# Meta-analysis of GWAS of over 16,000 individuals with autism spectrum disorder highlights a novel locus at 10q24.32 and a significant overlap with schizophrenia

**DOI:** 10.1186/s13229-017-0137-9

**Published:** 2017-05-22

**Authors:** The Autism Spectrum Disorders Working Group of The Psychiatric Genomics Consortium, Richard J. L. Anney, Stephan Ripke, Verneri Anttila, Jakob Grove, Peter Holmans, Hailiang Huang, Lambertus Klei, Phil H Lee, Sarah E. Medland, Benjamin Neale, Elise Robinson, Lauren A. Weiss, Lonnie Zwaigenbaum, Timothy W. Yu, Kerstin Wittemeyer, A.Jeremy Willsey, Ellen M. Wijsman, Thomas Werge, Thomas H. Wassink, Regina Waltes, Christopher A. Walsh, Simon Wallace, Jacob A. S. Vorstman, Veronica J. Vieland, Astrid M. Vicente, Herman vanEngeland, Kathryn Tsang, Ann P. Thompson, Peter Szatmari, Oscar Svantesson, Stacy Steinberg, Kari Stefansson, Hreinn Stefansson, Matthew W. State, Latha Soorya, Teimuraz Silagadze, Stephen W. Scherer, Gerard D. Schellenberg, Sven Sandin, Stephan J. Sanders, Evald Saemundsen, Guy A. Rouleau, Bernadette Rogé, Kathryn Roeder, Wendy Roberts, Jennifer Reichert, Abraham Reichenberg, Karola Rehnström, Regina Regan, Fritz Poustka, Christopher S. Poultney, Joseph Piven, Dalila Pinto, Margaret A. Pericak-Vance, Milica Pejovic-Milovancevic, Marianne Giørtz Pedersen, Carsten Bøcker Pedersen, Andrew D. Paterson, Jeremy R. Parr, Alistair T. Pagnamenta, Guiomar Oliveira, John I. Nurnberger, Merete Nordentoft, Michael T. Murtha, Susana Mouga, Preben Bo Mortensen, Ole Mors, Eric M. Morrow, Daniel Moreno-De-Luca, Anthony P. Monaco, Nancy Minshew, Alison Merikangas, William M. McMahon, Susan G. McGrew, Manuel Mattheisen, Igor Martsenkovsky, Donna M. Martin, Shrikant M. Mane, Pall Magnusson, Tiago Magalhaes, Elena Maestrini, Jennifer K. Lowe, Catherine Lord, Pat Levitt, Christa Lese Martin, David H. Ledbetter, Marion Leboyer, Ann S. LeCouteur, Christine Ladd-Acosta, Alexander Kolevzon, Sabine M. Klauck, Suma Jacob, Bozenna Iliadou, Christina M. Hultman, David M. Hougaard, Irva Hertz-Picciotto, Robert Hendren, Christine Søholm Hansen, Jonathan L. Haines, Stephen J. Guter, Dorothy E. Grice, Jonathan M. Green, Andrew Green, Arthur P. Goldberg, Christopher Gillberg, John Gilbert, Louise Gallagher, Christine M. Freitag, Eric Fombonne, Susan E. Folstein, Bridget Fernandez, M. Daniele Fallin, A. Gulhan Ercan-Sencicek, Sean Ennis, Frederico Duque, Eftichia Duketis, Richard Delorme, Silvia DeRubeis, Maretha V. DeJonge, Geraldine Dawson, Michael L. Cuccaro, Catarina T. Correia, Judith Conroy, Ines C. Conceição, Andreas G. Chiocchetti, Patrícia B. S. Celestino-Soper, Jillian Casey, Rita M. Cantor, Cátia Café, Jonas Bybjerg-Grauholm, Sean Brennan, Thomas Bourgeron, Patrick F. Bolton, Sven Bölte, Nadia Bolshakova, Catalina Betancur, Raphael Bernier, Arthur L. Beaudet, Agatino Battaglia, Vanessa H. Bal, Gillian Baird, Anthony J. Bailey, Marie Bækvad-Hansen, Joel S. Bader, Elena Bacchelli, Evdokia Anagnostou, David Amaral, Joana Almeida, Anders D. Børglum, Joseph D. Buxbaum, Aravinda Chakravarti, Edwin H. Cook, Hilary Coon, Daniel H. Geschwind, Michael Gill, Joachim Hallmayer, Aarno Palotie, Susan Santangelo, James S. Sutcliffe, Dan E. Arking, Bernie Devlin, Mark J. Daly

**Affiliations:** MRC Centre for Neuropsychiatric Genetics and Genomics, Cardiff University, Cardiff, CF24 4HQ UK

**Keywords:** Autism spectrum disorder, Genome-wide association study, Meta-analysis, Genetic correlation, Heritability, Gene-set analysis, Schizophrenia, Neurodevelopment

## Abstract

**Background:**

Over the past decade genome-wide association studies (GWAS) have been applied to aid in the understanding of the biology of traits. The success of this approach is governed by the underlying effect sizes carried by the true risk variants and the corresponding statistical power to observe such effects given the study design and sample size under investigation. Previous ASD GWAS have identified genome-wide significant (GWS) risk loci; however, these studies were of only of low statistical power to identify GWS loci at the lower effect sizes (odds ratio (OR) <1.15).

**Methods:**

We conducted a large-scale coordinated international collaboration to combine independent genotyping data to improve the statistical power and aid in robust discovery of GWS loci. This study uses genome-wide genotyping data from a discovery sample (7387 ASD cases and 8567 controls) followed by meta-analysis of summary statistics from two replication sets (7783 ASD cases and 11359 controls; and 1369 ASD cases and 137308 controls).

**Results:**

We observe a GWS locus at 10q24.32 that overlaps several genes including *PITX3*, which encodes a transcription factor identified as playing a role in neuronal differentiation and *CUEDC2* previously reported to be associated with social skills in an independent population cohort. We also observe overlap with regions previously implicated in schizophrenia which was further supported by a strong genetic correlation between these disorders (Rg = 0.23; *P* = 9 × 10^−6^). We further combined these Psychiatric Genomics Consortium (PGC) ASD GWAS data with the recent PGC schizophrenia GWAS to identify additional regions which may be important in a common neurodevelopmental phenotype and identified 12 novel GWS loci. These include loci previously implicated in ASD such as *FOXP1* at 3p13, *ATP2B2* at 3p25.3, and a ‘neurodevelopmental hub’ on chromosome 8p11.23.

**Conclusions:**

This study is an important step in the ongoing endeavour to identify the loci which underpin the common variant signal in ASD. In addition to novel GWS loci, we have identified a significant genetic correlation with schizophrenia and association of ASD with several neurodevelopmental-related genes such as *EXT1*, *ASTN2*, *MACROD2*, and *HDAC4.*

**Electronic supplementary material:**

The online version of this article (doi:10.1186/s13229-017-0137-9) contains supplementary material, which is available to authorized users.

## Background

Autism spectrum disorder (ASD) is diagnosed in roughly 1% of the population [1, 2] and has complex genetic roots. The recurrence risk of developing ASD in siblings of an affected individual is approximately 7–19% [3–5], and estimates of heritability are high from both twin (64−91%) [6] and whole genome genotyping studies (31−71%) [7]. Analysis of rare and de novo structural and sequence variation in ASD has had recent success in identifying genes and the biology underpinning ASD, albeit with direct relevance to only a small proportion of cases. The establishment of a number of robust risk genes such as *CHD8*, *GRIN2B*, *SCN2A*, and *SYNGAP1* [8], and gene-set analyses from associated structural variation have identified synaptic functioning, chromatin remodelling, Wnt signalling, transcriptional regulation, fragile X mental retardation protein (FMRP) interactors and, more broadly, MAPK signalling, as putative biological processes that are disrupted in ASD [9–13].

Importantly, common genetic variation explains roughly half of this genetic risk in ASD [7], making the genome-wide association study (GWAS) an efficient design for identifying risk variants. Early GWAS [12, 14–17] were performed using a variety of genotyping arrays, and the independent samples sizes were of low statistical power to robustly identify genome-wide significant (GWS) loci at the lower effect sizes (OR <1.15) [18]. Recently, large-scale coordinated international collaborations have been established to combine independent genotyping data to improve statistical power, a strategy that has been fruitful for both schizophrenia [19] and bipolar disorder [20]. In this study, we report the first meta-analyses of a coordinated international effort in ASD from the ASD Working Group of the Psychiatric Genomics Consortium (PGC). By combining published and unpublished GWAS data, we are now able to provide more robust estimates of the underlying common variant structure.

In addition to identifying risk loci, we have examined whether gene-sets previously implicated in ASD are similarly impacted with associated common genetic risk variants. The converging evidence across the variant spectrum should reinforce and expand our understanding of ASD biology. To uncover new biology, we have also examined enrichment of association across numerous functional and cellular annotations, as well as within canonical gene-sets.

Finally, evidence that common structural variation is shared by individuals with ASD, schizophrenia and intellectual disability (ID) continues to fuel a common biological model of ID-ASD-schizophrenia [21]. For example, FMRP biology has also been implicated in all three diagnoses [11]. The hypothesis of a shared pathophysiology for neurodevelopmental disorders is not novel, with Craddock and Owen [22] suggesting that autism exists along a continuum between mental retardation (intellectual disability (ID)) and schizophrenia. Using results from the PGC Schizophrenia Working Group GWAS [19], we have directly tested the relationship between ASD and schizophrenia and extended the meta-analyses by combining these data to identify neurodevelopment-related variants implicated across disorders.

## Methods

### Participants

Using meta-analysis, we examined association from 14 independent cohorts contributed by eight academic studies (see Table 1). Each contributing site confirmed all affected individuals had an ASD diagnosis; details of diagnostic processes are provided in the Additional file 1 and where available, study specific details are described elsewhere [7, 12, 16, 17, 23–25]. Where data permitted, we excluded individuals assessed at under 36 months of age or if there was any evidence of diagnostic criteria not being met from either the Autism Diagnostic Interview-Revised (ADI-R) [26] or the Autism Diagnostic Observation Schedule (ADOS) [27]. The primary meta-analysis (Worldwide ancestry (WW)) was based on data from 7387 ASD cases and 8567 controls. An additional meta-analysis on a more ancestrally homogenous subset (see Additional file 1) was also performed; this subset included data from 6197 ASD cases and 7377 controls of ‘European ancestry’.

**Table 1:** Study design and sample size of the contributing ASD collections. For some collections, more than one genotyping panel was used or the study design differed, i.e., trios or case-control; in such cases, the sample was split into ‘sets’ based on genotyping array and design

Study name	Set	Design	Sample size		M:F ratio	% Euro
			Case	Control		
Autism Center of Excellence Network (ACE)		Trios	372	372^a^	3.1:1	82.1
Autism Genetic Resource Exchange (AGRE)	[1]	Trios	572	572^a^	3.5:1	100.0
	[2]	Trios	1045	1045^a^	3.9:1	86.1
Autism Genome Project (AGP)	[1]	Trios	1259	1259^a^	5.4:1	95.9
	[2]	Trios	941	941^a^	8.6:1	84.4
Finnish Case-Control ASD Collection		CaCo	159	526	3.3:1	99.7
NIMH Repository and Montreal/Boston (MonBos) Collection		Trios	117	117^a^	4.1:1	95.5
Population-Based Autism Genetics and Environment Study (PAGES)		CaCo	305	1200	2.2:1	100.0
Simons Simplex Collection (SSC)	[1]	Trios	396	396^a^	7.1:1	86.2
	[2]	Trios	617	617^a^	6.2:1	84.7
	[3]	Trios	804	804^a^	5.9:1	82.0
	[4]	Trios	372	372^a^	7.5:1	87.8
Weiss Laboratory Autism Collection	[1]	CaCo	331	249	7.2:1	100.0
	[2]	Trios	97	97^a^	1.2:1	100.0
Total			7387	8567	4.9:1	90.8

^a^For trio designs, the control individuals are pseudocontrols generated from non-transmitted alleles. M:F ratio proportions derived from case-only; % Euro is an approximation defined as similarity to reference genotypes from 1000 genomes project (see Additional file 1). All sample sizes reported are post-imputation quality control

We sought independent replication of our results using summary GWAS findings from two additional sources; the Danish iPSYCH Project (7783 ASD cases and 11359 controls) and a combined deCODE Collection (from Iceland plus a collection of individuals from Ukraine, Georgia and Serbia) and the ‘Study to Explore Early Development’ (SEED) (1369 ASD cases and 137308 controls). A detailed description of each cohort is provided in the Additional file 1.

### Statistical analyses

#### Genotyping quality control

Genotyping quality control and imputation of the 14 independent cohorts were performed by the PGC Statistical Analysis Group. Each dataset was processed separately. Experimental details are described in the Additional file 1.

#### Association, meta-analyses, and binomial sign test

We tested all 14 cohorts individually for association under an additive logistic regression model using PLINK [28]. For samples derived from parent child trios, we applied a case-pseudocontrol design in which the pseudocontrol was created with the non-transmitted alleles from the parents. Since these pseudocontrols are perfectly matched to each case, no covariates were used in association analysis from these trio cohorts. For the non-trio cohorts, each regression included derived principal components as covariates [29]. To minimise the putative reduction in power observed when non-confounding covariates are included in GWAS for rare disease [30], sex was not included as a covariate in these analyses. Individual PP plots for each cohort are reported in the Additional file 1: Figure S1).

We performed a meta-analysis of the individual GWAS using an inverse-weighted fixed effects model [31] implemented in METAL (http://csg.sph.umich.edu//abecasis/Metal/) [32]. A fixed effects meta-analysis was chosen over a random effects model to maximise power and improved discover of associated SNPs [33]. An additional meta-analysis was performed including 13 of the cohorts, omitting the Swedish PAGES collection, this was named noSWM3. The Swedish PAGES collection include control individuals that overlap with the PGC schizophrenia GWAS, and we wished to preclude any potential for confounding of our results which rely upon comparison of these datasets. We performed a cross-disorder meta-analysis of the noSWM3 ASD GWAS and the PGC schizophrenia GWAS [19] using an inverse-weighted fixed effects model as described above. We applied a GWS threshold of *P* = 5 × 10^−8^. This is based on the Bonferroni approach, controlling the observed associations at *P* = 0.05 given approximately 1000000 independent tests.

To aid interpretation, we report findings as linkage disequilibrium (LD) independent SNPs and the corresponding ranges attributed to the LD. LD pruning was performed using the clump flag in PLINK v1.9 [28, 34]. Clumping was used to link additional associated markers within a 0.5Mb window surrounding the primary association. Markers were linked if they were also associated at P < .05 and had an estimated LD with the index SNP of r^2^ ≥ 0.2. Associated regions were defined for each index SNP as the location spanning all linked markers. All LD statistics were calculated using the 1000 genomes project phase 1 integrated reference haplotypes.

Binomial sign tests to evaluate the random direction effect between studies were implemented in STATA (version 13, Statacorp, College Station, TX, USA).

#### Gene-based and gene-set analyses

Combining association signals for multiple loci across genes and gene-sets has the potential to capture a greater proportion of variance leading to an increase in power [35]. We performed gene-based association analyses, using the VEGAS2 method (performed at: https://vegas2.qimrberghofer.edu.au/) [36]. This method calculates a test statistic from the sum of test statistics within a gene region. The LD between markers within a gene region is calculated and adjusted for within the software using the 1000 genomes reference genotypes. Analyses were limited to the top 10%, by *P* value, of SNPs per gene, an approach which has been shown to give rise to higher sensitivity and lower false positive rates compared to other gene-set methods [37].

To explore the converging biology hypothesis of ASD we examined enrichment within gene-sets derived from previously implicated genes and pathways (candidate gene-set) using the Interval-based Enrichment Analysis Tool for Genome Wide Association Studies (INRICH) method [38]. To identify new biology, we examined gene-set enrichment using established canonical gene-sets including gene ontology and the Kyoto Encyclopedia of Genes and Genomes (KEGG)﻿ gene-sets. INRICH is a pathway-based GWAS tool that tests for enrichment of association signals against predefined gene-sets across independent genomic intervals. INRICH analysis was performed in interval mode. The interval mode describes an enrichment statistic *E* for each gene-set that is the number of intervals that overlap at least one target gene in the gene-set. The significance of *E* is approximated using permutation and an empirical *P* value is generated from a null set of intervals which match the interval size, overlapping gene, and SNP number to the original input intervals. A detailed description of the gene-set compositions for the candidate gene-set and canonical gene-sets is reported in the Additional file 1. A summary of the sources of each candidate gene-set are also reported in Additional file 1: Table S1.

#### LD score-based heritability

SNP-based heritability, genetic correlation estimation, and partitioned heritability analyses were performed using the LD score approach [39] (scripts available at https://github.com/bulik/ldsc). Partitioned heritability was performed to examine enrichment of the heritability estimates within SNPs annotated per functional classes based on gene structure (promoter, intron, coding, UTR) and regulatory elements (Histone and DNASE-I hypersensitivity sites). We also examined cell type-specific histone binding elements to identify whether enrichment was limited to specific cell and tissue types. Finally, we applied the partitioned heritability method to examine whether enrichment existed in genes and gene-pathways previously implicated in ASD (see Additional file 1).

## Results

### Association analyses

Following quality control, the primary meta-analysis (Worldwide ancestry (WW)) included data on 6695386 common variants across all chromosomes (1−22, *X*) (minor allele frequency, MAF >0.05; imputation quality score (INFO) >0.60). The secondary GWAS, restricted to individuals defined as being of European (EUR) ancestry, included data on 6632956 common variants that surpassed quality control criteria. Summary Manhattan and PP plots for each analysis are reported in Additional file 1: Figures S2 (WW) and S3 (EUR). We applied a genomewide significance threshold of *P* ≤ 5 × 10^−8^. None of the markers investigated exceeded this threshold in the WW meta-analysis. A summary table, containing details of linkage independent associations at *P* < 10^−4^ is reported in the Additional file 2 (WW) and Additional file 3 (EUR). Complete summary statistics for these analyses are available at https://www.med.unc.edu/pgc/results-and-downloads.

### Replication

Although none of the discovery markers exceeded the GWS threshold, we wanted to further test the existence of true positive signal in our top associated regions and sought replication in independent samples. Previously, when comparing the results of an early iteration of the PGC schizophrenia GWAS (PGC1) [40], which is of similar size to that of reported here for the PGC ASD GWAS, to more recent and larger PGC schizophrenia GWAS (PGC2) [19], we noted that of the association signals 5 × 10^−8^ ≤ *P* < 10^−4^ in the schizophrenia PGC1 study, over 10% (20 of 183 independent loci) were subsequently reported as GWS in the schizophrenia PGC2 analysis. Others have also observed this, revealing in their sample that a substantial minority of associations with borderline genome-wide significance represent replicable and possibly genuine associations [41].

**Fig. 1:**
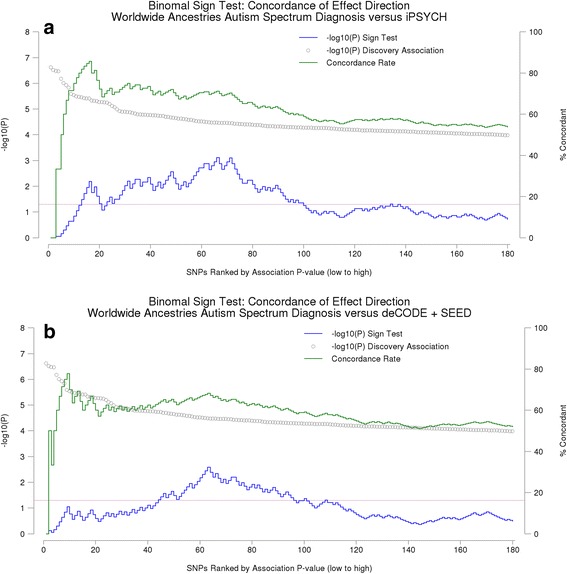
Sign test of concordance of the direction of effect (odds ratio) of the discovery (PGC worldwide) and the replication sample (**a** replication set 1: iPSYCH; **b** replication set 2: deCODE/SEED). *Blue line* is the –log_10_(*P*) of the binomial sign test for all associated markers below the rank. The *green line* describes the concordance, and the *grey markers* the association in the discovery set

Therefore, we sought replication of the primary PGC ASD GWAS (WW). Summary association data were obtained from the Danish iPSYCH ASD GWAS for all 180 LD-independent markers that were associated at *P* < 10^−4^. Of these, 11 (6.1%) met the nominal *P* < 0.05 threshold in the iPSYCH sample, a non-significant enrichment (Pr(*K* > = 11/180) = 0.29 where *K* is the number with *P* < 0.05). A step-wise binomial sign test was then performed to evaluate the concordance of direction of the effect for each pair of markers below a given rank. This analysis revealed significant enrichment for markers ranked within the top 100 associations (see Fig. 1a). Of the 11 (of 180) markers that were nominally associated in the Danish iPSYCH ASD GWAS, all ranked within the top 100 PGC ASD GWAS association signals (see Additional file 4).

A second replication set from the deCODE/SEED ASD GWAS was also available. Due to platform differences, information from only 159 of the 180 LD-independent markers was available. From these 159 markers, 8 (5%) resulted in association exceeding the nominal *P* < .05 (Pr(*K* > = 8/159 = 0.54). The step-wise binomial sign test revealed a smaller concordance effect, with the maximum concordance achieved with approximately the top 70 ranked PGC ASD GWAS association signals, again all nominally associated markers reside within this set (see Fig. 1b).

**Fig. 2:**
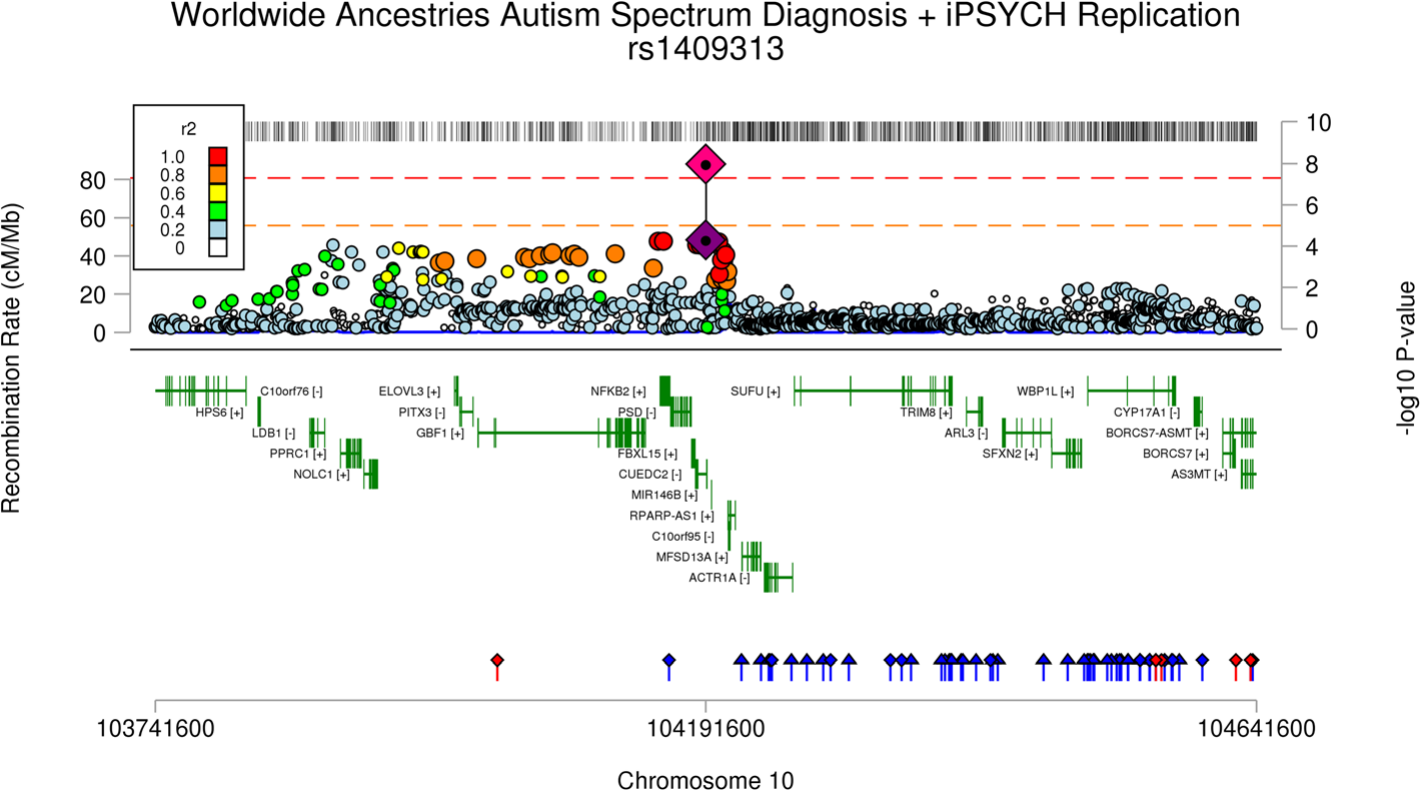
Association locus plot for the index SNP rs1409313 in the GWAS of all (worldwide) ancestries autism spectrum disorder. The GWS association (*pink diamond*) refers to the combined PGC-iPSYCH meta-analyses. Additional panels include gene location and location of eQTL markers

When the top association signals from the PGC ASD GWAS (*P* < 10^−4^) were meta-analysed against the Danish iPSYCH data a single GWS association was observed for rs1409313-T (OR = 1.12 (95% CI 1.08–1.16); *P* = 1.058 × 10^−8^). This marker has previously been implicated as a paternally inherited risk marker for ASD within the Simons Simplex Collection (SSC) data [25]. The SSC is not independent as the PGC ASD GWAS data as these individuals are included within our analyses. Examination of the LD between adjacent associated markers and rs1409313 using the clump routine in PLINK, reveals that rs1409313 is correlated with nominally associated markers across a 405 kb region on chromosome 10q24.32 that includes 13 genes (*C10orf76*, *CUEDC2*, *ELOVL3*, *FBXL15*, *GBF1*, *HPS6*, *LDB1*, *MIR146B*, *NFKB2*, *NOLC1*, *PITX3*, *PPRC1*, and *PSD*). A summary linkage disequilibrium locus plot of these data is shown in Fig. 2.

Meta-analysis of the PGC ASD—deCODE/SEED data did not result in any GWS findings. However, the top-ranked loci do identify genes previously implicated in ASD such as *EXOC4* [42], *ANO4* [43], *EXT1* [44], and *ASTN2* [45]. Similarly, a combined meta-analysis of both PGC plus iPSYCH plus deCODE/SEED datasets did not identify markers exceeding the GWS threshold. The top-ranked locus resulting from this analysis was again rs1409313-T, albeit not achieving GWS (OR = 1.10 (95% CI 1.06–1.14); *P* = 1.47 × 10^−6^). In addition to rs1409313-T, the top-ranked associations include markers tagging *HDAC4*, *MACROD2*, and *EXOC4*. A summary of the meta-analysis results is provided in the Additional file 4.

### SNP-heritability estimate

We performed LD-score regression to determine the additive heritability attributed to the genome-wide SNPs. The heritability from the WW sample on the observed scale is 0.326 (SE = 0.043, unconstrained, intersect 0.97 (0.007), liability scale *h* ^2^ (assuming population prevalence of 1%; 0.18 (SE = .02)). This estimate was based on data from 1095,173 high-confidence SNPs, which show an association inflation, Lambda = 1.06. For the European ancestry samples, the heritability estimate is nearly identical (1,081,358 SNPs; observed scale *h* ^2^ = 0.334 (SE = 0.035; unconstrained; intersect 0.99 (0.008); liability scale *h* ^2^ (assuming population prevalence of 1%; 0.19 (SE = .02); Lambda = 1.07)). Both estimates are consistent with previous PGC-based estimates of SNP heritability from a subset of these data (liability scale *h* ^2^ = 0.17 (SE = 0.025) [46]).

Using the noSWM3 ASD data and the summary GWAS data from the PGC schizophrenia study, we estimate the genetic correlation between ASD and schizophrenia at approximately 23% (genetic correlation (Rg) = 0.23 (SE = 0.05); *P* = 9 × 10^−6^; total liability scale genetic covariance = 0.09 (SE 0.02)). This genetic covariance is almost threefold larger than previous reports [47]. As a null comparison, we estimated the genetic correlation against the unrelated Rheumatoid Arthritis GWAS and found no significant correlation with ASD.

### Gene-based and gene-set analyses

Gene-based association, performed using the VEGAS2 algorithm, tested the significance of 17,322 genes (Bonferroni threshold *P* < 2.89 × 10^−6^) (see Additional file 5). No gene-level associations were significant after Bonferroni correction. The minimum *P* value achieved, *P* = 7 × 10^−6^, was for 3 genes from chromosome 6p21.1 (*ENPP4*, *ENPP5*, and *CLIC5*) and was driven by association of rs7762549, the 4th ranked association interval. To the best of our knowledge, none of these genes have previously been implicated in ASD or ASD-related traits.

Association enrichment was performed using the INRICH method [38]. Using the WW data, we observe enrichment at an empirical *P* ≤ .05 for the synaptic co-expression network M13 [48], Mendelian disease genes [11], and both human (HARs) and primate-accelerated regions (PARs) [49]. None of these enrichments exceeded the experiment-wise corrected *P* ≤ .05. Due to overlapping samples between the ASD and PGC GWAS, INRICH analyses against the PGC schizophrenia study [19] was restricted to the noSWM3 ASD GWAS including the worldwide dataset minus the Swedish PAGES sample. This set was analysed in isolation and exceeded the experiment-wise correction (*P* = .008), with 19 of the 82 blocks included in the analysis overlapping these annotations. Finally, none of the 9708 canonical gene-sets examined in these analyses met the experiment-wise corrected *P* ≤ .05. A full summary of the INRICH analyses and associated files are given in the Additional files 6, 7, 8, 9, 10, 11, and 12. Of the top-ranked enrichments from canonical pathways, some of the gene-sets overlap those previously highlighted in ASD biology and include processes such as glutamate receptor activity, adheren/cell junctions and the beta-catenin nuclear pathway.

Using the LD Score approach, we also estimated the proportion of heritability that can be attributed to specific partitions of the genome, such as those attributed to gene-sets [39]. Analyses were divided into two sets; WW ASD GWAS against candidate gene-sets, functional annotations and cell type annotation and noSWM3 ASD GWAS against PGC schizophrenia GWS loci. A summary of observed enrichment at an uncorrected *P* ≤ .05 is given in Table 2, full enrichment data are provided in the Additional file 13. We again observe evidence of an overlap with schizophrenia, with a 2.5-fold enrichment in heritability for those markers within the PGC schizophrenia GWS loci (*P* = 0.021). The most significant enrichments were observed for annotations tagging gene enhancers, conserved elements and histone marks indexing expression in the mid-frontal lobe. We do not observe evidence for FMRP targets in either the INRICH or LD score analyses.

**Table 2:** Enriched heritability by functional, cellular, and candidate gene-set annotations. prSNPs refers to proportion of SNPs in the model, prH2 refers to proportion on the heritability (SE) attributed to the annotation set, and enrichment refers to the enrichment (SE; *P* value) in heritability given the number of SNPs in the model

Category	prSNPs	prH2	Enrichment
Function: weak enhancer +/−500 bp	0.106	0.299 (0.06)	2.82 (0.61); *P* = 0.0045
Function: conserved	0.052	0.258 (0.08)	4.94 (1.46); *P* = 0.0047
Cell: CNS: mid-frontal lobe: H3k27ac	0.027	0.132 (0.04)	4.89 (1.34); *P* = 0.0056
Function: enhancer +/−500 bp	0.180	0.402 (0.08)	2.24 (0.44); *P* = 0.0065
Function: DNASE I hypersensitivity site (foetal) +/−500 bp	0.362	0.666 (0.11)	1.84 (0.32); *P* = 0.0099
Candidate: Mendelian disease [11] genes	0.018	0.040 (0.01)	2.19 (0.49); *P* = 0.0132
Candidate: PGC schizophrenia GWAS loci [19]	0.014	0.035 (0.01)	2.50 (0.70); *P* = 0.0211
Function: H3k4me1	0.538	0.771 (0.10)	1.43 (0.19); *P* = 0.0303
Function: CCCTC-binding factor	0.027	0.129 (0.05)	4.71 (1.75); *P* = 0.0326
Cell: CNS: hippocampus middle: H3k4me1	0.077	0.211 (0.06)	2.76 (0.81); *P* = 0.0351
Cell: CNS: angular gyrus: H3k27ac	0.033	0.106 (0.03)	3.25 (1.03); *P* = 0.0365
Function: digital genomic footprint +/−500 bp	0.621	0.836 (0.10)	1.35 (0.17); *P* = 0.0399
Function: conserved +/−500 bp	0.449	0.603 (0.07)	1.34 (0.17); *P* = 0.0488

### Overlap of ASD and schizophrenia GWAS

In addition to the significant genetic correlation between ASD and schizophrenia, and enrichment in the heritability, we see further support for an etiological overlap when considering the PGC ASD data as a ‘replication set’ for schizophrenia. When considering the GWS loci reported in the PGC schizophrenia GWAS, 118 of the markers pass QC in the ASD WWW GWAS sample. Eleven of these 118 schizophrenia-associated markers are also associated with ASD at *P* ≤ .05 (Pr(*K* > = 11/118) = 0.035). Moreover, when applying the binomial sign test, we observe strong concordance of the direction of these markers (concordance 64.4%; Pr(*K* > = 76/118) = 0.0011) (see Additional file 1: Figure S14 Panel A). As a comparison, we did not observe any similar enrichment with a Rheumatoid Arthritis dataset (see Additional file 1: Figure S14 Panel B).

Given these observations regarding ASD and schizophrenia, and to further identify novel loci, we meta-analysed the PGC schizophrenia and PGC ASD GWAS data (see Additional file 1: Figure S12 for Manhattan and PP plot). After removing significant loci (+/−1 Mb) previously reported in the PGC schizophrenia analyses, we observed 12 new GWS loci (see Table 3; Additional file 14 and Additional file 15).

**Table 3:** Novel GWS loci from combined ASD-schizophrenia GWAS

SNP	Locus range	A1	Odds ratio (95% CI)			*P* value	Genes within locus
			ASD	SCZ	Combined		
rs57709857	chr8:38014429..38316849	A	0.92 (0.86–0.97)	0.93 (0.91–0.96)	0.93 (0.91–0.95)	4.2 × 10^−9^	BAG4 DDHD2 FGFR1 LETM2 LSM1 PLPP5 WHSC1L1
rs1353545	chr3:60276185..60305117	C	1.05 (1.00–1.10)	1.06 (1.04–1.09)	1.06 (1.04–1.08)	1.1 × 10^−8^	FHIT
rs6803008	chr3:71433554..71679148	T	0.94 (0.90–0.99)	0.95 (0.93–0.97)	0.94 (0.93–0.96)	1.3 × 10^−8^	FOXP1 MIR1284
rs2828478	chr21:25092482..25219939	A	1.07 (1.02–1.12)	1.06 (1.04–1.08)	1.06 (1.04–1.08)	1.6 × 10^−8^	None
rs9879311	chr3:10317432..10520739	T	1.08 (1.03–1.13)	1.05 (1.03–1.08)	1.06 (1.04–1.08)	1.9 × 10^−8^	ATP2B2 GHRL GHRLOS LINC00852 MIR378B MIR885 SEC13 TATDN2
rs73416724	chr6:43234901..43411659	A	1.11 (1.03–1.20)	1.09 (1.05–1.13)	1.09 (1.06–1.13)	3.0 × 10^−8^	ABCC10 CRIP3 MIR6780B SLC22A7 TTBK1 ZNF318
rs61847307	chr10:53935082..54035437	T	0.95 (0.90–1.00)	0.94 (0.92–0.96)	0.94 (0.92–0.96)	3.1 × 10^−8^	PRKG1
rs7122181	chr11:81178475..81209569	A	0.95 (0.91–1.00)	0.95 (0.93–0.97)	0.95 (0.93–0.97)	3.7 × 10^−8^	None
rs880446	chr20:62113220..62178105	A	1.07 (1.02–1.13)	1.06 (1.04–1.09)	1.06 (1.04–1.09)	4.4 × 10^−8^	EEF1A2 PPDPF PTK6 SRMS
rs7521492	chr1:163581663..163790947	A	1.05 (1.00–1.10)	1.06 (1.03–1.08)	1.06 (1.04–1.08)	4.7 × 10^−8^	None
rs72986630	chr19:11849736..11943697	T	1.07 (0.95–1.21)	1.16 (1.10–1.22)	1.14 (1.09–1.20)	4.7 × 10^−8^	ZNF440 ZNF441 ZNF491 ZNF823
rs4904167	chr14:84628384..84701798	T	1.08 (1.03–1.14)	1.05 (1.03–1.07)	1.06 (1.04–1.08)	4.9 × 10^−8^	None

## Discussion

### Genomewide association study of ASD

We present here the results from a large international collaborative GWAS meta-analysis and follow up of 16539 ASD cases and 157234 controls. Despite the considerable increase in the sample size and statistical power of these new analyses to identify associations, we do not observe individual variants that exceed the accepted GWS threshold (*P* ≤ 5 × 10^−8^) in the discovery GWAS (*n* = 7387 ASD cases and 8567 controls). This does not, however, disqualify the loci which fall within the upper-ranked associations from further interrogation, through replication or through supporting biology. There is evidence to support the hypothesis that a substantial proportion of ‘borderline’ association represent genuine associations and deserve an attempt at replication [41]. A PGC schizophrenia GWAS [40], which was of comparable size to this, yielded 5 GWS loci and 183 non-GWS loci at *P* < 10^−4^. In a follow-up study [19], 20 of these markers were elevated beyond GWS; these were not limited to the most highly ranked markers, as the newly GWS markers ranged from 7 to 188 in the original study.

We have sought to assess the veracity of these upper-ranked associations through replication and meta-analysis using summary GWAS findings from the Danish iPSYCH Project (*n* = 7783 ASD cases and 11359 controls) and the combined deCODE and SEED collection (*n* = 1369 ASD cases and 137308 controls). These analyses show that the concordance of the direction of effect is highly significant for the top 100 and top 70 markers, for the iPSYCH and deCODE/SEED ASD datasets, respectively.

The combined meta-analysis of the PGC ASD GWAS and iPSYCH samples show a single GWS association at rs1409313-T, a marker located on chromosome 10q24.32 within intron 1 of *CUEDC2. CUEDC2* encodes the protein ‘CUE domain containing 2’, shown to be involved in the ubiquitination-proteasomal degradation pathway. Others have previously identified associations at this region with the social skills domain of the autism quotient in a population cohort (independent of the PGC ASD GWAS [50]); identifying an association with rs927821 (*P* = 3.4 × 10^−6^), a marker in strong linkage disequilibrium with rs1409313 (1000 genomes project all ancestries: *r* ^2^ = 0.82; *D*’ = 0.91; European ancestries: *r* ^2^ = 0.68; *D*’ = 0.85). Other genes in this region include *PITX3* which encodes the transcription factor Pitx3 (paired-like homeodomain 3) which plays an important role in the differentiation and maintenance of midbrain dopaminergic neurons during development. It is important to note that the PGC ASD association does not overlap with the 10q24.32 association observed in the recent PGC schizophrenia GWAS. The ASD signal occurs directly upstream of the schizophrenia signal and is separated by a recombination hotspot (see Additional file 1: Figure S13). Other notable genes within the top-ranked associations observed in the PGC-iPSYCH meta-analysis are *HDAC4* and *MACROD2*. GWS associations have previously been reported for *MACROD2* by the Autism Genome Project, a subset of the data included in these analyses [12]. *HDAC4* encodes the gene histone deacetylase 4 and is involved in the deacetylation of core histones. Dosage of *HDAC4* has been implicated in ASD, with observed overexpression in the post-mortem brain tissue of individuals with ASD [51]; conversely, deletions of the *HDAC4* loci have been reported in individuals with syndromic autism [52]. Annotation of the associated region implicated in this study (chr2:240183467 to 240327863) does not reveal eQTLs that may support a functional link (see Additional file 2).

The meta-analysis of the PGC–deCODE/SEED data failed to identify GWS SNPs. However, the top-ranked findings did identify genes previously implicated in ASD such as *EXOC4* [42], *ANO4* [43], *EXT1* [44, 53], and *ASTN2* [45]. *ASTN2* was implicated because of association with the marker rs7026354. Although relatively modest in this meta-analysis, rs7026354 is reported as the 10th ranked association in the PGC-deCODE/SEED ASD analysis (OR = 1.10 (95% CI 1.06–1.14); *P* = 4.96 × 10^−6^), this marker is noteworthy because it passes the GWS threshold in the European ancestry only data (rs7026354-A; OR = 1.15 (95% CI 1.09–1.20); *P* = 4.99 × 10^−8^ (see Additional file 1: Figure S9 for corresponding locus plot). *ASTN2* encodes the protein, astrotactin 2 (ASTN2). Astrotactin 1 (ASTN1) is a membrane protein which forms adhesions between neurons and astroglia [54]. ASTN2 interacts with ASTN1, regulating its expression, thereby playing a role in neuronal-glial adhesion during migration [55]. *ASTN2* has previously gained recognition due to the presence of rare CNV losses in ASD [56, 57]. In a recent study of 89,985 individuals, approximately 71% of whom were reported to have a neurodevelopmental disorder (NDD), a total of 46 deletions and 12 duplication mutations were observed in the *ASTN2* gene [58]. Exonic deletions were significantly enriched in the NDD cohort (OR = 2.69 (95% CI 1.13–7.77); *P* = 0.01). The peak of our association signal at 9q33.1 extends over the 3’ end of *ASTN2* corresponding to a region of the gene that encodes the C terminus of the protein, a region with cross-species sequence conservation and strong enrichment of exonic deletions in male NDD cases [58].

To garner additional information regarding the biology of ASD, we explored enrichment of association signals across a range of gene-sets and genomic annotations. Of the top canonical results, we observed enrichment in gene-sets related to synaptic structure and function. PDZ domain-binding, beta-catenin nuclear pathways, glutamate receptor activity, and adherens junctions all fit this categorization. PDZ domains (PSD95-disc large-zonula occludens-1) are found in scaffolding proteins, and those at neuronal excitatory synapses are thought to play a key role in synapse organisation and structure. These proteins organise glutamate receptors and associated protein composition at the synapse, subsequently determining the size and strength of the synapse (reviewed in [59]). Moreover, the beta-catenin and adherens gene-sets describe proteins that are involved in establishing synaptic contacts. Genetic insult which disrupts the synapse as a model of ASD is not novel [60], with genes encoding members of the SHANK, neurexin and neuroligin families, all well established as risk factors for ASD. Our data implicating gene-sets that interact with these genes confer additional support for the synaptic hypothesis of ASD.

The candidate gene-set analysis demonstrates a consistent relationship between schizophrenia and ASD. We observed strong genetic correlation between ASD and schizophrenia and a striking concordance in terms of direction of effect (binomial sign test of effect). Moreover, when examining only those genes within the schizophrenia GWS loci, we observed enrichment through gene-based and partitioned heritability analyses. We have excluded confounding due to known sample overlap.

### Cross-disorder genome-wide association study of ASD and schizophrenia

We extended our analyses to perform a cross-disorder ASD and schizophrenia meta-analyses to identify putative neurodevelopmental loci; again, we utilised the ASD GWAS excluding known shared controls. We identified 12 GWS loci not previously identified as GWS in the PGC schizophrenia GWAS.

The strongest independent locus, rs57709857-A (OR 0.93, 95% CI 0.91–0.95; *P* = 4.15 × 10^−9^) identifies a region previously branded as a neurodevelopmental hub on chromosome 8p (8p11.23). Other GWS associations include the marker rs9879311-T, indexing an association signal within the *ATP2B2* gene located at 3p25.3 (OR 1.06, 95% CI 1.04–1.08; *P* = 6.04 × 10^−9^) and rs6803008-T, indexing the *FOXP1* locus at 3p13 (OR 0.94, 95% CI 0.93–0.96; *P* = 1.34 × 10^−8^) (see Additional file 1: Figure S16 for corresponding locus plots). *ATP2B2* (ATPase, Ca(2+)-transporting, plasma membrane, 2) plays an important role in intracellular calcium homeostasis and has previously been implicated in ASD through reported genetic associations in North American, Italian, and Chinese samples [61–63], as well as through differential expression in ASD brain tissue [64]. *FOXP1*, a member of the Forkhead Box P family of transcription factors has been implicated in ASD aetiology based on observations of multiple de novo SNVs [65–67]. *FOXP1* has also been implicated in several related cognitive phenotypes including language impairment and intellectual disability [68–70]. Moreover, in a murine *Foxp1* KO mouse model, in addition to observable neuronal phenotypes, mice exhibit many behavioural phenotypes associated with ASD [71]. The genetic relationship we observed for common variation shared between schizophrenia and ASD is striking; alongside shared rare structural variation, such as that observed in the 22q11.2 deletion syndrome [72], these data suggest a common risk and a shared biology leading to related but distinct outcomes. We must also consider potential confounding; there is some evidence to support increased assortative mating within and across psychiatric illnesses. Consequently, the evidence from a cross-disorder meta-analysis may not be due to pleiotropy (or not entirely) but may instead be an artefact of a residual genetic background from assortative mating between individuals with these diagnoses [73]. Whether the observed degree of such assortative unions can explain the observed correlations will require further investigation.

To make further progress with the investigation of common variation in ASD, several strategies are being implemented. First, substantial increases in sample size are necessary. This is on the near horizon, with the ongoing activities from groups such as the iPSYCH collaborative likely to bring data from thousands of additional ASD cases to the GWAS effort. Secondly, genetic designs for studies of complex developmental disorders, including ASD and ADHD, have tended to favour a trio-based design and family-based association testing. Although this has provided strength in reducing artefacts resulting from population stratification, recent simulations advise against their use in common and complex polygenic traits, especially where assortative mating may be involved and where the family is known to be multiplex [74]. In such cases, the trio design can underestimate the SNP heritability and the power to observe association.

## Conclusions

This study provides an additional step towards understanding the genetic architecture of ASD. We show a robust relationship with the genetic risk identified in schizophrenia GWAS and have highlighted loci such as 10q24.32 and gene-sets that have been previously and independently implicated in ASD and related disorders. Shared heritability findings and our cross-disorder meta-analysis reveals additional GWS loci that may be important in neurodevelopment including a region flagged as a neurodevelopmental hub on chromosome 8p as well as the *ATP2B2* gene located at 3p25.3, a gene previously implicated in ASD through both genetic association [61–63] and differential expression in the post-mortem brain tissues of individuals with ASD [64].

Like other GWAS of similar size, our ASD-only results are not definitive with the observed associations falling short of accepted statistical significance thresholds. However, we view these data as an important step in the ongoing endeavour to identify the loci which underpin the common variant signal in ASD and we anticipate that some of these loci of ‘borderline’ significance, especially those with additional corroborating evidence such as *EXT1*, *ASTN2*, *MACROD2*, and *HDAC4*, to eventually garner sufficient evidence to become established robust ASD risk loci.

## Additional files

Additional file 1: Supplementary Text, Figures and Tables. (DOCX 13.3 MB)

Additional file 2: Summary GWAS finding for ASD Meta-analysis (World-wide Ancestries); Data reported on *P* < 10E-4. (XLS 99 KB)

Additional file 3: Summary GWAS finding for ASD Meta-analysis ("European" Ancestries); Data reported on *P* < 10E-4. (XLS 119 KB)

Additional file 4: Targetted Association Replication of Discovery Markers (ASD Meta-analysis (World-wide Ancestries): *P* <1e-4) in independent ASD cohorts. (XLS 5 MB)

Additional file 5: Summary Gene-based association findings (VEGAS2) for ASD Meta-analysis (World-wide Ancestries); Data reported on ALL and *P* < .05. (SETS 0.97 MB)

Additional file 6: INRICH-format gene-sets for "ASD Candidate Gene-sets". (SETS 39.2 MB)

Additional file 7: INRICH-format gene-sets for "Canonical Gene-sets". (GENES 601 KB)

Additional file 8: INRICH-format gene co-ordinates (based on hg19). (SETS 17 KB)

Additional file 9: INRICH-format gene-set derived from "PGC Schizophrenia GWAS - PMID:25056061". (XLS 490 KB)

Additional file 10: Full Results of Gene-set Enrichment findings (INRICH) for ASD Meta-analysis (World-wide Ancestries) versus Candidate.sets. (XLS 236 KB)

Additional file 11: Full Results of Gene-set Enrichment findings (INRICH) for ASD Meta-analysis (World-wide Ancestries; no Swedish PAGES) versus PGCSchizophrenia2014.sets. (XLS 1.48 MB)

Additional file 12: Full Results of Gene-set Enrichment findings (INRICH) for ASD Meta-analysis (World-wide Ancestries) versus Canonical.sets. (XLS 60 KB)

Additional file 13: Partitioned Heritability analysis (LDSC) for ASD Meta-analysis (World-wide Ancestries). (XLS 119 KB)

Additional file 14: Summary finding for ASD (World-wide Ancestries; no Swedish PAGES) and Schizophrenia (PMID: 25056061) Meta-analysis; Data reported on *P* ˂ 5E-8; all associations. (XLS 154 KB)

Additional file 15: Summary finding for ASD (World-wide Ancestries; no Swedish PAGES) and Schizophrenia (PMID: 25056061) Meta-analysis; Data reported on *P* ˂ 5E-8; all associations not previously reported at *P* ˂ 5E-8 in ASD or SCZ. (XLS 28 KB)

## Abbreviations

95% CI: 95% Confidence interval boundaries
ADI-R: Autism Diagnostic Interview-Revised
ADOS: Autism diagnostic observation schedule
ASD: Autism spectrum disorder
EUR: European ancestry
FMRP: Fragile X mental tetardation protein
GWAS: Genome-wide association study
GWS: Genome-wide significant
H2: Heritability
ID: Intellectual disability
INFO: Imputation Quality Score
LD: Linkage disequilibrium
MAF: Minor allele frequency
MAPK: Mitogen-activated protein kinase
noSWM3: ASD GWAS meta-analysis excluding the Swedish PAGES GWAS
OR: Odds ratio
PGC: Psychiatrics Genomics Consortium
Rg: Genetic correlation
SE: Standard error
UTR: Untranslated region of a transcript
WW: Worldwide ancestry
