# Supplementary material for: Meta-analysis of GWAS of over 16,000 individuals with autism spectrum disorder highlights a novel locus at 10q24.32 and a significant overlap with schizophrenia
Source: Mol Autism. 2017 May 22;8:21. doi: 10.1186/s13229-017-0137-9 (PMC5441062; doi:10.1186/s13229-017-0137-9)
Supplement: Supplementary file 1 — Supplementary Text, Figures and Tables. (DOCX 13.3 MB) [file 13229_2017_137_MOESM1_ESM.docx]

# Supplementary Information

Meta-analysis of GWAS of over 16,000 individuals with Autism Spectrum Disorder highlights a novel locus at 10q24.32 and a significant overlap with schizophrenia.

The Autism Spectrum Disorders Working Group of The Psychiatric Genomics Consortium

## Cohort Description

### Discovery Cohorts

We meta-analysed fourteen independent cohorts contributed by eight academic studies. Each contributing site confirmed all affected individuals had an ASD Diagnosis derived from research standard tools and expert clinical consensus diagnosis. Specific study details were described elsewhere [1-7]. When relevant data were provided, we excluded individuals if they were assessed at less than 36 months old or if there was any evidence of the diagnostic criteria not being met from either the Autism Diagnostic Interview-Revised (ADI-R) or the Autism Diagnostic Observation Schedule (ADOS) domain scores. The primary meta-analysis (Worldwide (WW)) was based on data from 7,387 ASD cases and 8,567 controls. An additional more ancestrally homogenous subset was also meta-analysed. The subset of subjects of European ancestry (EU) contained data from 6,197 ASD cases and 7,377 controls. We do not have detailed information on the family structure of the available individuals; specifically, whether the family is considered “Multiplex” or implicitly “Simplex”. This has relevance when considering the polygenic model of ASD; in a multiplex family, the genetic architecture favours the polygenic (common variant of small effect) and/or inherited (rare variant of large effect) models, whereas in a simplex family, the genetic architecture favours de novo mutation models. This has relevance to the power of the study to observe common variant associations. The study design applied by some of our contributing sites are implied, such as the Simons Simplex Collection; in our other collections, the family status is unknown or unavailable.

The contributing Discovery Cohorts are;

1. Autism Centre of Excellence (Geschwind Laboratory)
2. Autism Genome Project (AGP)
3. Autism Genetic Resource Exchange
4. Finnish Autism Collection
5. NIMH Repository and Montreal/Boston Collection
6. Population-based Autism Genetics and Environment Study (PAGES)
7. Simons Simplex Collection (SSC)
8. Weiss Laboratory Autism Collection

Ethics Approval Statement: All samples used in this study arose from investigations approved by the individual and respective Institutional Review Boards in the USA and at international sites where relevant. Informed consent was obtained for all adult study participants; for children under age 18, both the consent of the parents or guardians and the assent of the child were obtained.

#### Autism Center of Excellence

Families were drawn from the Autism Genetics Resource Exchange (AGRE) cohort [3] or recruited by the Geschwind Autism Centre of Excellence (ACE) Network consistent with protocols established by AGRE. Purified DNA from lymphoblastoid cell lines was obtained from the Rutgers University Cell and DNA Repository (RUCDR; Piscataway, NJ) and genotyped on the Illumina Omni-2.5 platform using standard manufacturer protocols (Illumina, San Diego, CA). Autism diagnoses were derived from a combination of assessment on the Autism Diagnostic Interview-Revised (ADI-R) [8] and/or Autism Diagnostic Observation Schedule (ADOS) [9] and clinician’s best judgment according to standard protocols at AGRE [3].

Additional Ethics Approval Statement: This study was approved by the Institutional Review Boards at AGRE, UCLA and Washington University.

#### Autism Genome Project

The Autism Genome Project (AGP) collection represents families collected from more than 50 centres across North America and Europe. Whole genome genotype information was collected and previously examined via GWAS [1, 2], CNV [10] [11] and linkage analyses [12]. The AGP collection is family-based, with a total of 1,259 and 941 probands included from the AGP stage 1 and AGP stage 2 collections, respectively. As part of the study exclusion criteria, subjects with known karyotypic abnormalities and fragile X mutations were excluded. Individuals were assessed using the Autism Diagnostic Interview-Revised (ADI-R) [8] and Autism Diagnostic Observation Schedule (ADOS) [9]. Cognitive functioning was established for most subjects using a range of cognitive measures from which a categorical classification of intellectual capacity was derived. Genotyping was performed by using either Illumina Human 1M-single Infinium BeadChip or Illumina Human 1M-Duo Infinium BeadChip arrays on Stage 1 and Stage 2, respectively. A detailed description of the genotyping procedure and sample is described elsewhere [1, 2],

Additional Ethics Approval Statement: Informed consent was obtained from all participants, and all procedures followed were in accordance with the ethical standards on human experimentation of the participating sites.

#### Autism Genetic Resource Exchange and NIMH Repository/Montreal/Boston Collection

This collection of parent, child trios was the foundation for the GWAS and linkage analyses described by Weiss and colleagues [5]. This collection was genotyped at the Broad Institute and the Johns Hopkins Center for Complex Disease Genomics on Affymetrix 5.0 and 500K platforms.

The Autism Genetic Resource Exchange (AGRE) collection [4] is a curated resource of DNA and phenotypic data from multiplex families with autism spectrum disorder (ASD) available for genetic research. A child was included in the analyses if he/she met criteria for autism by the ADI-R [8]. Probands were excluded if there was discrepancy in diagnosis between the ADI-R and ADOS. Study exclusion criteria included subjects with known karyotypic abnormalities and fragile X mutations and those with inconsistencies in genetic data (generating excess Mendelian segregation errors or showing genotyping failure on a test panel of 24 SNPs used to check gender and sample identity with the full array data).

The NIMH Autism Genetics Initiative (<https://www.nimhgenetics.org/available_data/autism/>) is a collection of DNA from multiplex and simplex families with ASD, that are independent of the AGRE dataset, with at least one child meeting criteria for autism by the ADI-R. Similar exclusion criteria to the AGRE collection were used, including known chromosomal abnormalities and excess non-Mendelian inheritance.

The Montreal subjects were diagnosed with ASD and recruited from clinics specializing in the diagnosis of Pervasive Developmental Disorders (PDD), readaptation centers, and specialized schools in the Montreal and Quebec City regions, Canada [13]. Study exclusion criteria include: subjects with estimated mental age < 18 months, a diagnosis of Rett syndrome or Childhood Disintegrative Disorder or evidence of any psychiatric and neurological conditions including: birth anoxia, rubella during pregnancy, fragile X syndrome, encephalitis, phenylketonuria, tuberous sclerosis, Tourette and West syndromes. Participants with a co-occurring diagnosis of semantic-pragmatic disorder, attention deficit hyperactivity disorder, and idiopathic epilepsy were eligible. Assessment of ASD was performed using the Autism Screening Questionnaire [14], followed by the ADI-R [8] and ADOS [9]. A detailed description of the genotyping procedure and sample is described elsewhere [5].

The Boston Sample consists of the Santangelo EDSP family sample and the Iranian Trio sample. The Santangelo EDSP families were ascertained for having one or more autistic children and at least one non-autistic child aged 16 or older for an extremely discordant sib-pair linkage study. Recruitment took place in Massachusetts and surrounding states through contacts with parent support and patient advocacy groups, brochures, newsletters, and the study web site. Parents were interviewed about their children, and non-autistic children were interviewed about themselves. An informant/caregiver, usually the proband’s mother, was interviewed using the Autism Diagnostic Instrument-Revised (ADI-R) to confirm the diagnosis of autism at age 4–5 years. Families were included if the affected children met Diagnostic and Statistical Manual of Mental Disorders-IV (DSM-IV) criteria for autistic disorder and their non-autistic siblings (aged 16 and older) did not display any of the broader autism phenotype traits, which were assessed with the (M-PAS-R), the Pragmatic Language Scale (PLS), and the Friendship Interview. Probands were excluded if they had medical conditions associated with autism such as fragile X syndrome or gross CNS injury, or if they were under four years of age, due to the possible uncertainty in diagnosis at younger ages. Twenty-nine families met eligibility criteria for the study and comprised the final sample for analysis. The eligible participants for the Iranian trio samples were Iranian families with at least one child affected with ASD, including cases of autistic disorder, Asperger syndrome and pervasive developmental disorder-not otherwise specified (PDD-NOS). Eighty families (282 individuals) from Iran were ascertained and assessed. This sample was ascertained by screening and diagnostic testing of over 90,000 preschool children from Tehran in 2004. Diagnoses of children were made according to DSM-IV criteria via the ADI-R and the ADOS. Patients with abnormal karyotypes and dysmorphic features were excluded. Most of the families were father-mother-child trios but some had more than one affected child. All affected biological siblings were assessed with the same diagnostic tools. We have ascertained and assessed 80 families (282 individuals) from Iran.

#### Finnish Autism Case-Control Study

The families for the Finnish autism dataset were recruited via Finnish university and central hospitals, mainly Helsinki University Hospital, Jyväskylä Central Hospital and Kuopio University Hospital. The control samples originate from Finnish population cohorts [15].

Two groups of families were included 1) families with at least one child diagnosed with autism, including families with additional siblings diagnosed with other ASDs or 2) families with no individuals diagnosed with strict autism but at least two individuals with a diagnosis of Asperger Syndrome. [16-19].

Diagnostic evaluations were made by a multidisciplinary group of clinicians at the neurological department of hospitals. Data were collected from extensive diagnostic examinations including neurological examinations, assessment of developmental history as well as psychological and neuropsychological examinations. Final diagnoses were based on ICD-10 and DSM-IV diagnostic nomenclatures. Families with known associated medical conditions or chromosomal abnormalities such as fragile X syndrome were excluded from the study. All families were Finnish except for one family where the father was of Turkish origin.

Subsequently, the ADI-R was administered to autism families willing to continue to participate in the study. In a subset of the Finnish autism families a 96% concordance rate was observed between ICD-10 and ADI-R diagnosis of autism [20] making the Finnish autism families clinically comparable to international family sets used for genetic studies.

One individual was included from each family in the case-control datasets, choosing the most severely affected individual (i.e. autism before Asperger Syndrome). If several individuals in the family had the same diagnosis, the individual with highest genotyping success rate was included. Genotyping was performed in multiple stages using Illumina HumanHap300/550, HumanHapCNV370 and Human610 arrays. All genotypes were called using Illumina BeadStudio.

#### Population-Based Autism Genetics and Environment Study

The Population-based Autism Genetics and Environment Study (PAGES) builds upon an epidemiological sample of subjects diagnosed with autistic disorder (AD; ICD-9 code 299A or ICD-10 codes F84.0–F84.1) from birth and medical registries in Sweden, as well as subjects from Sweden who were not diagnosed with any ASD. DNA from subjects were genotyped on the Illumina Human OmniExpress Exome BeadChip, of which the OmniExpress content of more than 715,000 single nucleotide polymorphisms (SNPs) were used for this study. Inclusion/exclusion criteria for PAGES samples and genetic analyses of PAGES data are given in [6].

Additional Ethics Approval Statement: This study has been reviewed and approved by institutional review boards at the Karolinska Institutet, Icahn School of Medicine at Mount Sinai and Carnegie Mellon University.

#### Simons Simplex Collection

DNA of individuals from Simons Simplex Collection (SSC) families were genotyped for a million or more single nucleotide polymorphisms (SNPs) on one of three array versions - Illumina 1Mv1, Illumina 1Mv3 Duo, or Illumina HumanOmni2.5M. Members of each family were analyzed on the same array version. All families were simplex [21], with only the proband being affected by ASD, as assessed by evaluation of family history. Probands, ages 4 to 18, were diagnosed using the ADI-R [8] and ADOS [9] as administered by expert clinicians. Genome-wide association analyses were previously reported elsewhere [7]. Approved researchers can obtain the SSC population data set described in this study by applying at https://base.sfari.org.

Additional Ethics Approval Statement: This study was overseen by the IRB at both Yale (HIC 0301024156) and UCSF (IRB: 14-14726 Ref: 146621). The data reported in this paper can be downloaded from SFARI Base (http://sfari.org/resources/sfari-base).

#### Weiss Laboratory Autism Collection

The Weiss Laboratory Autism Collections are a combination of Californian cohorts including the UCSF/Weiss (42 trios, 13 cases), UCSF/Hendren [22, 23] (27 cases), USC/Levitt [24] (44 cases, 32 controls), CHARGE/Hertz-Picciotto [25] (60 trios, 181 cases, 176 controls; http://beincharge.ucdavis.edu/), and the Autism Phenome Project (APP/Amaral; 84 cases, 54 controls; https://www.ucdmc.ucdavis.edu/mindinstitute/research/app/). Genotyping was performed at the UCSF genome core facilities by the LA Weiss laboratory for trios and case-control data from California using the Affymetrix Axiom EUR arrays [26]. Analysis was limited to European-Americans (as determined by principal components analysis).

UCSF/Weiss: ASD probands were recruited with a clinical diagnosis of an ASD. Individuals with known genetic cause (e.g. Rett syndrome, Fragile X) were excluded. UCSF/Hendren: A diagnosis of autism in all participants was confirmed by the Diagnostic and Statistical Manual-IV: TR criteria, as well as the ADOS conducted by a licensed psychologist trained to research reliability. If any diagnostic questions arose after the above were completed, the ADI-R was also conducted, followed by diagnostic agreement from a consensus raters’ meeting reviewing all available diagnostic information. All subjects were required to have a nonverbal IQ of 49 or above, confirmed by the Wechsler Preschool and Primary Scale of Intelligence, Mullen Scales of Early Learning, or the Wechsler Intelligence Scale for Children conducted by a licensed psychologist. USC/Levitt: To confirm ASD diagnoses, children (5-18 years old) in the ASD group were assessed with the ADOS. Exclusion criteria included severe sensory or motor impairment, neurodevelopmental disorders of known aetiology (e.g. Fragile X Syndrome), gestational age less than 36 or greater than 42 weeks, and birth weight less than 2500 grams. These individuals were also recruited based on positive/negative GI symptoms as described elsewhere [24, 27]. CHARGE/Hertz-Picciotto: Children were aged 2 to 5 years at enrolment, born in California, living with biological mother, parent and child speak English or Spanish, current residence in a 22-county region of California. Children were excluded if they displayed such severe disabilities that standardized instruments would provide invalid measures, e.g. blindness. All children who were recruited with a previous diagnosis of autism or ASD were evaluated using ADOS and ADI-R. Only confirmed cases, based on standard criteria [28] were classified as ASD. All control children who were recruited from the general population were administered the Mullen Scales of Early Learning (MSEL) and Vineland Adaptive Behaviour Scales (VABS) and SCQ. Those whose scores were higher than two standard deviations below the mean on one and higher than 1.5 SD's below mean on the other, and <12 on the SCQ were classified as controls. Any child who scored >12 on the SCQ was further evaluated using the ADOS and ADI-R. If they met criteria for ASD, they were classified as ASD. All tests were administered by trained clinicians who had attained research reliability on the instruments they conducted. APP: Inclusion criteria for the APP is a diagnosis of ASD through ADOS or ADI-R. Typically developing controls were screened and included after assessment with the Social Communication Questionnaire and the Mullen Scales of Early Learning (MSEL). Controls were excluded if they scored > 11 on the SCQ or revealed developmental scores within two standard deviations of the mean for performance quotient and verbal quotient subscales on the MSEL. Exclusion criteria for controls included a diagnosis of specific language impairment, or any known developmental, neurological, or behavioural problems. Further inclusion criteria for all children, both controls and children with ASD, included being native English speakers, ambulatory, and with no suspected vision or hearing problems. All diagnostic assessments were conducted or directly observed by trained, licensed clinical psychologists who specialize in ASD and had been trained per research standards for these tools.

Ethics Approval Statement: This study was approved by the relevant institutional review board [22-25].

### Replication Cohorts

Two replication cohorts were assembled. The first included individuals from the iPSYCH Initiative, a Danish population-based collection that includes linked treatment registers and DNA samples archived in blood spots. The iPSYCH autism sample contains an approximately equal number of cases as those genotyped in the PGC collection. A second replication sample includes a combination of three collections, two from deCODE; an Icelandic population-based collection and a third from the SEED Phase I collection. Although large in total sample, these collections contain a combined number of cases that comprise approximately 6.5% of the discovery case sample. The contributing Replication Cohorts are;

1. deCODE Autism Collection
2. iPSYCH Autism Collection
3. SEED Phase I Autism Collection

#### iPSYCH Project

Pre-publication consortium data; for questions email [mjdaly@atgu.mgh.harvard.edu](mailto:mjdaly@atgu.mgh.harvard.edu) and [anders@biomed.au.dk](mailto:anders@biomed.au.dk)

The iPSYCH-ASD collection is a population-based case-control ASD sample derived from the Danish Neonatal Screening Biobank hosted by Statens Serum Institut, comprising dried bloodspots (Guthrie cards) from all individuals born in Denmark since 1981. The samples can be linked to the Danish register system, including the Danish Psychiatric Central Register. DNA extracted from the bloodspots was successfully amplified and employed in GWAS as described previously [29, 30]. The iPSYCH-ASD project aims to genotype all Danish individuals with an available bloodspot and an ASD diagnosis in their medical record (ICD codes F84.0, F84.1, F84.5, F84.8 and F84.9). This study has been approved by the Danish research ethical committee system. This analysis employs data generated for the first ten genotyping waves of the iPSYCH-ASD collection, which contain 7,783 ASD cases and 11,359 controls. All individuals in the sample were born between 1981 and 2005. Genotyping was performed at the Broad Institute. For the iPSYCH sample, quality control and imputation was performed by the iPSYCH-Broad Autism Group. The data were cleaned and analyzed using the Ricopili pipeline at the Danish high performance computing cluster GenomeDK (<http://genome.au.dk>) at iSEQ, Centre for Integrative Sequencing, Aarhus University.

Additional Ethics Approval Statement: This study has been approved by the Danish research ethical committee system.

#### deCODE/SEED Phase I Collection

We combined GWAS via meta-analysis from the Icelandic deCODE collection (GWAS based on N = 483 cases, 136,203 controls); the non-Icelandic deCODE collection (incorporating individuals from Ukraine, Serbia and Georgia; GWAS based on N = 296 cases, 388 controls) and the Study to Explore Early Development (SEED) phase I collection (GWAS based on N= 590 cases, 717 controls [31]). In total this second replication set included data from GWAS of 1369 cases and 137,308 controls.

The Icelandic sample has been described previously [32]. Briefly, patients were ascertained through the State Diagnostic Counselling Center and the Department of Child and Adolescent Psychiatry, and were diagnosed based on ICD-10 criteria. Controls were recruited through ongoing projects at deCODE. Patients and controls from Georgia, Serbia and Ukraine were recruited by mental health clinics in their respective countries, with patients diagnosed using ICD-10 criteria [33]. Samples were genotyped using Illumina Whole genome genotyping platforms [33]. Following genotyping, individuals having evidence of ancestry outside their reported group (based on long-range phased haplotype analysis for the Icelandic samples, and on STRUCTURE and principal component analysis for the non-Icelandic samples) were excluded.

The Study to Explore Early Development (SEED) phase I is a national autism case-control study that has enrolled over 2,800 children and their parents across 6 sites in the US [31]. ASD-affected children between ages 2-5 were identified through clinic and education systems serving children with developmental problems. ASD was confirmed via the ADI-R [8] and ADOS [9]. Control children born in the same birth date window in the same zip codes were identified via vital records and were screened negative via the Social Communication Questionnaire [34] or seen in person to confirm no ASD or developmental problems. Genome-wide genotyping data for SEED samples was collected on either of two platforms: The Illumina 1M Quad BeadChip or a custom designed Affymetrix Axiom array. Ancestry was determined using the Eigensoft package [35]. Individuals greater than 4 standard deviations from the mean eigenvector values (for any of principal components 1-3), computed using the 1000 genome project European reference population, were excluded from our analyses.

## Quality Control, Imputation, Association and Meta-Analyses

### Ricopili Pipeline

Quality control, and imputation were performed by the PGC Statistical Analysis Group for each dataset separately and by the iPSYCH-Broad Autism Group for the iPSYCH sample. The core pipeline “Ricopili or Rapid Imputation Consortium Pipeline” has been used across the PGC initiative and enables robust, comparable generation of high quality imputed datasets from disparate genotype platforms. The pipeline is implemented on the Lisa Cluster, which is installed and maintained by SURFsara in the Netherlands. Additional details of the ricopili pipeline are available at <https://sites.google.com/a/broadinstitute.org/ricopili/home>.

#### Pre-Imputation Quality Control

To limit potential study or array biases, all quality control, imputation and association analyses were performed separately in batches defined per contributing site and array. As part of the PGC initiative it is not uncommon to identify participants enrolled in more than one contributing study (see [36]) . Prior to imputation duplicate individuals across contributing sites were removed. The quality control parameters for retaining SNPs and subjects were: SNP missingness < 0.05 (before sample removal); subject missingness < 0.02; autosomal heterozygosity deviation (| F_het_ | < 0.2); SNP missingness < 0.02 (after sample removal); difference in SNP missingness between cases and controls < 0.02; and SNP Hardy-Weinberg equilibrium (*P* > 10^−6^ in controls or *P* > 10^−10^ in cases). For family based cohorts (trios) we applied the following further QC steps: subject with > 2000 Mendel errors and SNPs with > 4 Mendel errors. Families with more than one offspring were divided into separate trios (same parents each) and treated as independent trios in downstream analyses.

#### Defining pseudo-controls

Several contributing sites have collected their participants using a parent-proband trio design. This model was historically favoured over the case-control design and uses an alternate family based association test, the transmission-disequilibrium-test, which is robust to the presence of population structure [37]. Advances in the identification and correction/ adjustment for population structure in the GWAS-era re-invigorated a favouritism for the case-control design. In the context of this study, we converted the parent-proband trios to proband-pseudocontrols using the --tucc flag in PLINK. The pseudocontrol to each affected offspring is created from the non-transmitted alleles from the parents and therefore effectively concordant for sex. These pseudocontrols are perfectly matched to each case. The principal advantage of the pseudocontrol approach is that it is compatible with our imputation and association pipelines. There is no power advantage over FBAT, indeed our approach is a simplification of FBAT chosen for computational efficiency. This simple data and analysis structure yields results essentially identical to FBAT for trios, which make up the great bulk of our data. Analysis of our design and approach can be found elsewhere [38, 39], although those manuscripts were focused on somewhat broader topics. A direct comparison of results and the fact that the results are similar is reported in Anney et al. 2010 [1], the first Autism Genome Project GWAS, in their methods section.

#### Genotype **Imputation**

Genotype imputation was performed using the pre-phasing/imputation stepwise approach implemented in IMPUTE2 / SHAPEIT (chunk size of 3 Mb and default parameters) [40, 41]. We applied the default effect population size of 20000. The imputation reference set consisted of all 2,184 phased haplotypes from the full 1000 Genomes Project dataset (Phase 1; August 2012, 30,069,288 variants, release “v3.macGT1”). Chromosome X imputation was conducted for subjects passing quality control for the autosomal analysis with the additional exclusions of chrX SNPs with missingness ≥ 0.05 or HWE p < 10^-6^ in females. Imputation was done using the proband-pseudocontrol genotypes.

After linkage disequilibrium pruning (r^2^ > 0.02) and frequency filtering (MAF > 0.05) of the intersection of SNPs, there were ~20,000 autosomal SNPs across all 14 cohorts. This SNP set was used for robust relatedness testing and population structure analysis. Relatedness testing was performed using PLINK v1.9 [42, 43]. For trio datasets, we removed only fully overlapping affected offspring ($\hat{\pi}$> 0.9) but kept sibling trios. For case control datasets, pairs of subjects with $\hat{\pi}$> 0.2 were identified and one member of each pair removed at random after preferentially retaining cases over controls and trio members over case-control members.

Principal components estimation was done with the same collection of autosomal SNPs. We tested the first 20 principal components for phenotype association (using logistic regression with study indicator variables included as covariates) and evaluated their impact on the genome-wide test statistics using λ [44]. Four principal components were included in all association analyses with case-control setup. For chrX, gene dosages in males were scored 0 or 2 to account for hemizygosity (dosages in females were scored 0/1/2 as for autosomal markers in both genders). For chrX we used sex as a covariate in association analysis.

#### Defining European Ancestry Subset

We defined a subset of each genotype set based on putative genetic ancestry. Individuals were classified as being of European ancestry based on similarity to the CEU-TSI HapMap reference populations. Individuals who were less than 10 standard deviations from the reference mean were considered as being of European ancestry (see Figure S17).

#### Logistic Regression, Meta-analyses and SNP filtering

We tested all 14 cohorts separately for association under an additive logistic regression model using PLINK. For samples derived from parent child trios, we applied a case-pseudocontrol design where the pseudocontrol was created with the non-transmitted alleles from the parents. Since these pseudocontrols are perfectly matched to each case, no covariates were used in association analysis from these trio cohorts. For the non-trio cohort, each regression included derived principal components as covariates [45]. SNPs with imputation quality (INFO Score) less than 0.6 were excluded from the meta-analyses. We performed meta-analyses of the 14 sets of results using an inverse-weighted fixed effects model [46] implemented in METAL (<http://csg.sph.umich.edu//abecasis/Metal/>) [47].

### GWAS Summary Data

Using data from the meta-analyses described above, we further excluded SNPs if imputation was unsuccessful in more than one subset and further limited reporting to common SNPs. For each dataset, chromosome and base-pair location based on hg19 build and reports are limited to markers with INFO score > 0.6 and minor allele frequency > 5%. The full data sets are available on the PGC data downloads website (<http://www.med.unc.edu/pgc/results-and-downloads>);

- daner_AUT_meta14_WW_all.hg19.Mar2016_info_0.60_maf_0.05.tsv.gz – Worldwide GWAS
- daner_AUT_meta14_EU_all.hg19.Mar2016_info_0.60_maf_0.05.tsv.gz – European GWAS
- daner_AUT_meta14_WW_noswm3.hg19.Mar2016_info_0.60_maf_0.05.tsv.gz – Worldwide GWAS minus Swedish PAGES GWAS

Manhattan and PP plots were rendered in STATA using self-authored programmes graphPP and graphManhattan. PP-plots for each cohort is shown in **Figure S1**. The corresponding Manhattan and PP-plots for WW and EU (all 14 datasets) are shown in Figure S2 and Figure S3 respectively.

### LD Clumped GWAS Reports and Summary Plots

*Linkage disequilibrium (LD) independent signals:* LD independent SNPs associated at P < 10^-4^ were defined using the clump flag in PLINK v1.9 [42, 43]. Clumping was used to link additional associated markers within a 0.5Mb window surrounding to the primary association. Markers were linked if they were also associated at P < .05 and had an estimated LD with the index SNP of r^2^ ≥ 0.2. Associated regions were defined for each index SNP as the location spanning all linked markers. All LD statistics were calculated using the 1000 genomes project phase 1 integrated reference haplotypes.

Summarised data are reported in;

- “Additional File 2 - Clumped GWAS ASD WW.xls” for Worldwide ASD GWAS
- “Additional File 3 – Clumped GWAS ASD EUR.xls” for European ASD GWAS

These worksheets contain LD clumped summary statistics from GWAS meta-analysis for the autism GWAS including all 14 datasets.

For each of the top ranked association signals a Locus plot was generated to describe the range of the association, the gene and annotation overlap and the expected linkage disequilibrium of adjacent markers. Forest plots were also generated to describe the distribution of the collective associations for the 14 contributing datasets. Locus plots and Forest plots were rendered in STATA using the self-authored programs graphLocus and the STATA program -metan- (see Figures S4).

Each region was annotated as intersecting known gene coordinates, previously reported genome-wide significant associations and Expression Quantitative Trait Loci (eQTL) signals. Gene co-ordinates were downloaded from the UCSC genome browser ftp site: <ftp://hgdownload.cse.ucsc.edu/goldenPath/hg19/database/refGene.txt.gz>. Previously reported GWAS associated regions were downloaded from the NHGRI GWAS catalogue (<http://www.ebi.ac.uk/gwas>); associations reported at P < 5 x10^-8^ were retained. eQTL annotations for human cortex as defined by the work of Myers and colleagues [48], and consensus eQTL from human lymphoblastoid cell lines were downloaded from the seeQTL database at <http://www.bios.unc.edu/research/genomic_software/seeQTL/data/> [49]. Annotations were limited to both cis- and trans-eQTL with a Q-value < .05. Annotation overlaps were assigned using the bedmap tools in the BEDOPS package available at <http://bedops.readthedocs.io/> [50].

#### Association Locus Plots

Each association locus plot contains a header plot showing SNP coverage, with each SNP noted by a single black vertical line. Each association signal is plotted on the -log(10) scale and each SNP is represented by a single coloured marker; the index association is plotted as a single purple diamond, with additional markers coloured according to linkage disequilibrium (r2) with the index marker (see legend). A dark blue trace of the average recombination rate across the region is also plotted. Gene (and exons) and eQTL loci are plotted in the panel beneath the association data. eQTLs are represented by coloured “lollipops”; triangles and diamond markers represent cis-and trans-eQTLs respectively; blue and red markers represent LCL [44] and CNS [43] derived eQTLs respectively.

### Estimate of SNP-based Heritability

We sought to investigate the estimated heritability attributable to common additive genetic variation. We utilised the LD-score regression methodology applied to our summary GWAS statistics [51]. Analysis routines were implemented using the LD score regression python script ldsc.py (available at <https://github.com/bulik/ldsc>). Summary GWAS statistics were converted to *. sumstats format by applying the munge_sumstats.py script. Analyses were limited to markers with a minimum imputation quality info score of 0.8 and a minimum minor allele frequency of 0.05. According to the authors’ recommendations we further restricted our analyses to markers overlapping high-confidence HapMap3 SNPs (listed in <https://data.broadinstitute.org/alkesgroup/LDSCORE/w_hm3.snplist.bz2>). LD-scores were calculated locally using the 1000 genomes genotypes as a reference. Genetic correlation between the ASD noSWM3 datasets and the schizophrenia and rheumatoid arthritis (control) GWAS were also calculated using the ldsc.py script using the --rg flag. The rheumatoid arthritis GWAS was originally reported in the meta-analysis by Stahl and colleagues [52] available at: <http://www.broadinstitute.org/ftp/pub/rheumatoid_arthritis/Stahl_etal_2010NG/>. Additional graphical representation of these data is given in **Figure S10**.

### Gene-based Analyses (VEGAS2)

Based on the observation that up to 1/3 of associated loci harbour multiple independent effects contributing to a GWAS signal, [53-55] we performed gene-based tests which, under a heterogeneity scenario, will have more power than single SNP tests [53]. Gene-based statistics for the WW GWAS were calculated using VEGAS2 (<https://vegas2.qimrberghofer.edu.au/>) [56], a modification on the versatile gene-based association study (VEGAS) method. Analysis was limited to the top 10% of SNPs per gene, an approach which has previously been shown to give rise to higher sensitivity and lower false positive rates compared to other gene-set methods [57]. Manhattan and PP plots are for the VEGAS2 outputs are shown in Figure S11 and Figure S12. The full gene-based report is provided in;

- “Additional File 4 - VEGAS2 GWAS ASD WW.xls”

### Gene-set Analyses (INRICH)

Our primary hypothesis is that gene-sets previously implicated in ASD and neurodevelopment will be enriched in these GWAS. Therefore, we created sets based on lists of genes reported in the literature. INRICH supplementary files used in these analyses are;

- Additional File 5 - Candidate.sets
- Additional File 6 - Canonical.sets
- Additional File 7 - hg19_refgene_inrich.genes
- Additional File 8 - PGCSchizophrenia2014.sets

#### Candidate-Gene-Sets

We utilised gene lists derived from studies of ASD which utilised other technologies and variation types such as private, rare and de novo sequence and structural variation. From reports examining de novo/rare SNVs, gene sets can be broadly characterised as being related to the “TGF-β pathway”, “synaptic transmission”, “transcriptional regulation” and “neurodegeneration” [58], and from de novo/rare CNV studies in ASD, gene sets have been found to be related to “neuronal development and axon guidance”, “MAPK signalling” and “chromatin remodelling” [11]. We included genes implicated in ASD from extensive next generation sequencing efforts within the Autism Sequencing Consortium [59] and subsequent extended networks of genes derived from gene-targets, namely FMRP targets [60], CHD8-related genes [61], genes implicated as chromatin modifiers or those influencing post-synaptic density [62], brain-expressed genes under purifying selection which have been shown to be enriched in ASD [63], and cortical co-expression networks implicated in ASD (specifically early transcriptional regulation and synaptic development; M2, M3, M13, M16 and M17 from [64]). Gene-sets were also examined which include genes implicated in the recent PGC GWAS of schizophrenia [65], as well as expert annotated lists of genes implicated in intellectual disability and ASD [11]. Finally, we explored three genome conservation sets under the hypothesis that variation in conserved genes are likely to have greater impact than in those more tolerant to variation; firstly, a set of “constrained” genes identified by Samocha et al., [66] which contain a set of genes with significantly reduced functional coding variation in typically developing individuals. The remaining gene-sets further examine evolutionary related elements by exploring Human (HAR) and Primate Accelerated Regions (PAR). These are highly conserved genomic segments that have been shown to exhibit “accelerated” substitutions in the human and primate genomes respectively [67]. Moreover, PARs have been shown to be enriched in schizophrenia [68]. For the hypothesis driven model, gene-sets were restricted to those expressed in the brain as reported by Iossifov and colleagues [62]. The corresponding INRICH formatted *.set file for the candidate gene analyses is provided in <Candidate.set> and <PGCSchizophrenia2014.set>. A summary of the origin of these gene-sets are given in Table S1.

Table S1: Origin of Candidate Gene Sets.

| **Candidate Gene Set** | **Source in Ref.** | **Ref.** |
| --- | --- | --- |
| Chromatin Modifiers | Supplementary Table S7 | [62] |
| De Novo LGD In Schizophrenia | Supplementary Table S7 | [62] |
| Embryonic | Supplementary Table S7 | [62] |
| Essential Genes | Supplementary Table S7 | [62] |
| FMRP Targets | Supplementary Table S7 | [62] |
| Mendelian Disease Genes | Supplementary Table S7 | [62] |
| Post Synaptic Density | Supplementary Table S7 | [62] |
| ASD Risk Genes | Table 1 | [58] |
| Neurodegeneration Cluster | Figure 2 | [58] |
| Cell Communication and Synaptic Transmission Cluster | Figure 2 | [58] |
| Cell junction TGFβ pathway Cluster | Figure 2 | [58] |
| Transcriptional regulation Cluster | Figure 2 | [58] |
| ASD Risk Genes | Supplementary Table S6A | [11] |
| ASD High Risk Genes | Supplementary Table S16 | [11] |
| Chromatin Modifiers | Supplementary Table S15 | [11] |
| DAPPLE derived Gene list | Supplementary Table S15 | [11] |
| FMRP Targets | Supplementary Table S10 | [11] |
| Intellectual Disability Risk Genes | Supplementary Table S6B | [11] |
| MAP Kinase Genes | Supplementary Table S15 | [11] |
| Neuronal Genes | Supplementary Table S15 | [11] |
| ASD CNV Loci | Table 2 | [59] |
| ASD Risk Genes | Supplementary Table S6 | [59] |
| TADA Derived Genes | Supplementary Table S6 | [59] |
| FMRP Targets | Supplementary Table S2 | [60] |
| CHD8 Binding Targets | Supplementary Table S1 | [61] |
| M1 Module | Supplementary Table S1 | [61] |
| M2 Module | Supplementary Table S1 | [61] |
| M3 Module | Supplementary Table S1 | [61] |
| M4 Module | Supplementary Table S1 | [61] |
| Constrained Genes | Supplementary Table S2 | [66] |
| Human Accelerated Regions | Supplementary File 2xHARs.bed | [67] |
| Primate Accelerated Regions | Supplementary File 2xPARs.bed | [67] |
| Brain Critical Genes | Supplementary Table S7 | [63] |
| M2 Module | Supplementary Table S1A | [64] |
| M3 Module | Supplementary Table S1A | [64] |
| M13 Module | Supplementary Table S1A | [64] |
| M16 Module | Supplementary Table S1A | [64] |
| M17 Module | Supplementary Table S1A | [64] |

#### Canonical-Gene-Sets

In addition, we performed analyses against canonical gene-sets from the Gene-Ontology (GO) database. Briefly, genes to GO-term mapping was sourced from the NCBI ftp repository (<ftp://ftp.ncbi.nlm.nih.gov/gene/DATA/gene2go.gz>) and limited to human relationships (tax_id = 9606). GO-terms hierarchy was sourced (<ftp://ftp.geneontology.org/pub/go/ontology/go-basic.obo>) and after excluding obsolete GO-terms, the parent GO-terms were populated with daughter GO-terms to capture the complete gene-set composition for each term. We also included gene-sets posted as part of version 5.0 of the Molecular Signatures Database (MSigDB; [69]). The MSigDB gene sets are divided into a number of major collections. We incorporated the canonical gene-sets from the Reactome, National Cancer Institute (NCI), Kyoto Encyclopedia of Genes and Genomes (KEGG) and BioCarta databases. The corresponding INRICH formatted *.set file for the candidate gene analyses is provided in <Canonical.set>.

#### Defining Gene Coordinates

Gene coordinates for analyses were based on hg19 and to capture additional regulatory elements we incorporated an additional 35kb 5’ and 10kb 3’ of the transcription start and end coordinates respectively. Gene coordinates were sourced from the refGene database and analyses were limited to protein coding transcripts. Analyses such as INRICH require each GeneID to be assigned to a single set of coordinates; GeneIDs with multiple transcript coordinates were merged to include the largest overlapping transcripts. If multiple transcripts were present on more than 1 chromosome or non-overlapping and greater that 50kb 5’ or 3’ of the overlapping transcript they were considered *trans* or *duplications* and dropped from the analyses. The corresponding INRICH formatted *.genes gene co-ordinate file for these analyses is provided in <hg19_refgene_inrich.genes>.

#### Interval Enrichment Analyses (INRICH)

LD-Independent genomic intervals were described using the clump routine in PLINK. LD independent SNPs associated at P < .05 were defined using the clump flag in PLINK v1.9 [42, 43]. Clumping was used to link additional associated markers within a 0.5Mb window surrounding to the primary association. Markers were linked if they were also associated of P < .05 and had an estimated LD with the index SNP of greater than r^2^ of 0.2. INRICH analysis was performed in interval mode examining the top 1000 intervals ranked by index association p-value for the candidate analyses and the top 100 intervals for the canonical analyses. We chose a broader set of intervals for the candidate set analyses, allowing for a greater signal to noise in this hypothesis testing analyses. For the canonical gene-sets we used a more stringent set of intervals to reduce the putative signal to noise ratio in this discovery analyses.

Prior to clumping we excluded the HLA region (chr6;25000000-35000000). The interval mode describes an enrichment statistic E for each gene-set that is the number of intervals that overlap at least one target gene in the gene-set. The significance of E is approximated using permutation and an empirical p-value is generated from a null set of intervals which match the interval size, overlapping gene and SNP number to the original input intervals. To reduce computational burden gene-sets were filtered to a subset with a minimum and maximum gene content of 5 and 1000 respectively. Reporting was limited to gene-sets with an overlap with at least 3 associated intervals.

The Candidate.set gene-sets were applied to the full WW intervals, whereas due to overlap with the PGC Schizophrenia dataset, the PGCSchizophrenia2014.set was applied to the dataset derived from the WW minus the Swedish PAGES meta-analyses. Complete summary outputs from the INRICH analyses are reported in separate worksheets in;

- “Additional File 9 – INRICH GWAS ASD WW - Candidate.xls”
- “Additional File 10 – INRICH GWAS ASD WW noSWM3 - Schizophrenia.xls”
- “Additional File 11 – INRICH GWAS ASD WW - Canonical.xls”

### Partitioned Heritability

Partitioned heritability estimates were generated from summary GWAS statistics. As with SNP-based heritability estimates, GWAS summary statistics were converted to *. sumstats format by applying the munge_sumstats.py script. Analyses were limited to markers with a minimum imputation quality info score of 0.8 and a minimum minor allele frequency of 0.05. According to the authors’ recommendations, we further restricted our analyses to markers overlapping high-quality HapMap3 SNPs (listed in <https://data.broadinstitute.org/alkesgroup/LDSCORE/w_hm3.snplist.bz2>). Moreover, we restricted all analyses to annotations which cover greater that 1% of the SNPs included in the model. *Functional Annotations:* Functional annotations were parsed from the baseline model data and include annotations based on gene structure (promoter, intron, coding, UTR) and regulatory elements (Histone and DNAS-I hypersensitivity sites). The baseline model can be accessed at <https://data.broadinstitute.org/alkesgroup/LDSCORE/baseline_ldscores.tgz>. *Cell-Type-Specific Annotation:* 220 individual cell-type-specific annotations described in [51] and available for download at: <https://data.broadinstitute.org/alkesgroup/LDSCORE/cell_type_ldscores.tgz>. These annotations contain histone marks related to cell-specific histone binding sites, specifically the annotations relate to H3K4me1, H3K4me3, H3K9ac andH3K27ac. As with the functional annotation, these annotations were limited to those which cover greater than 1% of the SNPs included in the model (181 out of 220 cell retained). Candidate *Annotation:* We included annotations based on the candidate gene-sets described previously for INRICH analyses and with those canonical gene-sets which were found to be enriched in the INRICH analyses, at P ≤ .05. As noted above, annotations were based on gene co-ordinates, incorporating an additional 35kb 5’ and 10kb 3’ of the transcription start and end co-ordinates respectively. As with the INRICH analyses due to overlap with the PGC Schizophrenia dataset, the PGC Schizophrenia 2014 annotations were assessed in the WW minus the Swedish PAGES meta-analyses data only. Complete summary outputs from the Partitioned Heritability Analyses are reported in the worksheets;

- “Additional File 12 – LDSC GWAS ASD WW.xls “

### Replication analyses

We assessed the replicability of the top linkage independent loci observed with the combined discovery worldwide analyses (comprised of the 14 cohorts) against two replication collections; (1) the Danish iPSYCH data, and (2) the combined deCODE/SEED data.

#### Binomial Sign Test

The binomial sign test estimates the likelihood to observe a number of successful trials given the expected success rate and the number of trials. We used this to assess the likelihood to observe replication at P < .05 (expected proportion = .05) and to examine the likelihood to observe concordance of the direction of the effect of any two association studies given the expected proportions to be 0.5.

For the direction of effect analyses we included a number of additional steps. First, to avoid problems associated with extensive complex linkage disequilibrium, we excluded markers within the HLA region of chromosome 6. Second, concordance was classified as “not determined” if the effect in the replication was neutral (Beta = 0; OR = 1.0). Finally, we estimated the likelihood at each rank point, stepping through the data according to ranked discovery P-values, and calculated the likelihood of the observed concordance given all the SNPs at that rank and below. The likelihoods and concordance are subsequently plotted to determine the distribution of concordance across the dataset. See Figure 1 (main test) for concordance plots.

#### Meta-analyses

We also performed meta-analyses of the top linkage disequilibrium independent association findings for the combined discovery worldwide analyses (comprised of the 14 cohorts) against (SET 1) the iPSYCH data, (SET 2) the combined deCODE/SEED data and (SET 3) against both iPSYCH + deCODE/SEED combined. All analyses were performed using an inverse-weighted fixed effects model [46] implemented in METAL (<http://csg.sph.umich.edu//abecasis/Metal/>) [47]. A detailed summary of the replication results is reported in;

- “Additional File 13 – GWAS ASD WW - Replications.xls”

#### Comparison of PGC ASD GWAS and PGC Schizophrenia signal at 10q24.32

Both ASD and schizophrenia report GWS association at 10q24.32. In order to examine the overlap of these signals we have plotted and overlaid the association signals from the ASD and schizophrenia study across this region (see Figure S13). As can be seen from this figure the ASD signal is distinct from that observed in schizophrenia. Our signal does not extend into the schizophrenia GWAS peak, nor do we report any independent significant association in this peak.

### Cross Disorder Analysis

To further identify novel loci implicated in neurodevelopment, we examined the cross-disorder binomial sign test, followed by meta-analysis of the PGC Schizophrenia and PGC Autism datasets. The complete QC’d final reports for the ASD (worldwide) minus SWM3 data, PGC Schizophrenia meta-analysis are reported in daner_AUT_meta14_WW_all_SCZ_all.hg19.Mar2016_info_0.60_maf_0.05.tsv.gz and available on the PGC downloads page. The distribution of the binomial sign test for ASD versus schizophrenia and the putatively unrelated disorder rheumatoid arthritis are shown in Figure S14.

Manhattan plots from the schizophrenia by ASD meta-analysis were rendered in STATA using self-authored programmes graphManhattan (see **Figure S15**).

Abridged LD clumped GWAS reports of these data are reported in;

- “Additional File 14 – Clumped GWAS ASD + SCZ WW.xls”

After removing SNPs which were shown to be GWS in the PGC Schizophrenia analyses as well as GWS regions captured by these GWS loci we identified 10 putatively novel GWS neurodevelopmental loci. These data are reported in;

- “Additional File 15 – Clumped GWAS ASD + SCZ WW - Novel.xls”

Locus plots of these novel index SNPs are collated in Figure S16.

## Overview of Analytic PLAN

A schematic of the analytic plan used in this manuscript is described in **Figure S18**. This highlights the range of analyses and is a visual reference to where none primary datasets, specifically the ASD (worldwide) minus SWM3 data, are used instead of the primary ASD (worldwide) full data.

# Supplementary Figures

**Figure S1:** PP Plot for GWAS of Each Contributing GWAS of Worldwide Ancestries Autism Spectrum Disorder.


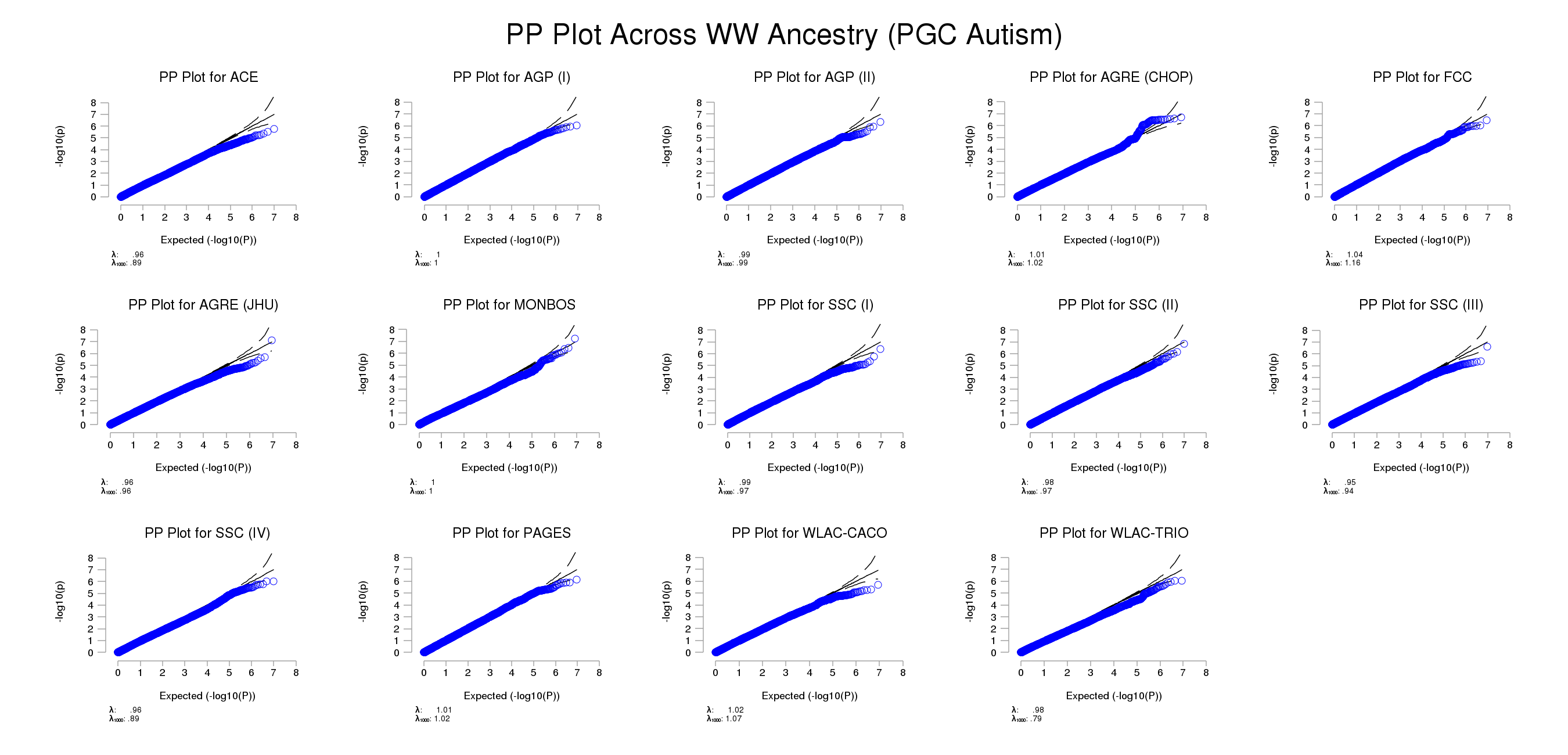


**Figure S2:** Combined PP and Manhattan Plot for GWAS of All (Worldwide) Ancestries Autism Spectrum Disorder


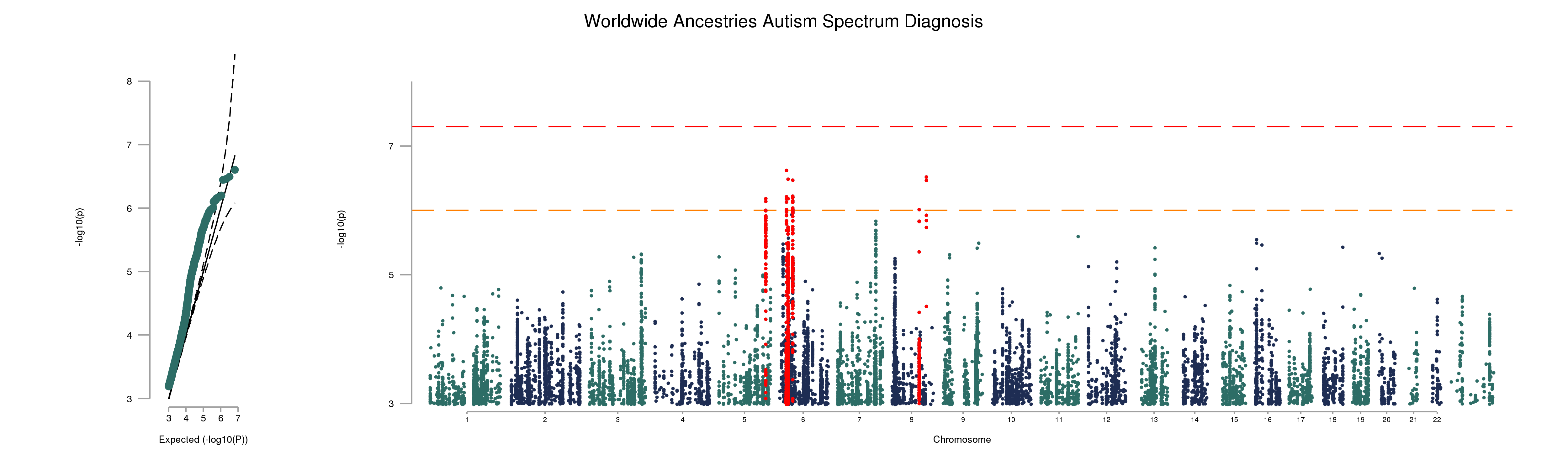


**Figure S3:** Combined PP and Manhattan Plot for GWAS of European Ancestries Autism Spectrum Disorder


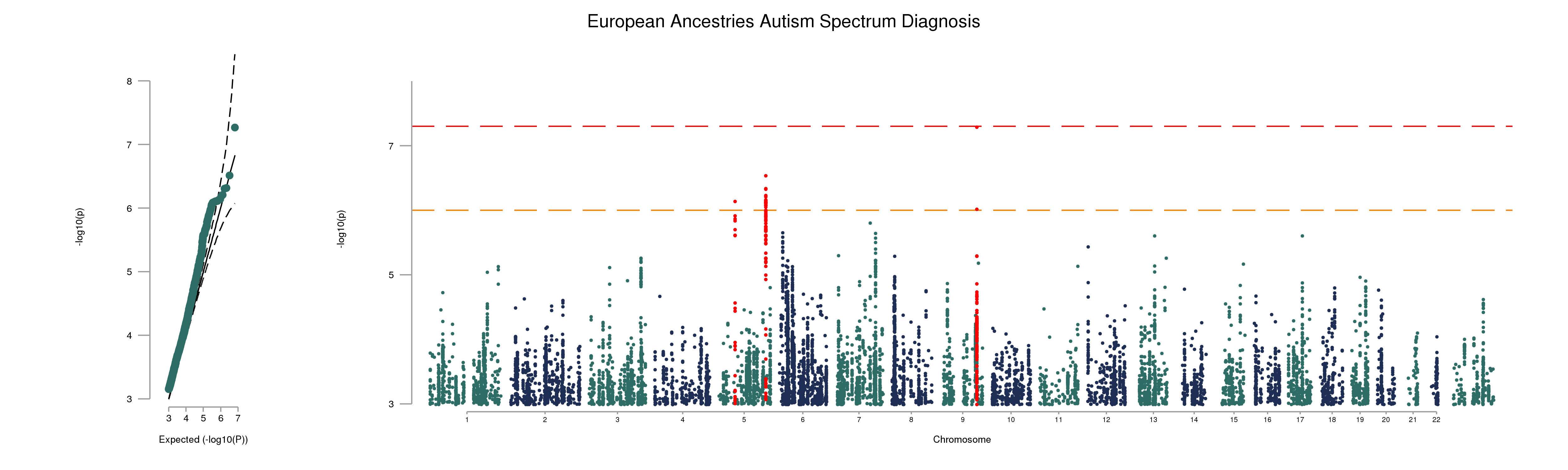


**Figure S4:** Forest plot for the top independent GWAS signals from the All (Worldwide) Ancestries Autism Spectrum Disorder analysis. Each Forest plot describes the study level association signal with the effect size shown as a black filled marker and 95% confidence intervals indicated by a black horizontal line. A filled grey box represents the weight of each study (based on sample size). Overall association findings are represented by an open diamond.


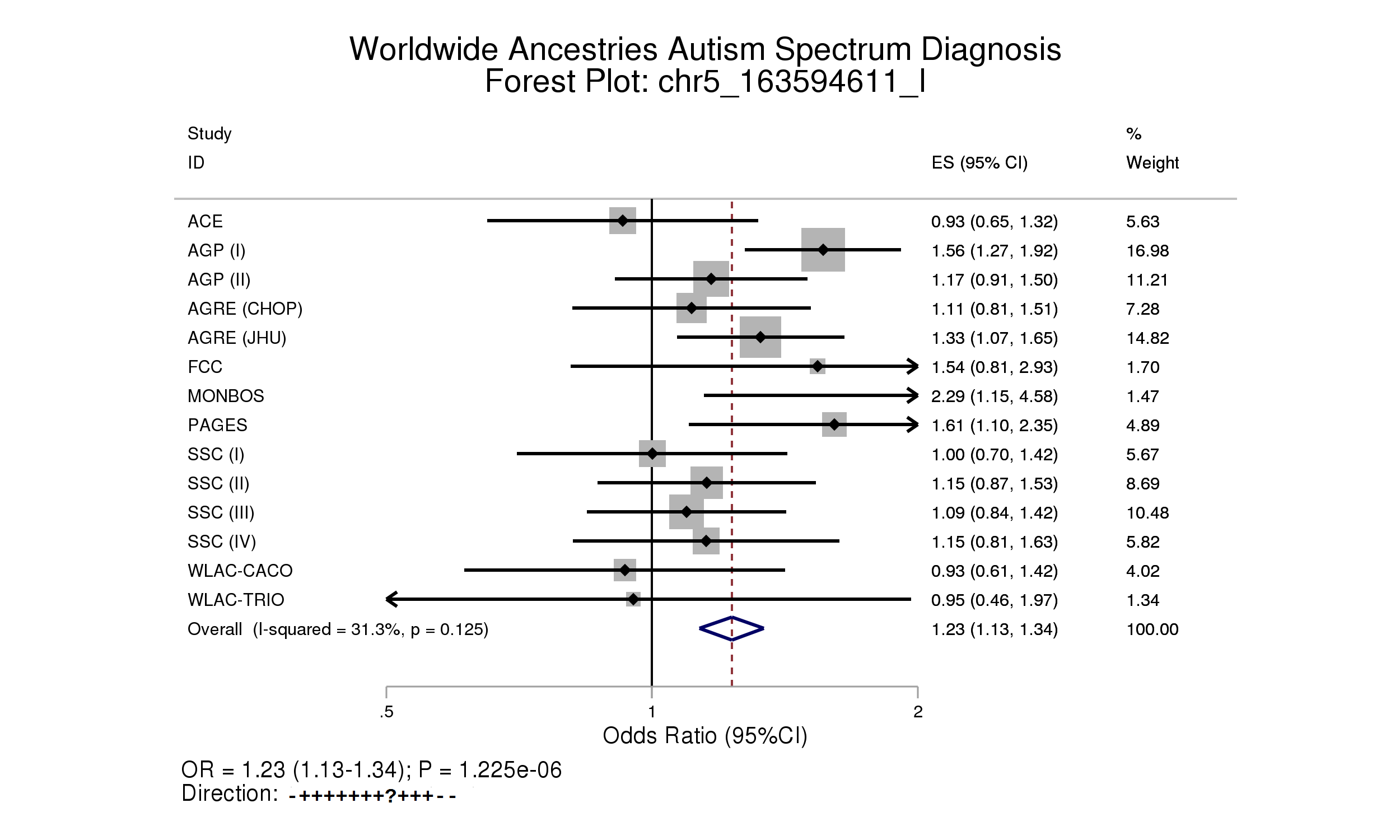

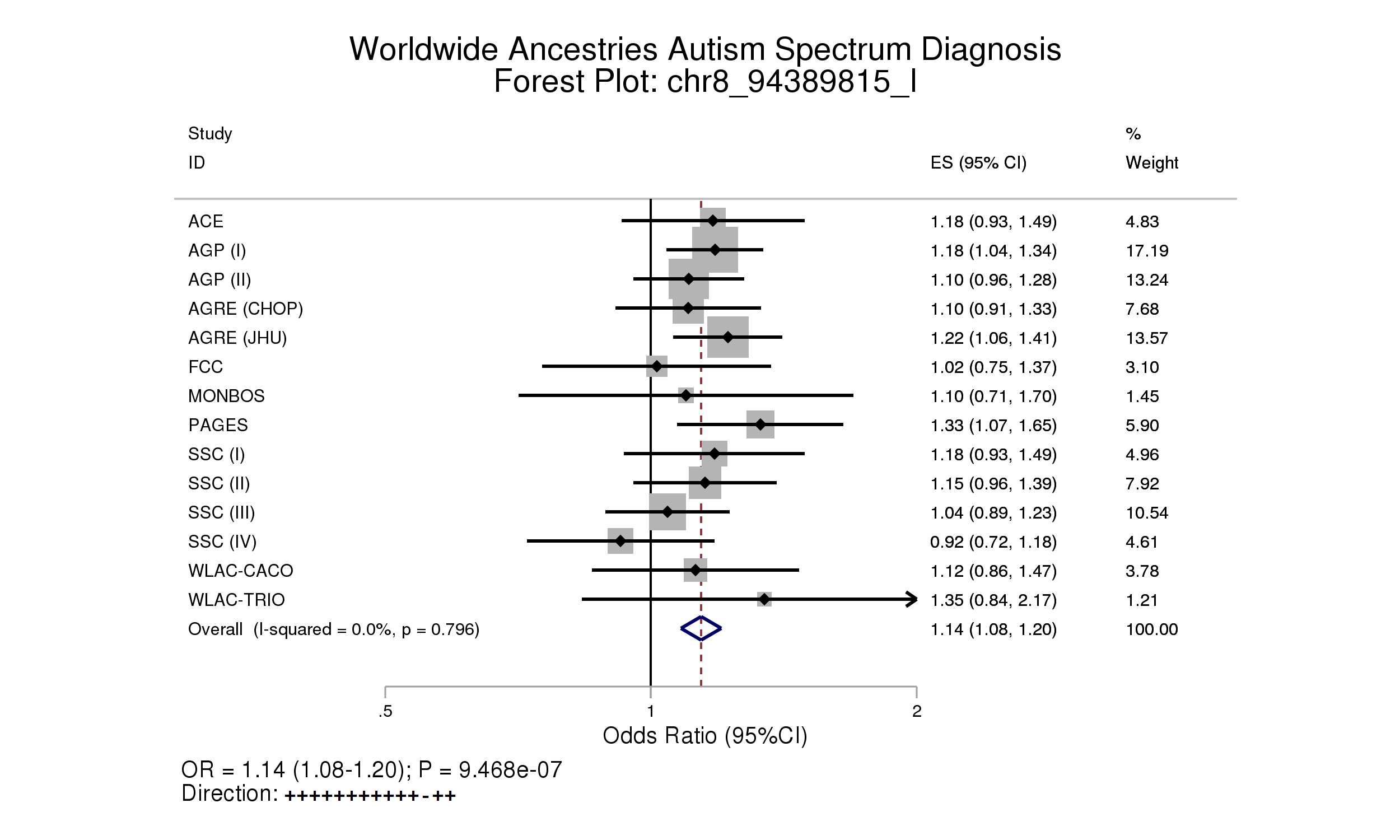

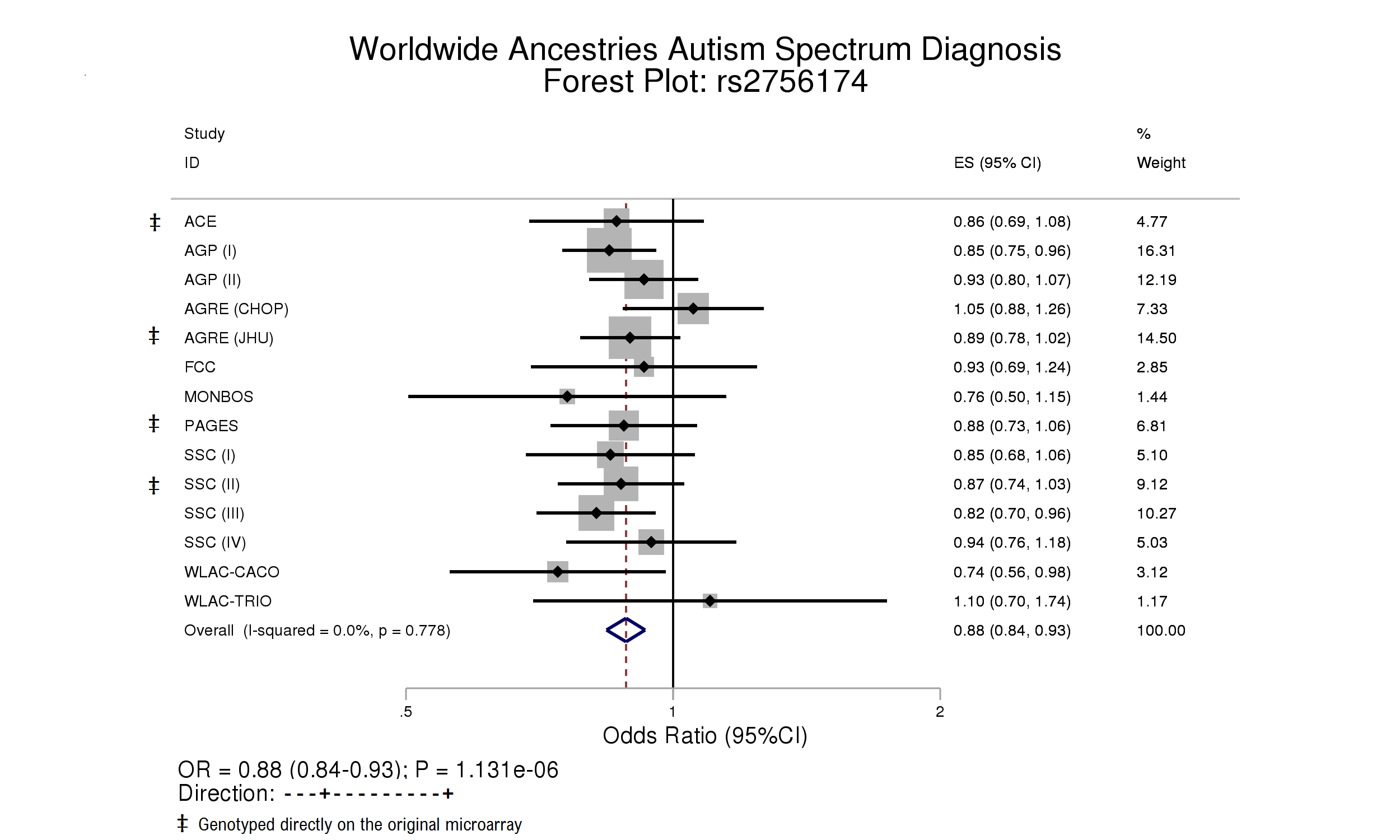

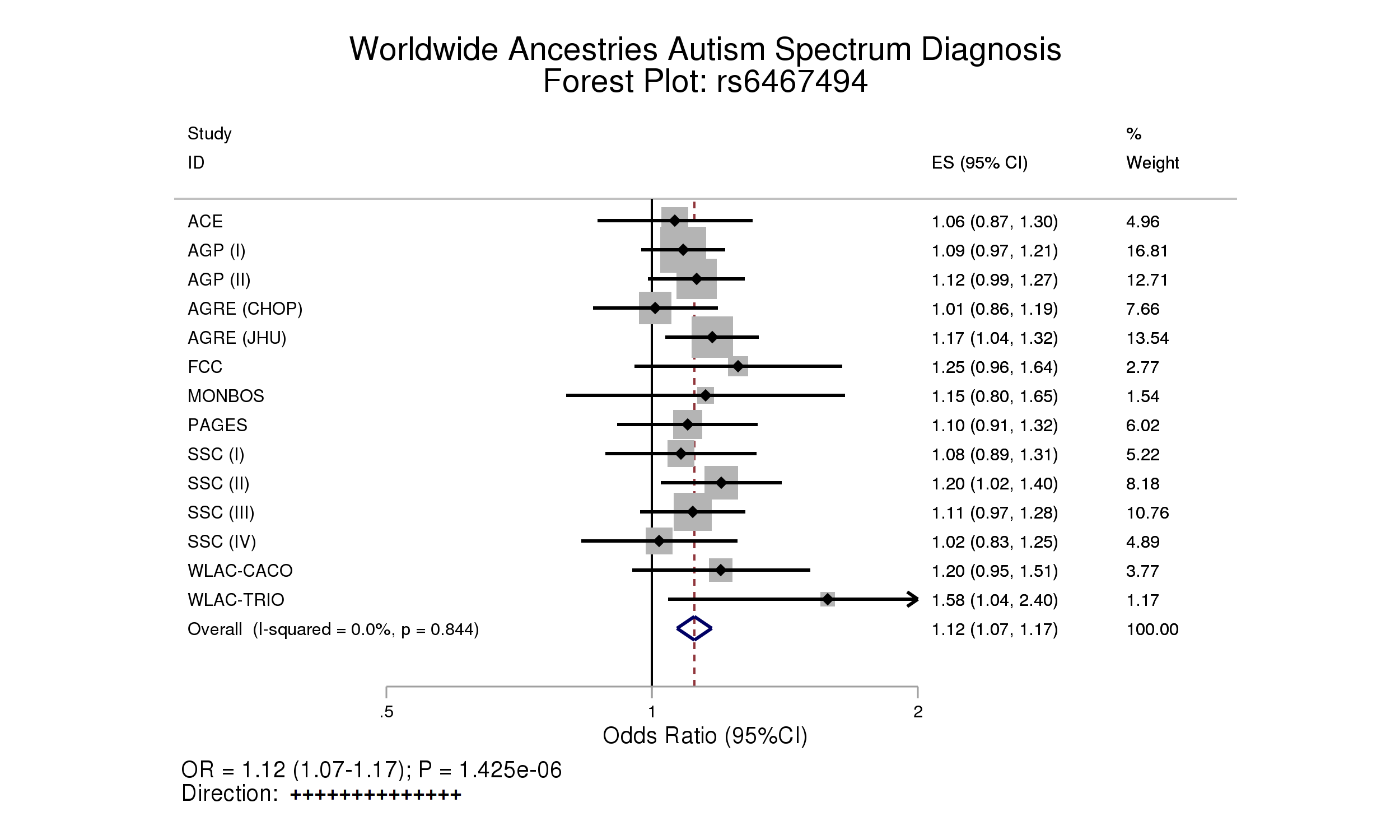

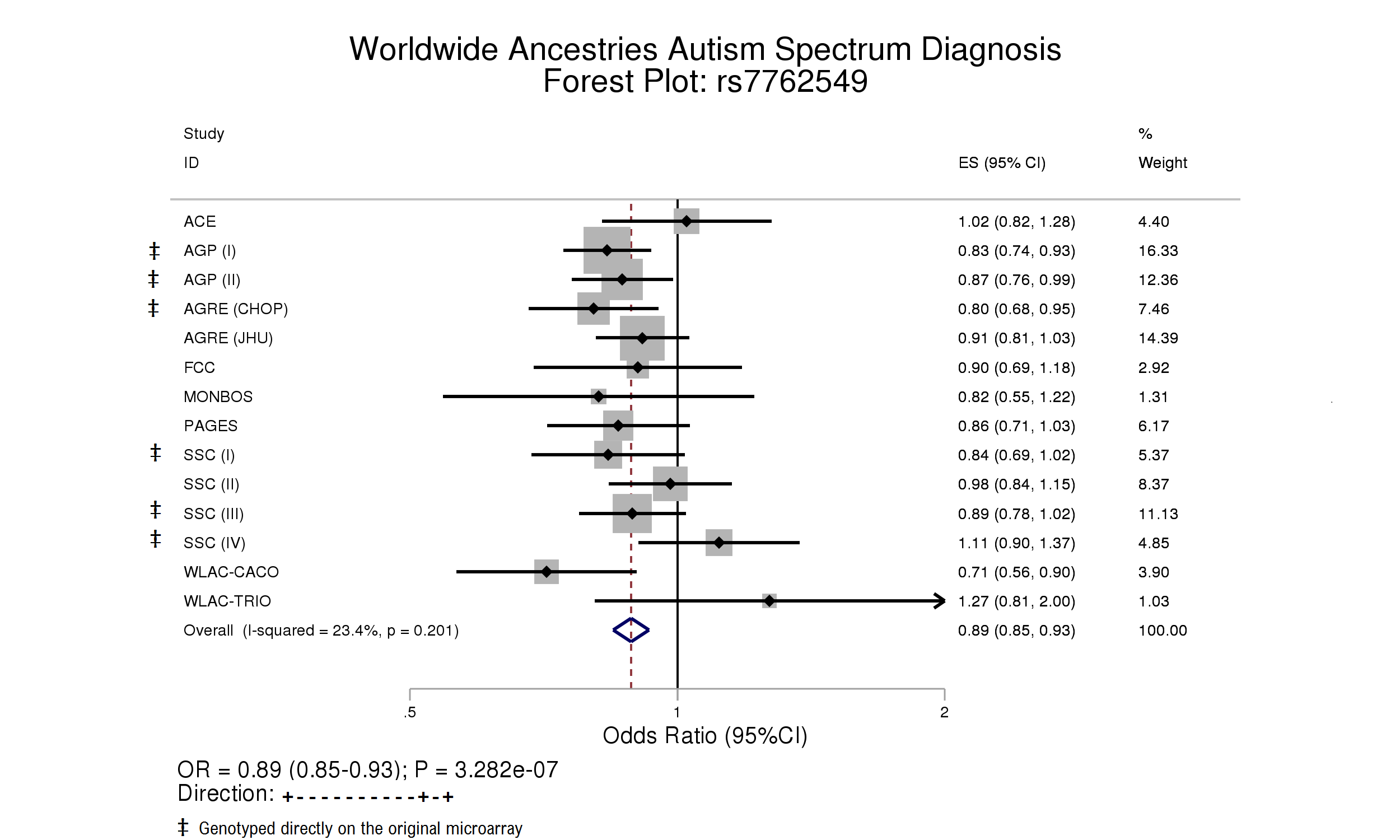

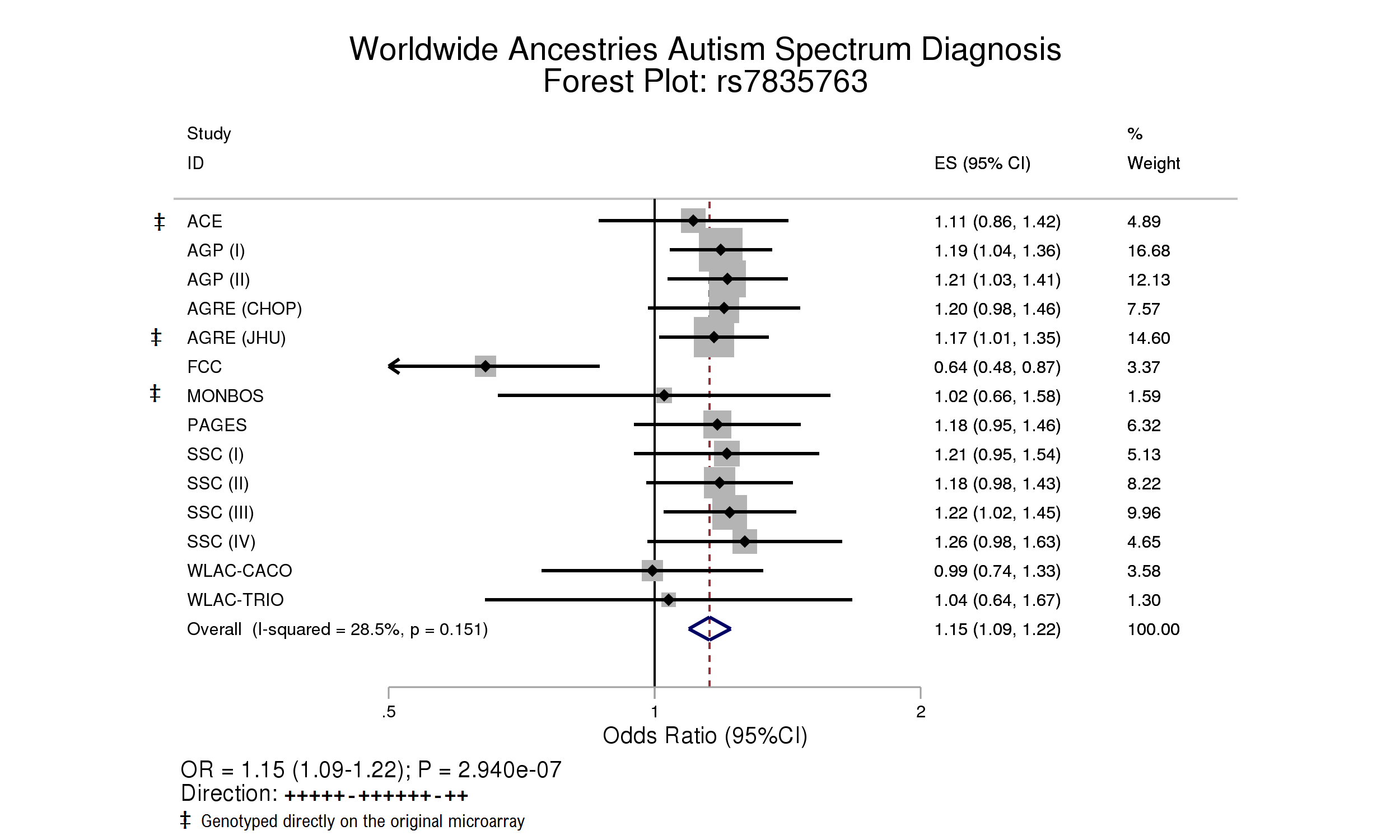

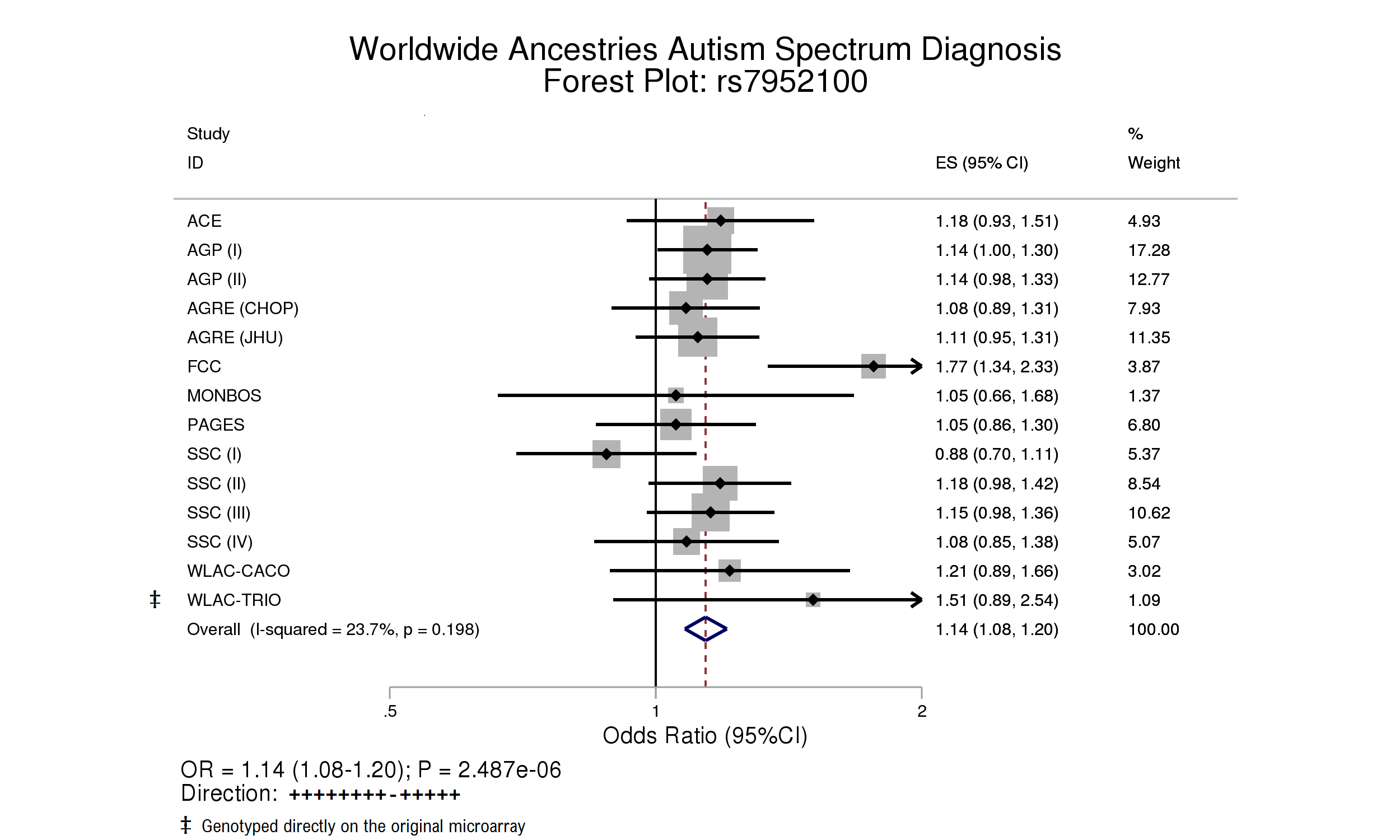

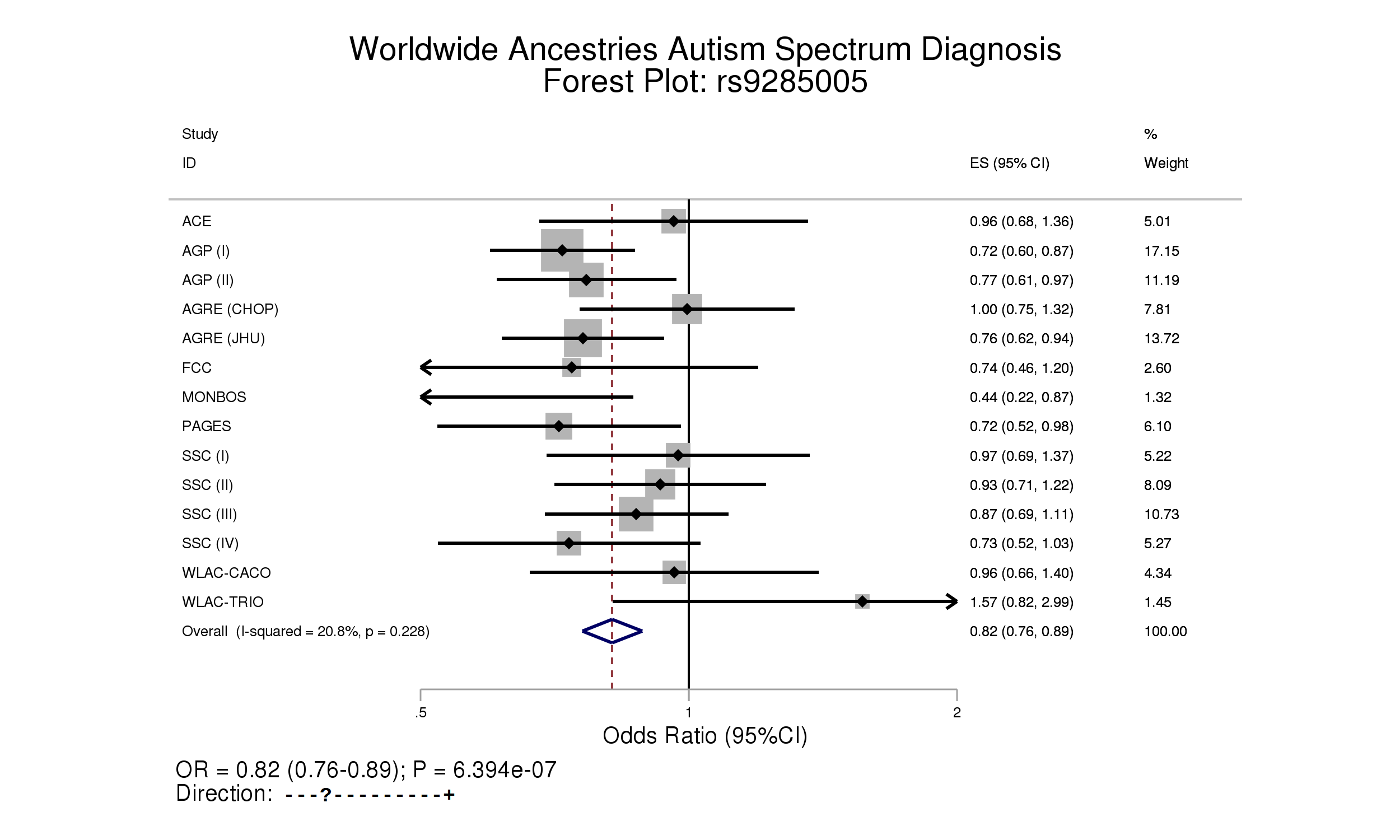

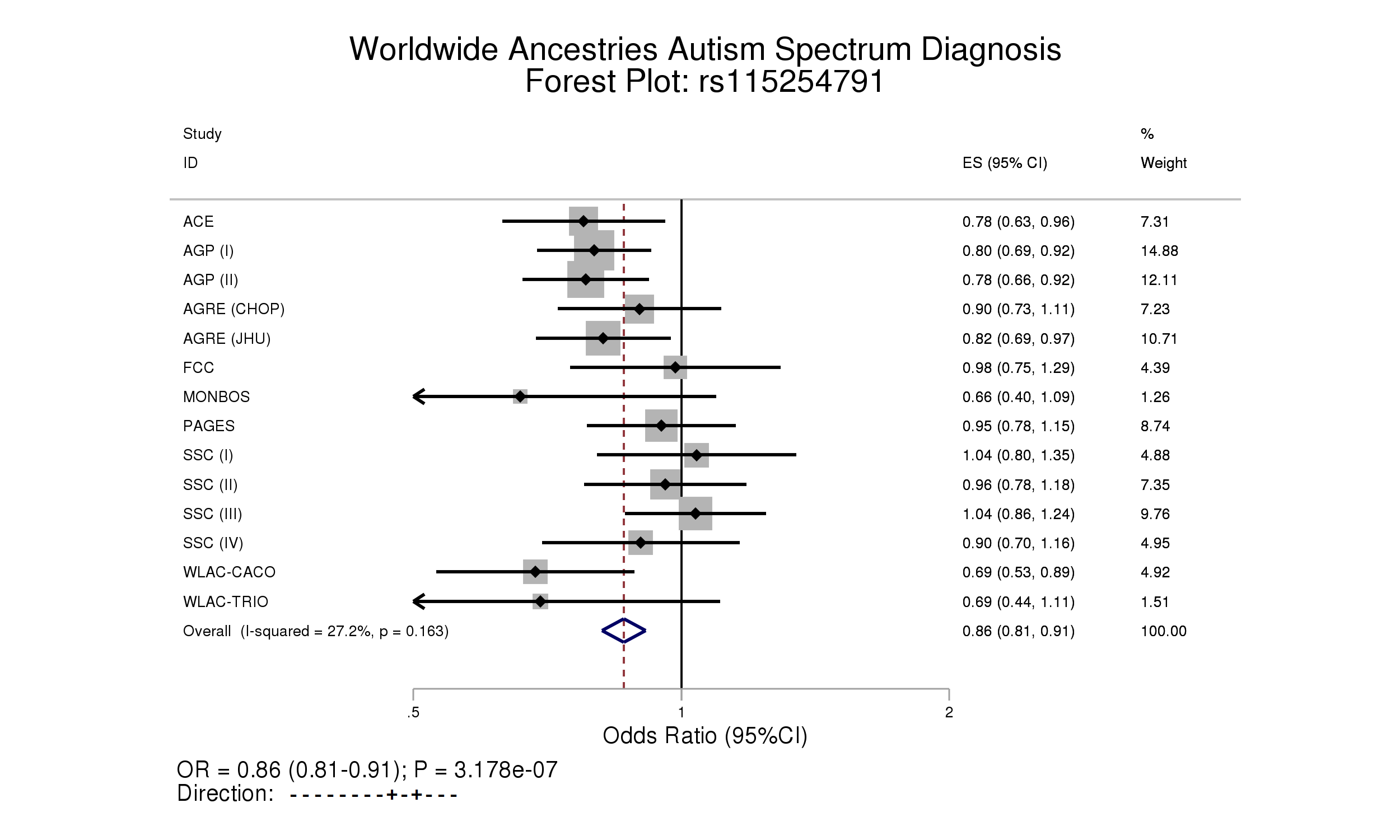

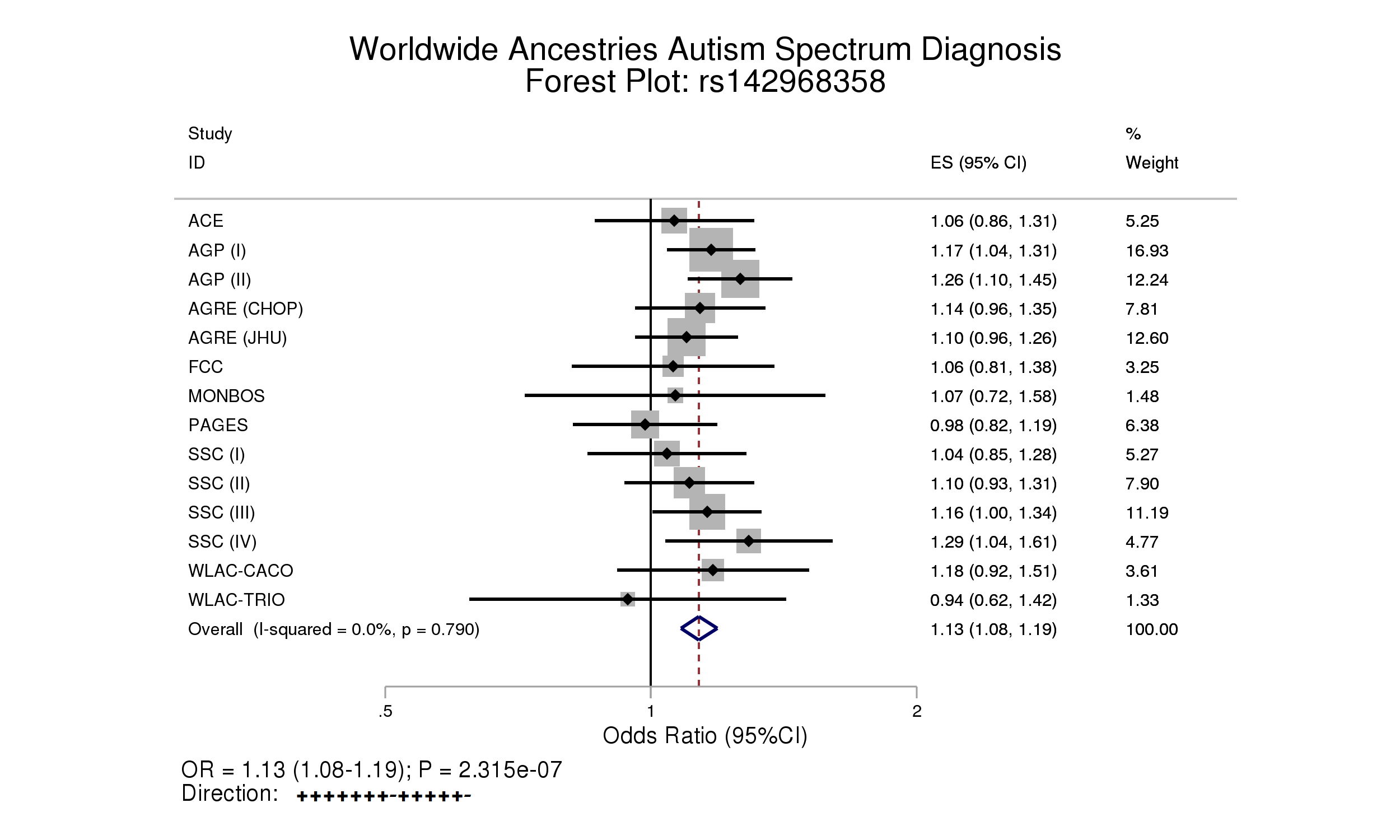


**Figure S5:** Association Locus plot for the index SNP rs142968358 in the Worldwide Autism GWAS.


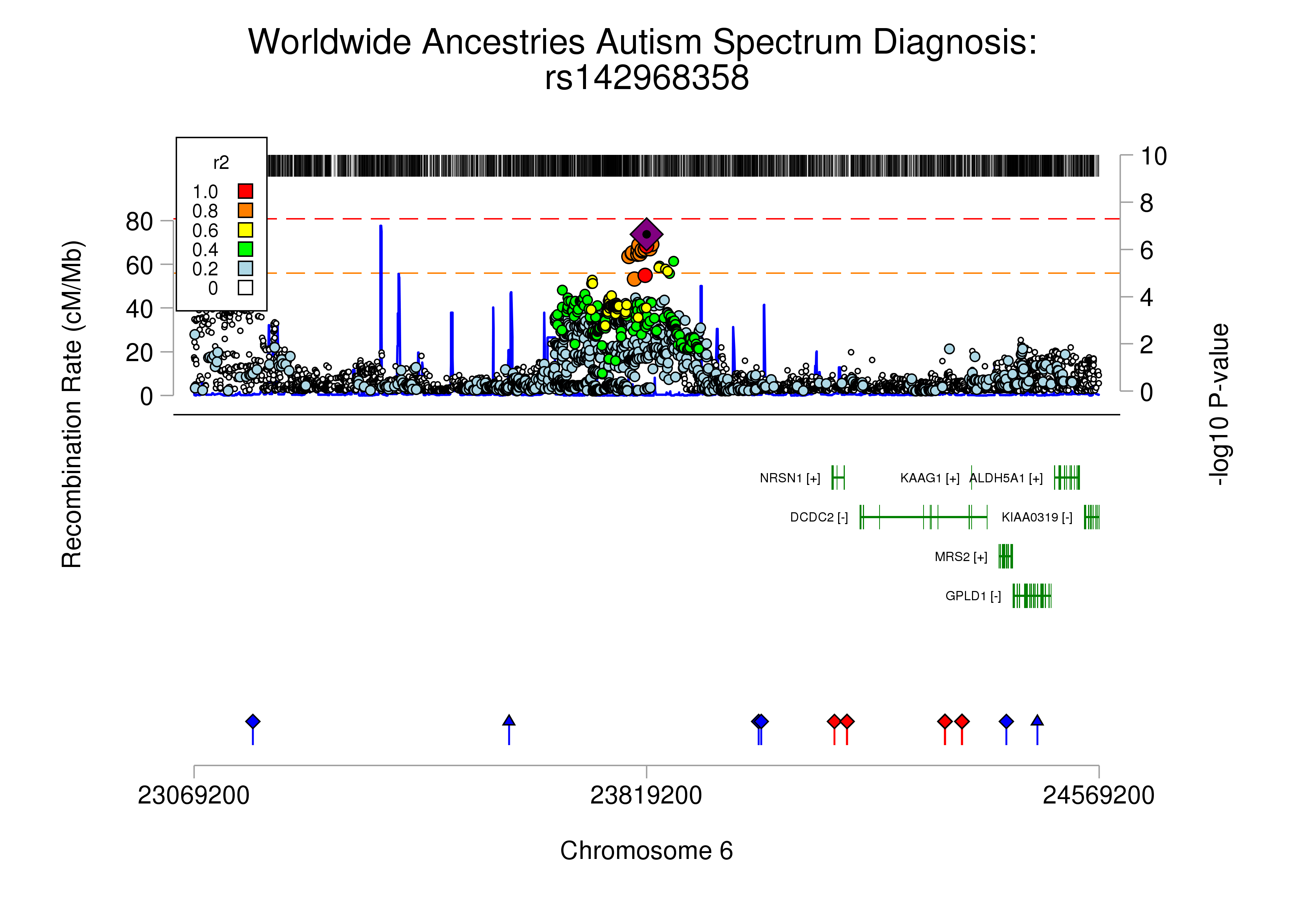


**Figure S6:** Association Locus plot for the index SNP rs142968358 in the Worldwide Autism GWAS.


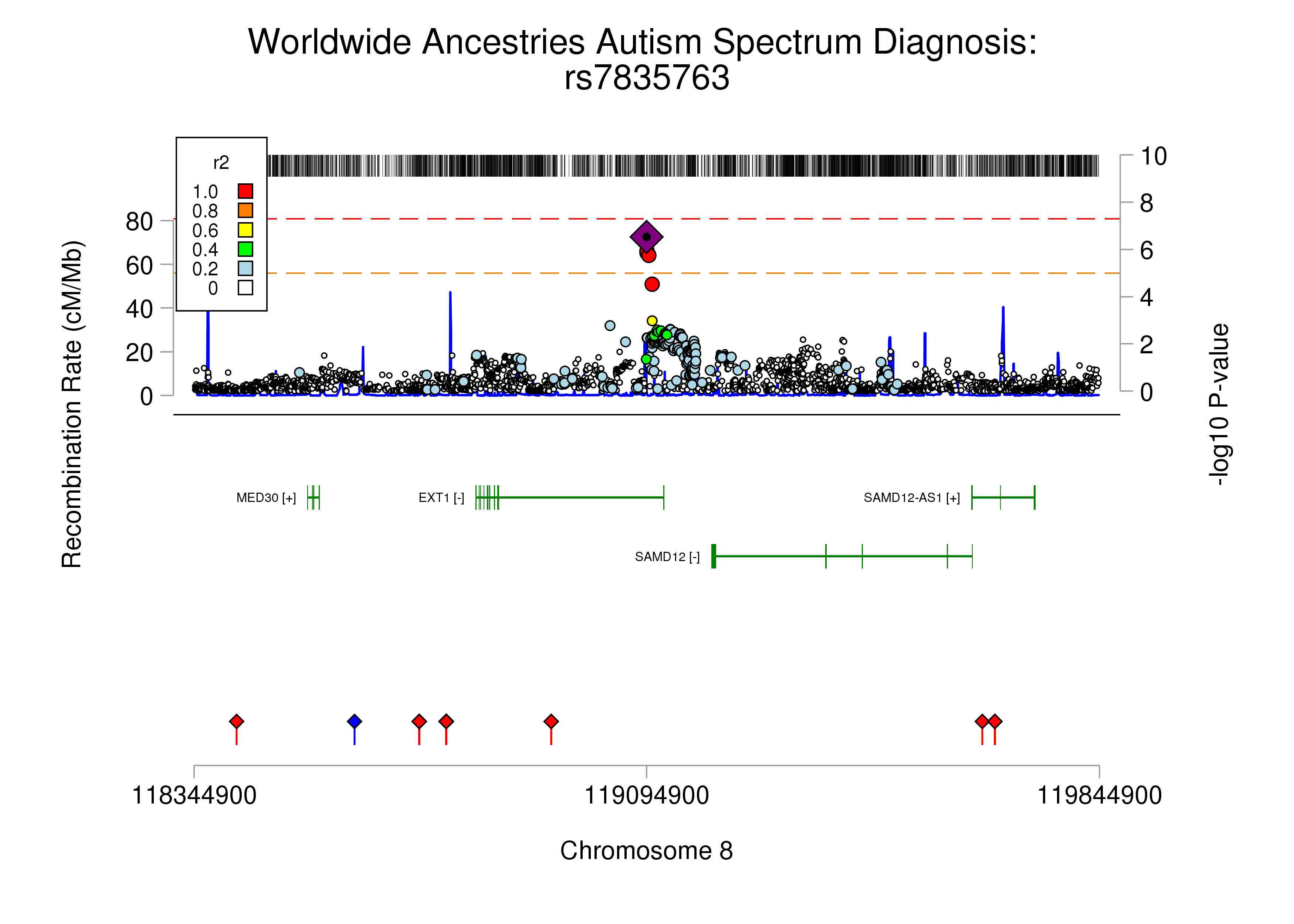


**Figure S7**: Association Locus plot for the index SNP rs115254791 in the Worldwide Autism GWAS.

**
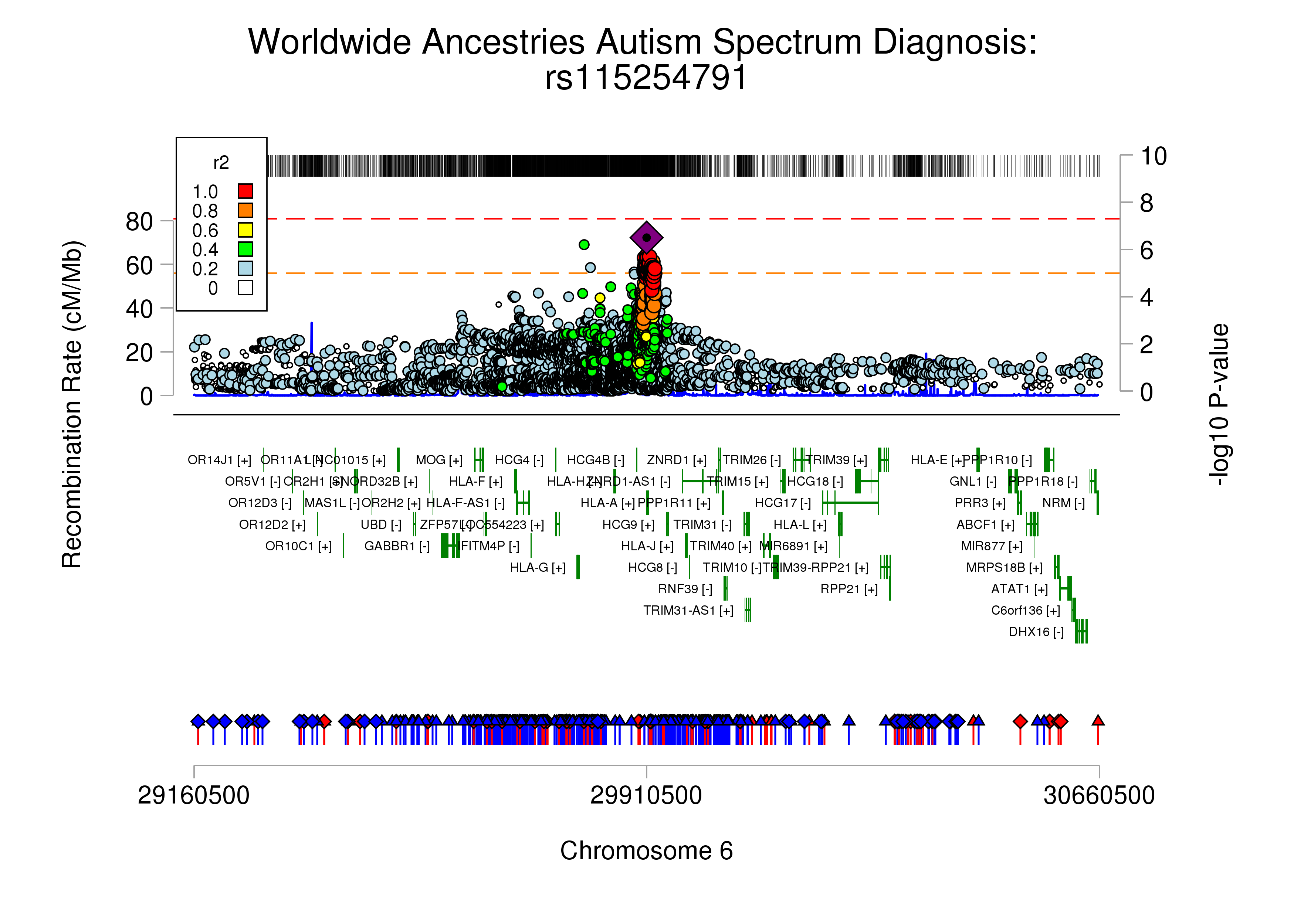
**

**Figure S8:** Association Locus plot for the index SNP rs7762549 in the Worldwide Autism GWAS.


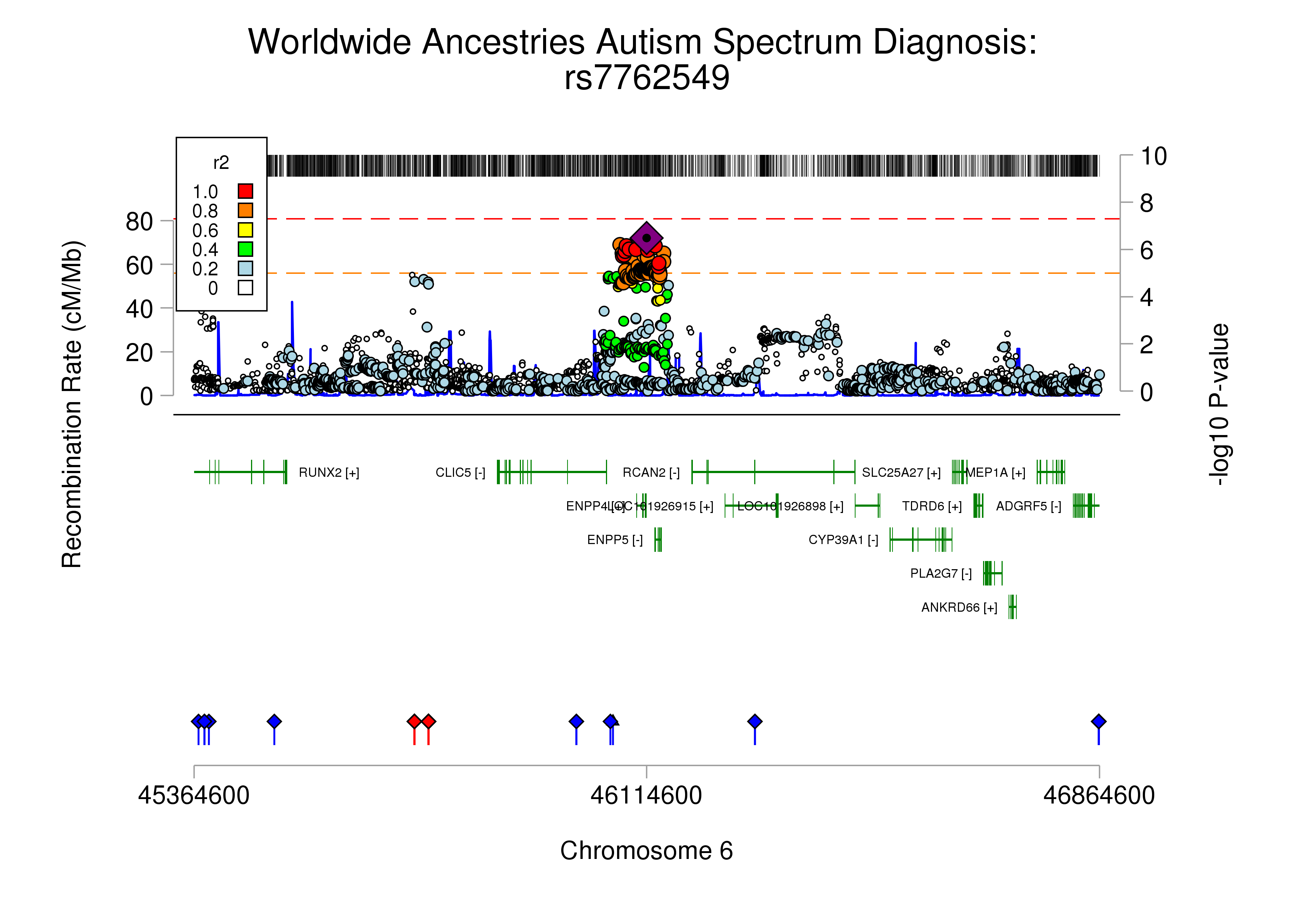


**Figure S9:** Association Locus plot for the index SNP rs7026354 in the European Autism GWAS


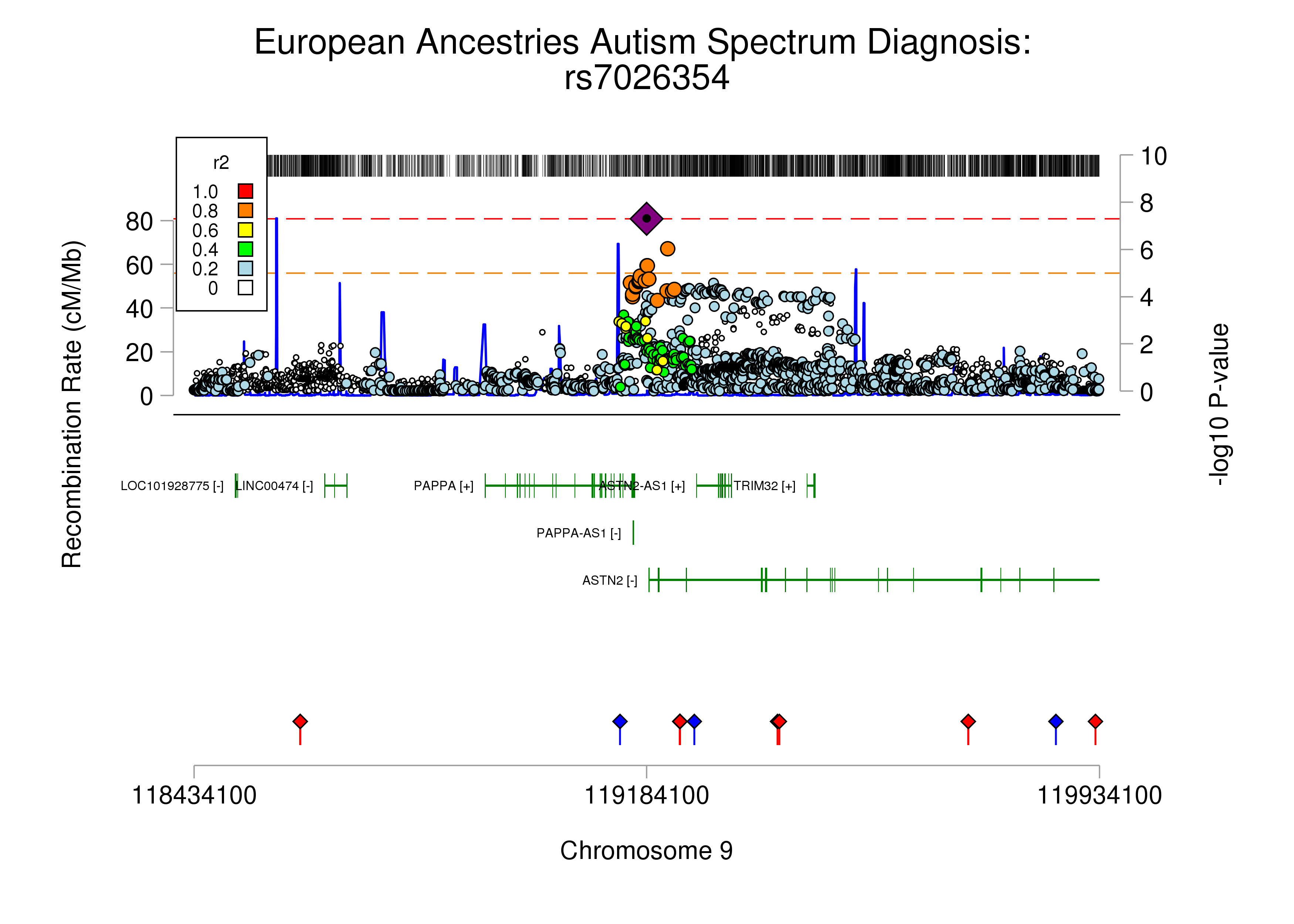


**Figure S10:** SNP-based heritability and genetic correlation estimated from the WW and EU Autism datasets. EU (European Ancestry ASD GWAS); WW/Aut (Worldwide Ancestry ASD GWAS); Schz (PGC2 Schizophrenia GWAS); RhAr (Rheumatoid Arthritis GWAS)


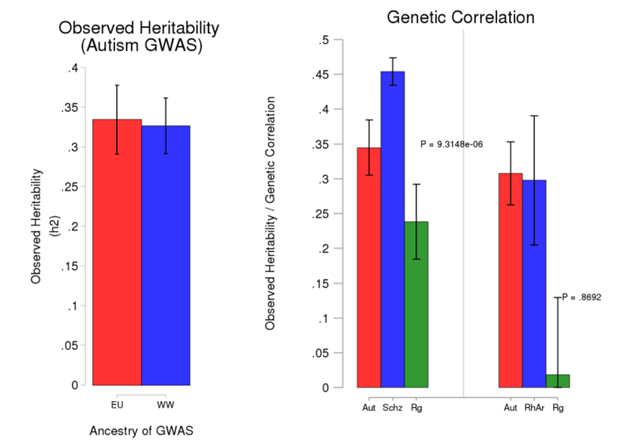


**Figure S11:** PP plot showing distribution of gene-based statistics


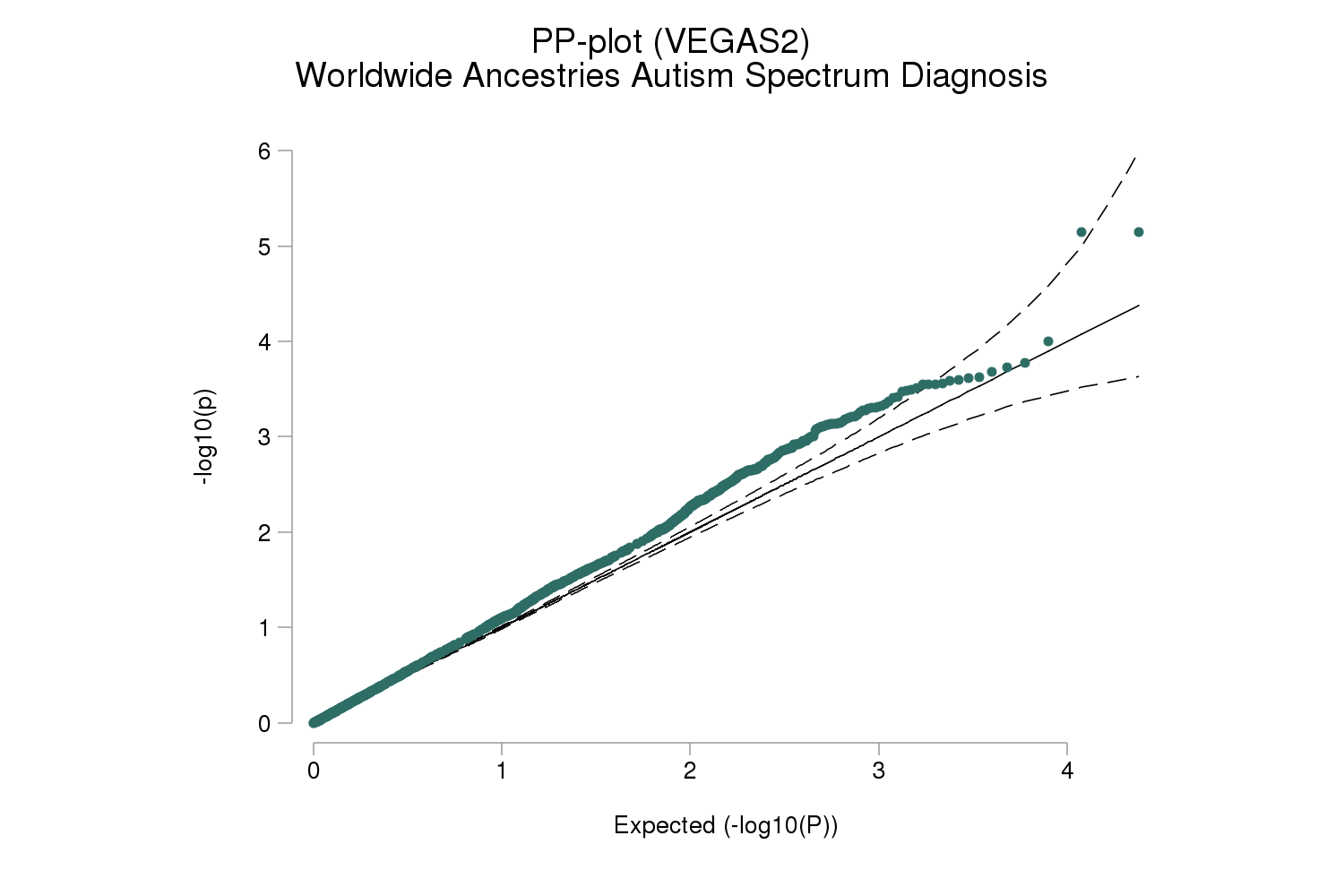


**Figure S12:** Manhattan Plot showing distribution of gene-based statistics


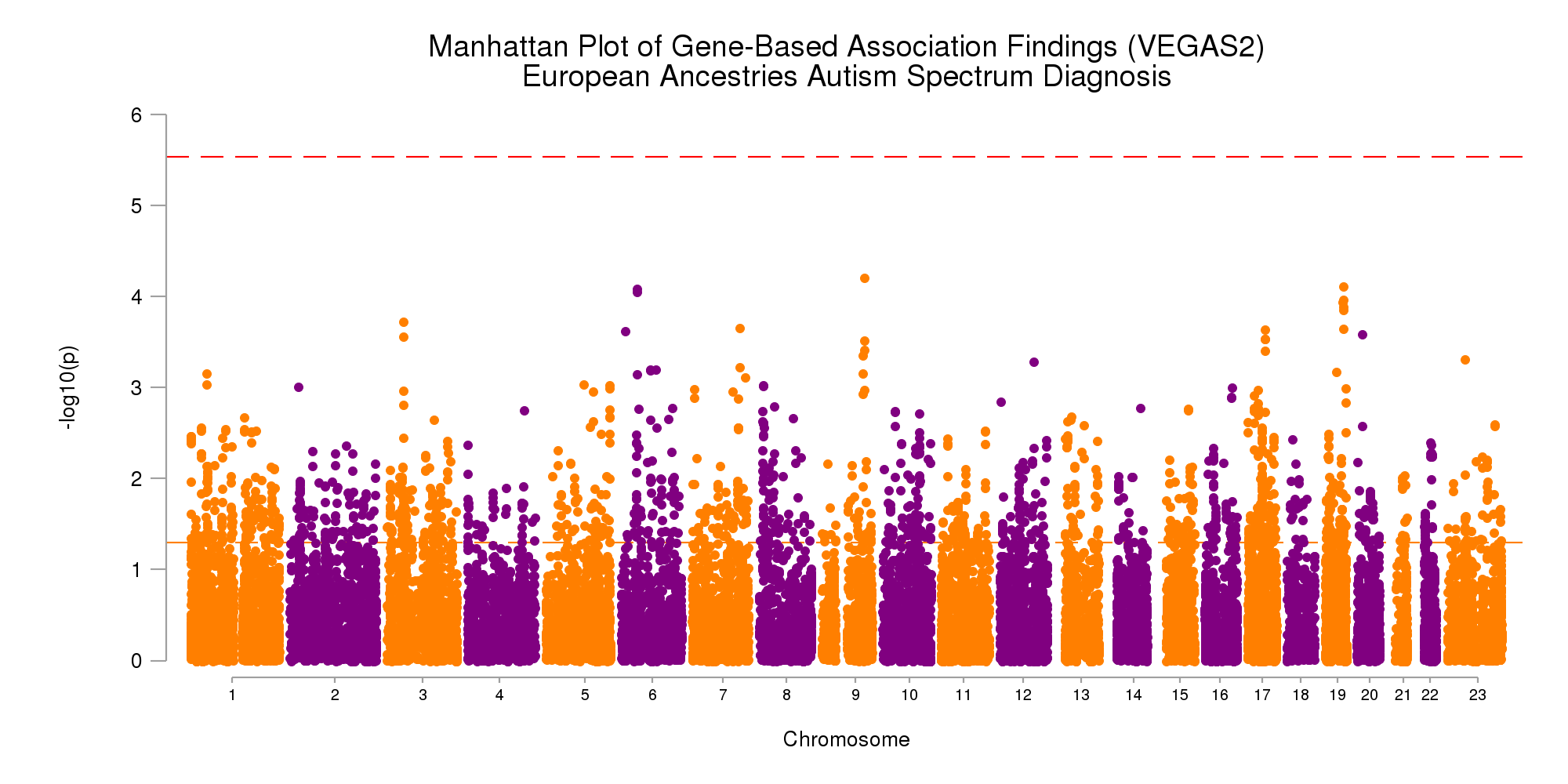


**Figure S13:** Association signal for the ASD and Schizophrenia GWAS across 10q24.32. ASD GWAS signals are depicted by red circles, with the index SNP from the meta-analyses in purple; Schizophrenia association signals are plotted in blue. Recombination Rate is plotted in black.


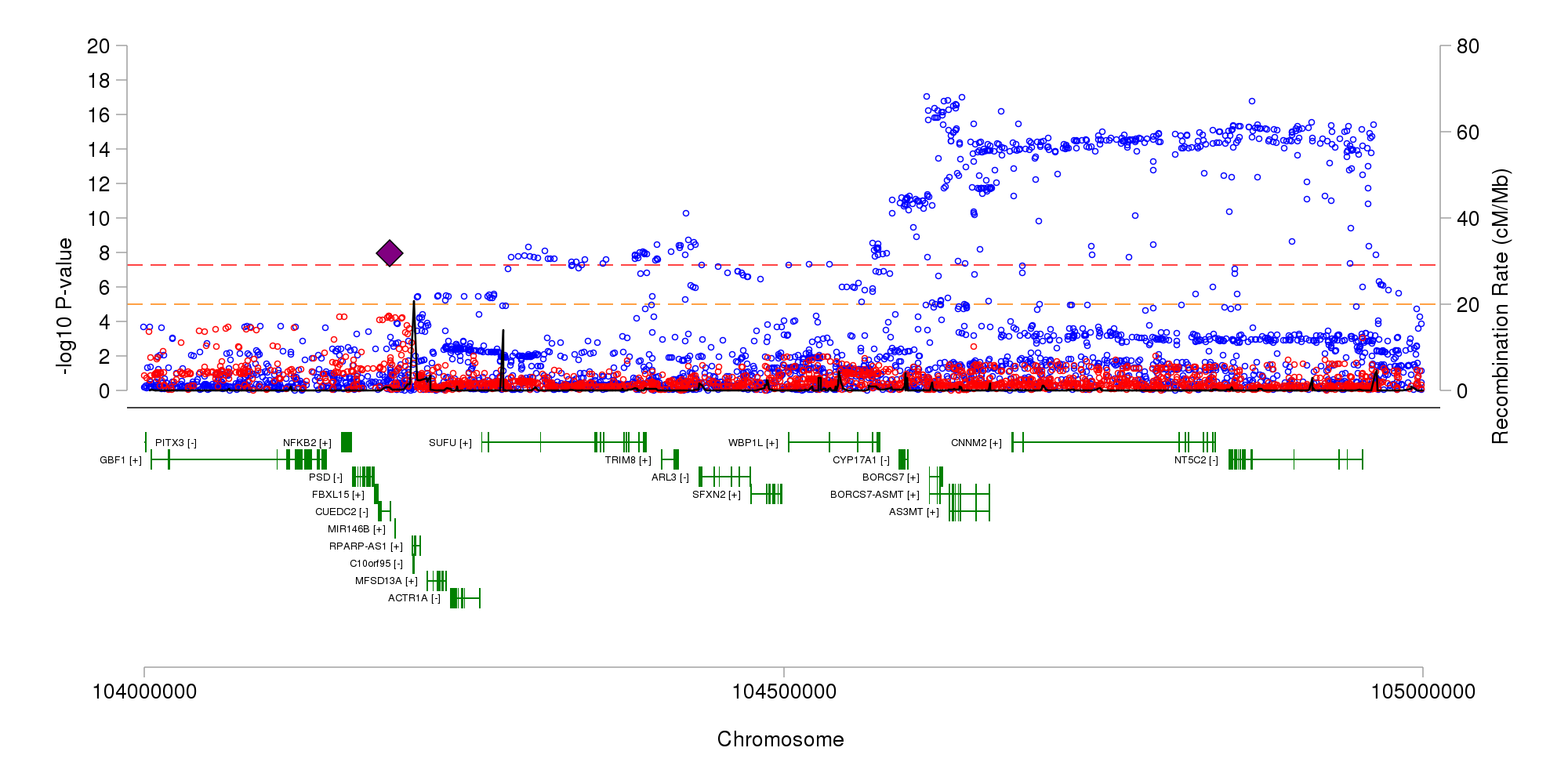


**Figure S14:** Distribution of Binomial Sign Test for the Direction of Effect in the Cross-Disorder Cohorts (Schizophrenia versus Autism; Schizophrenia versus Rheumatoid Arthritis).


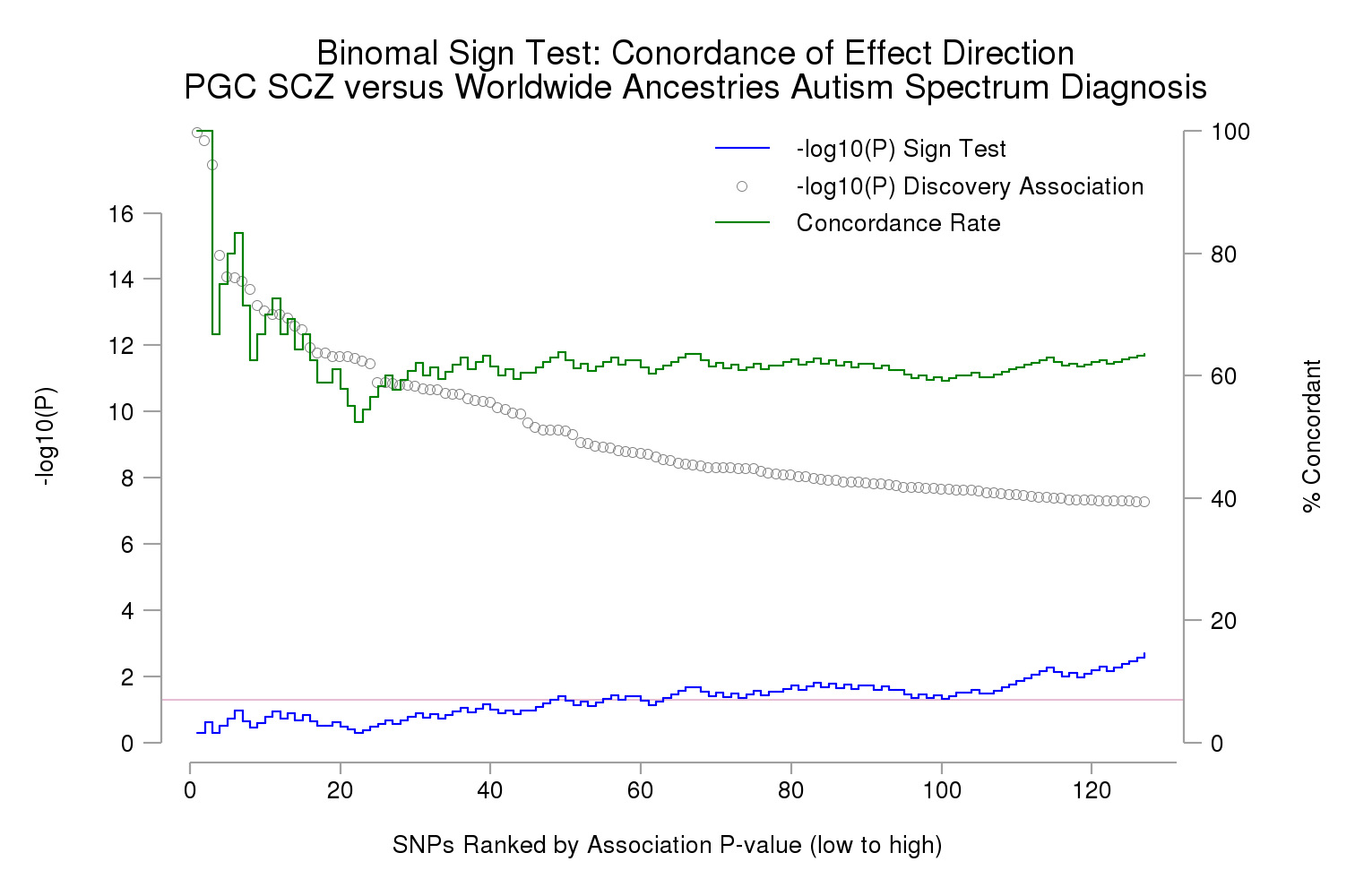

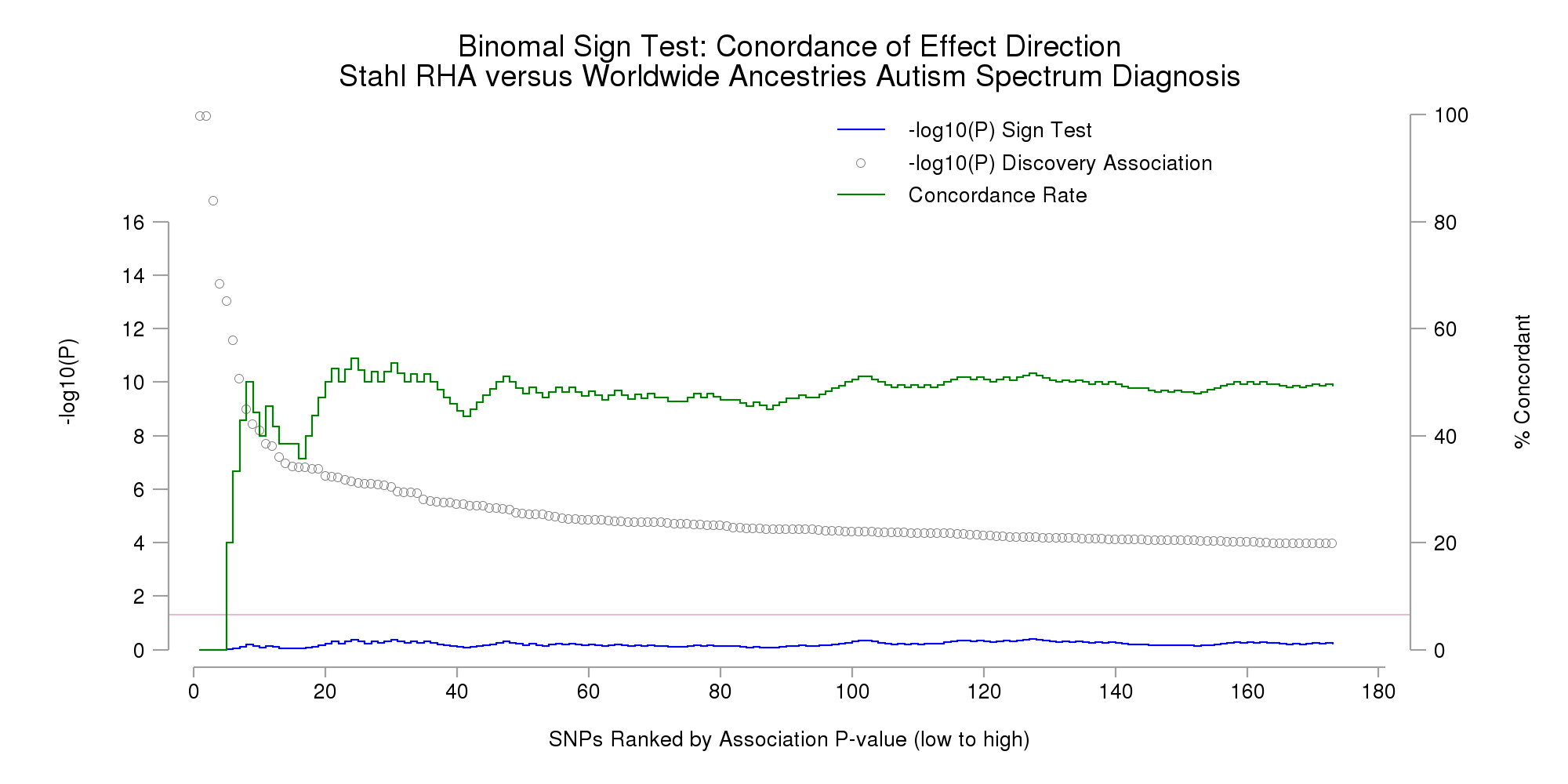


**Figure S15:** Manhattan Plot for combined PGC Autism and PGC Schizophrenia Meta - Analyses.


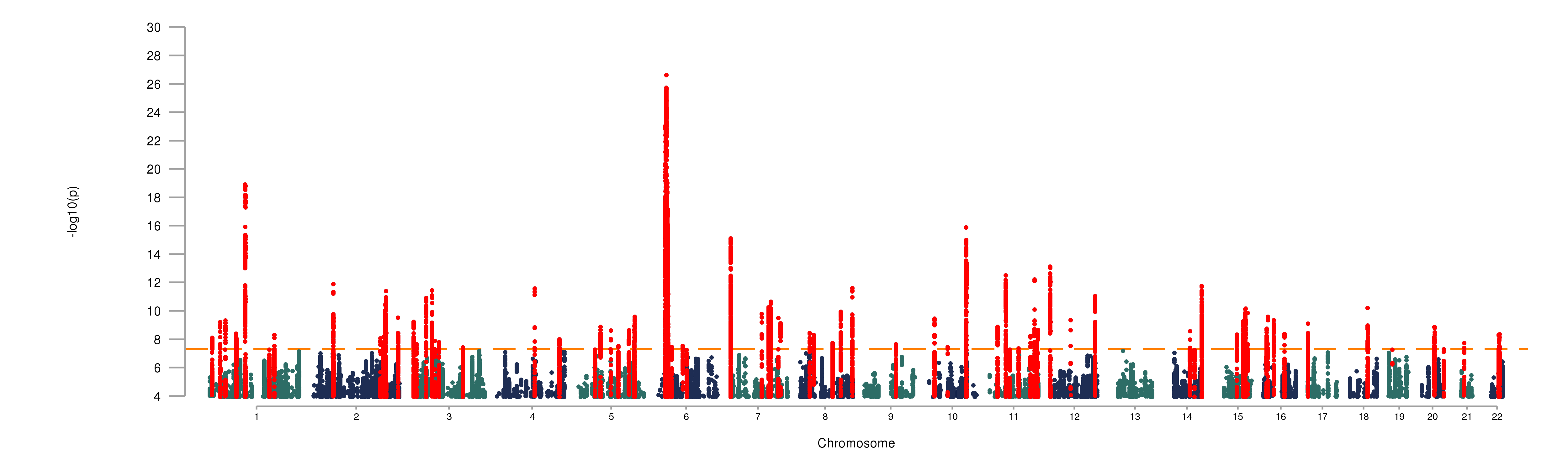


**Figure S16:** Association Locus Plot for the index SNPs in the combined Autism – Schizophrenia GWAS


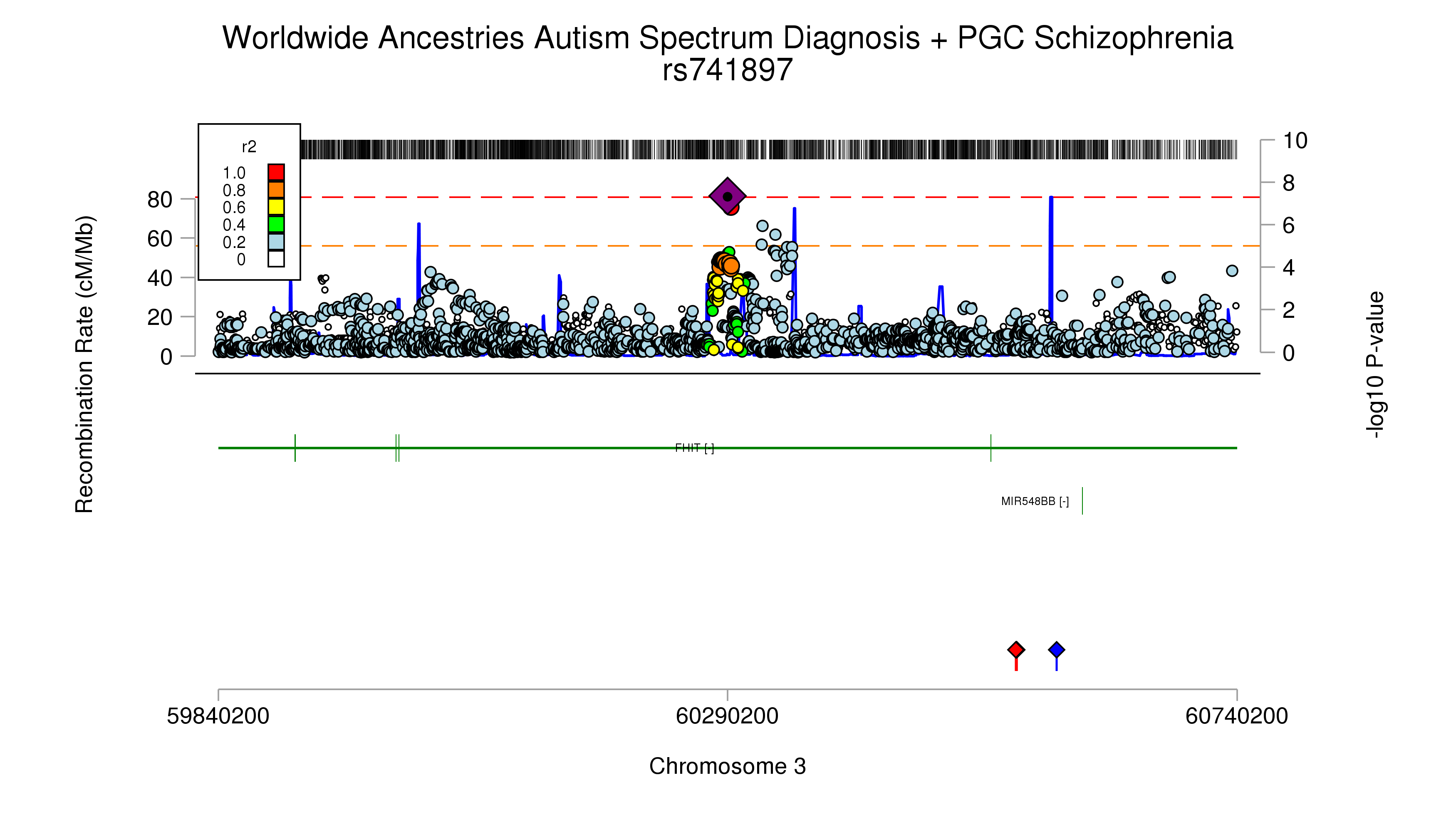

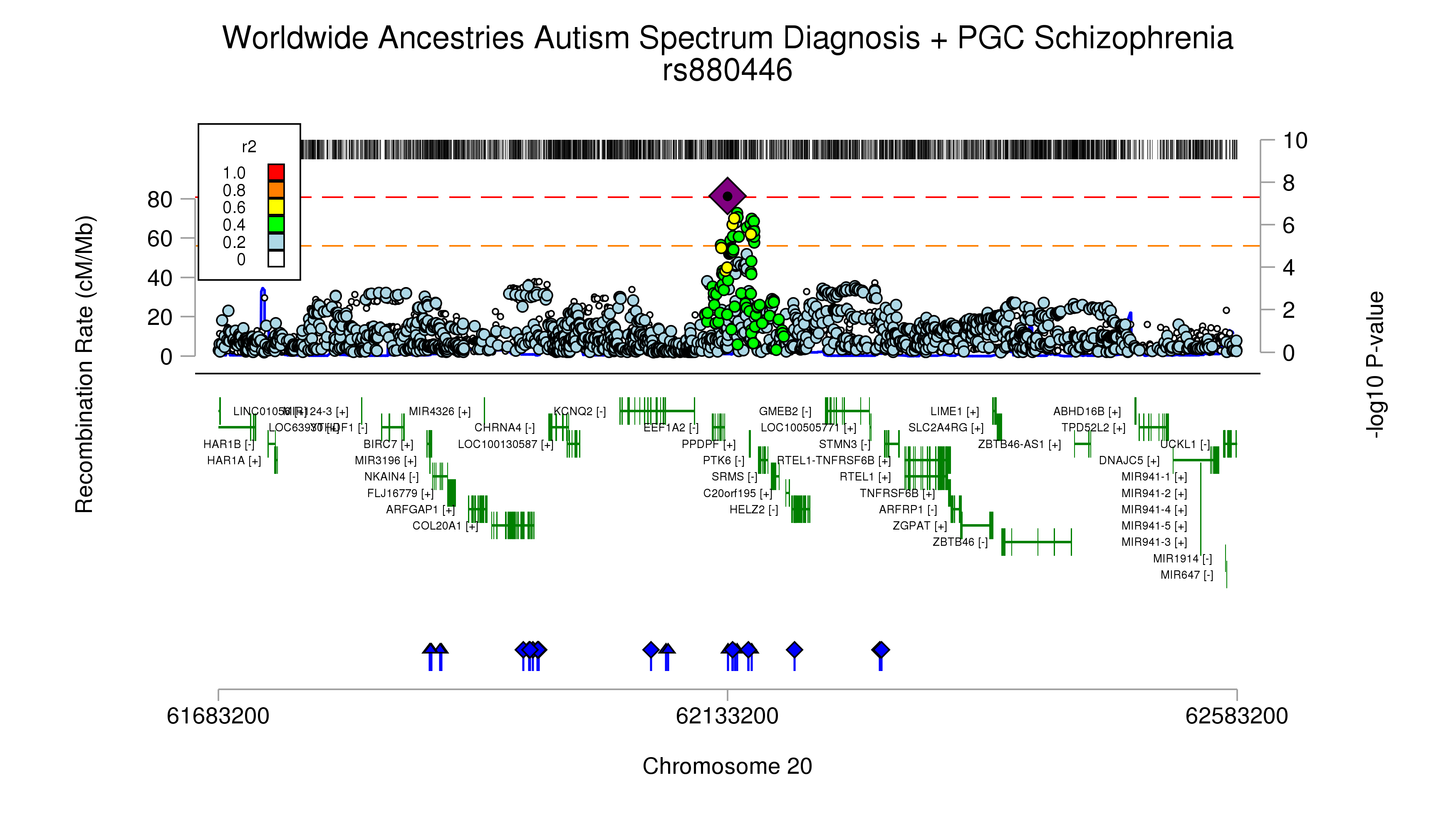

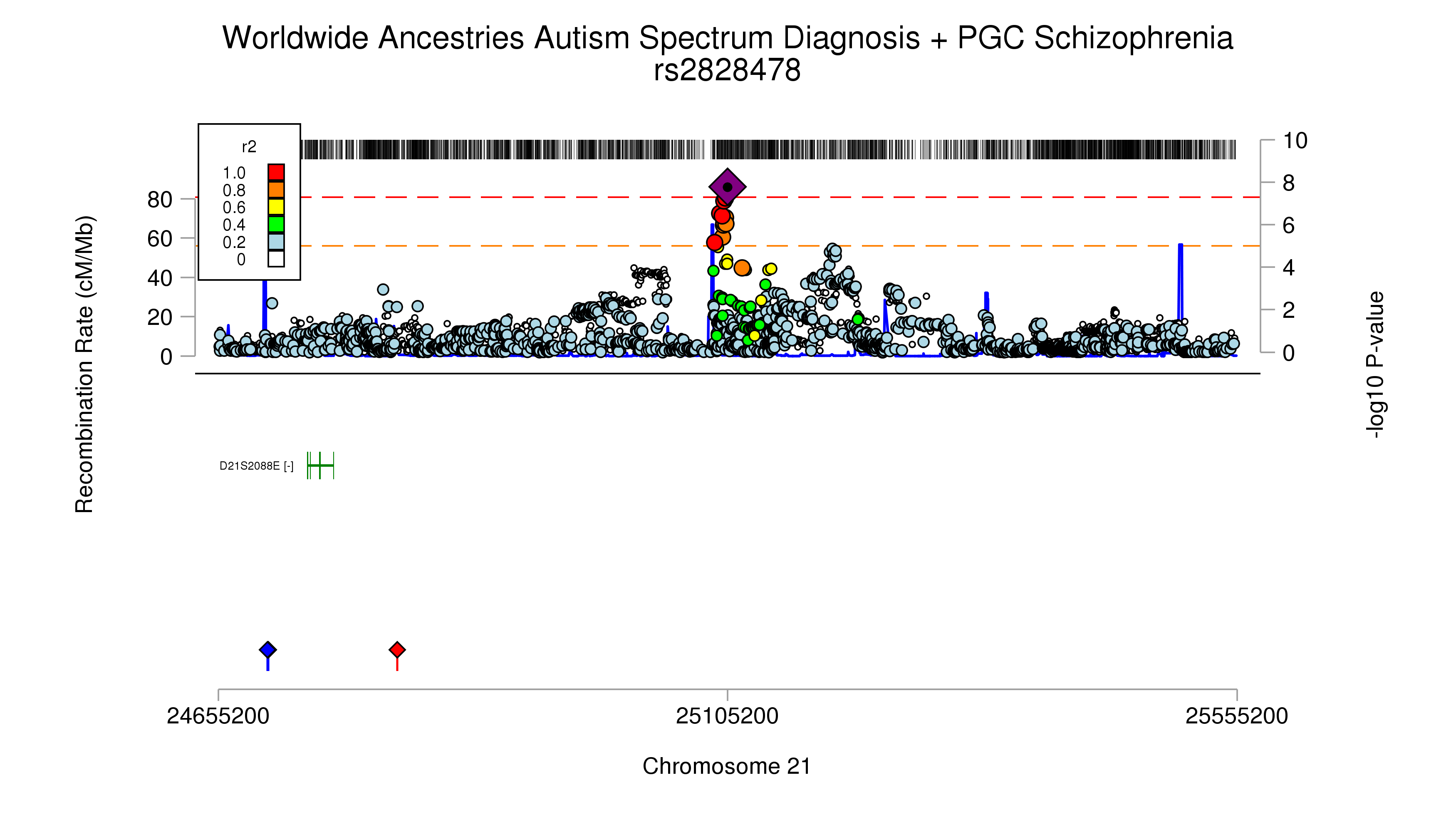

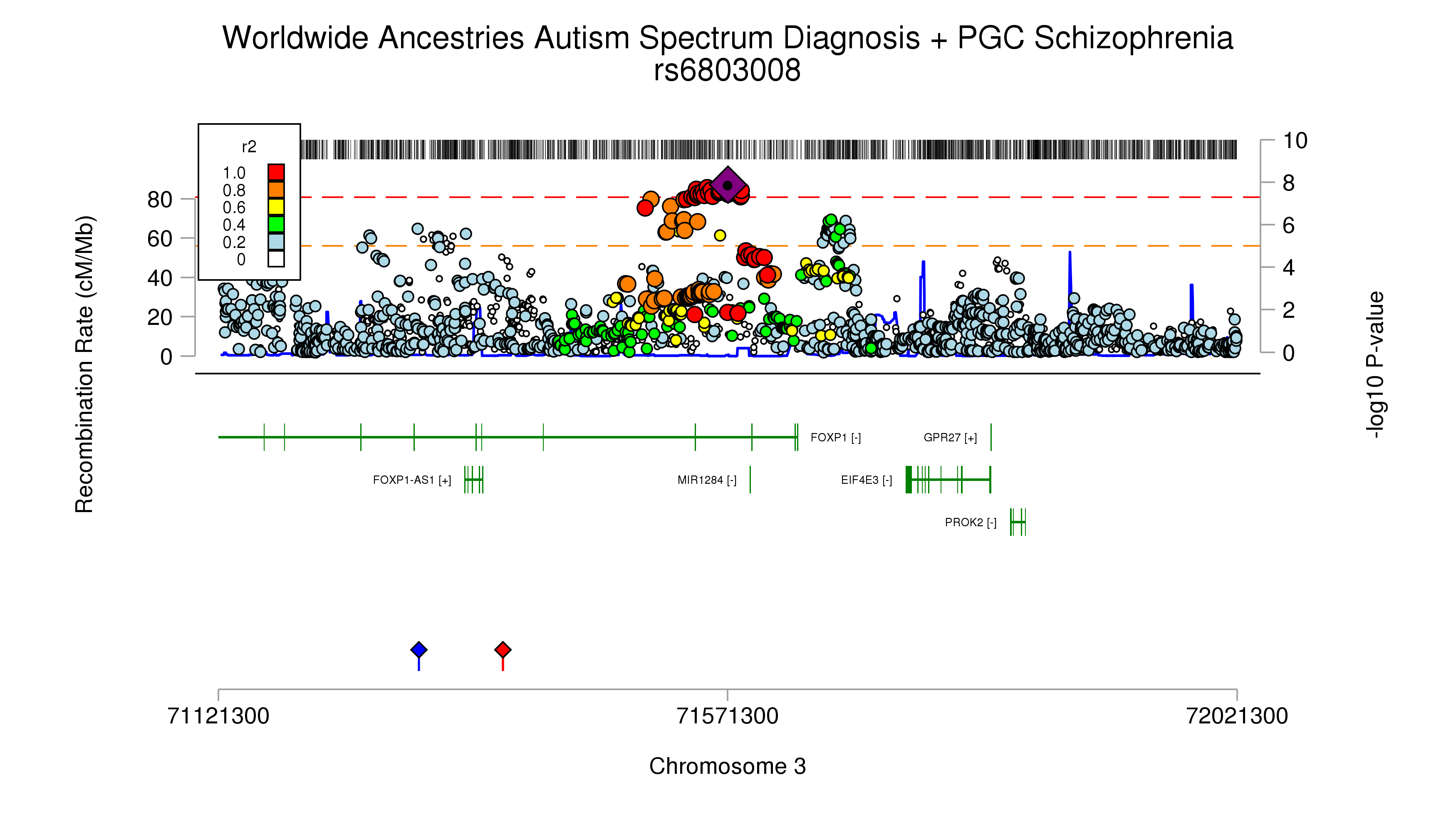

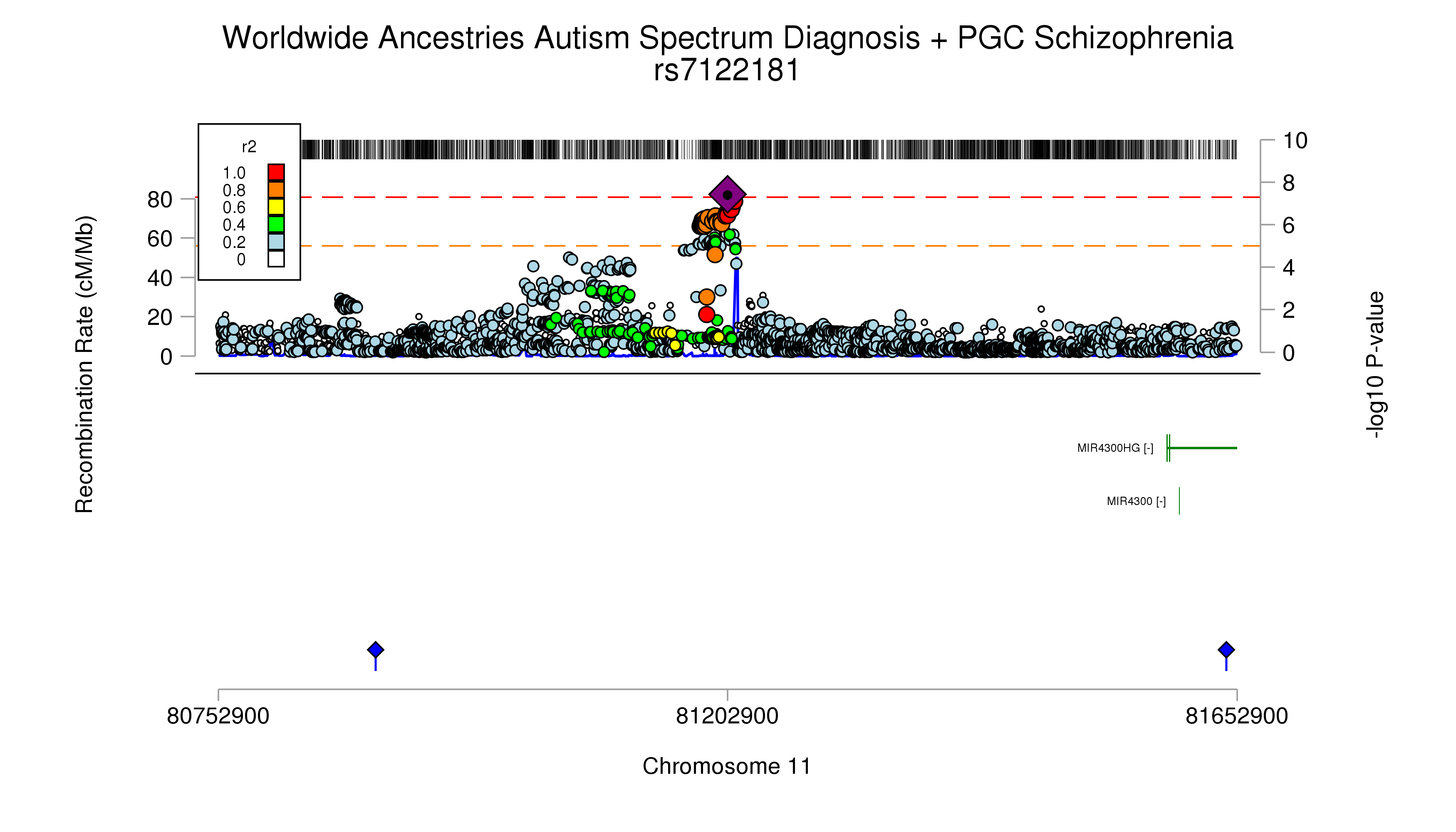

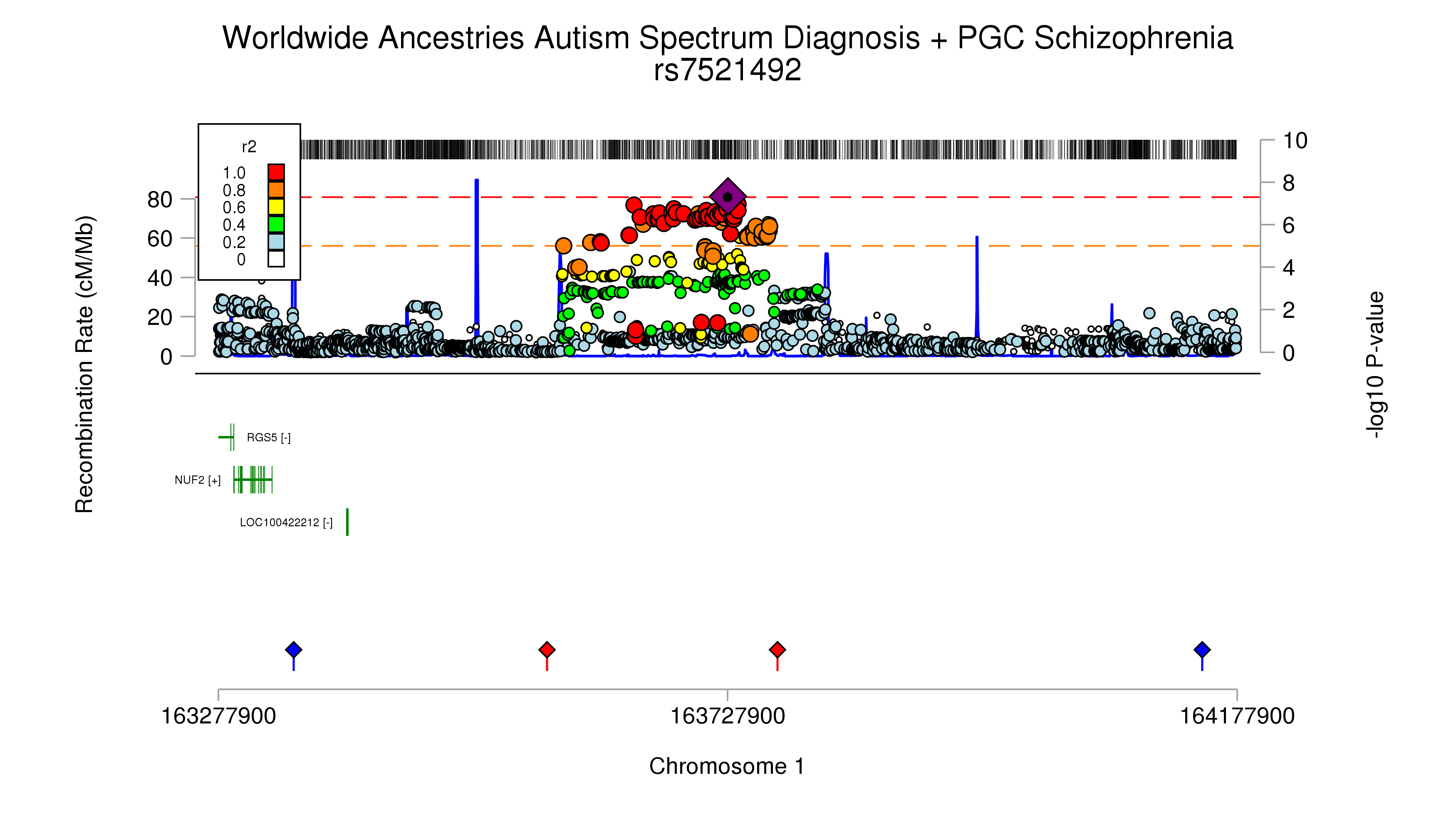

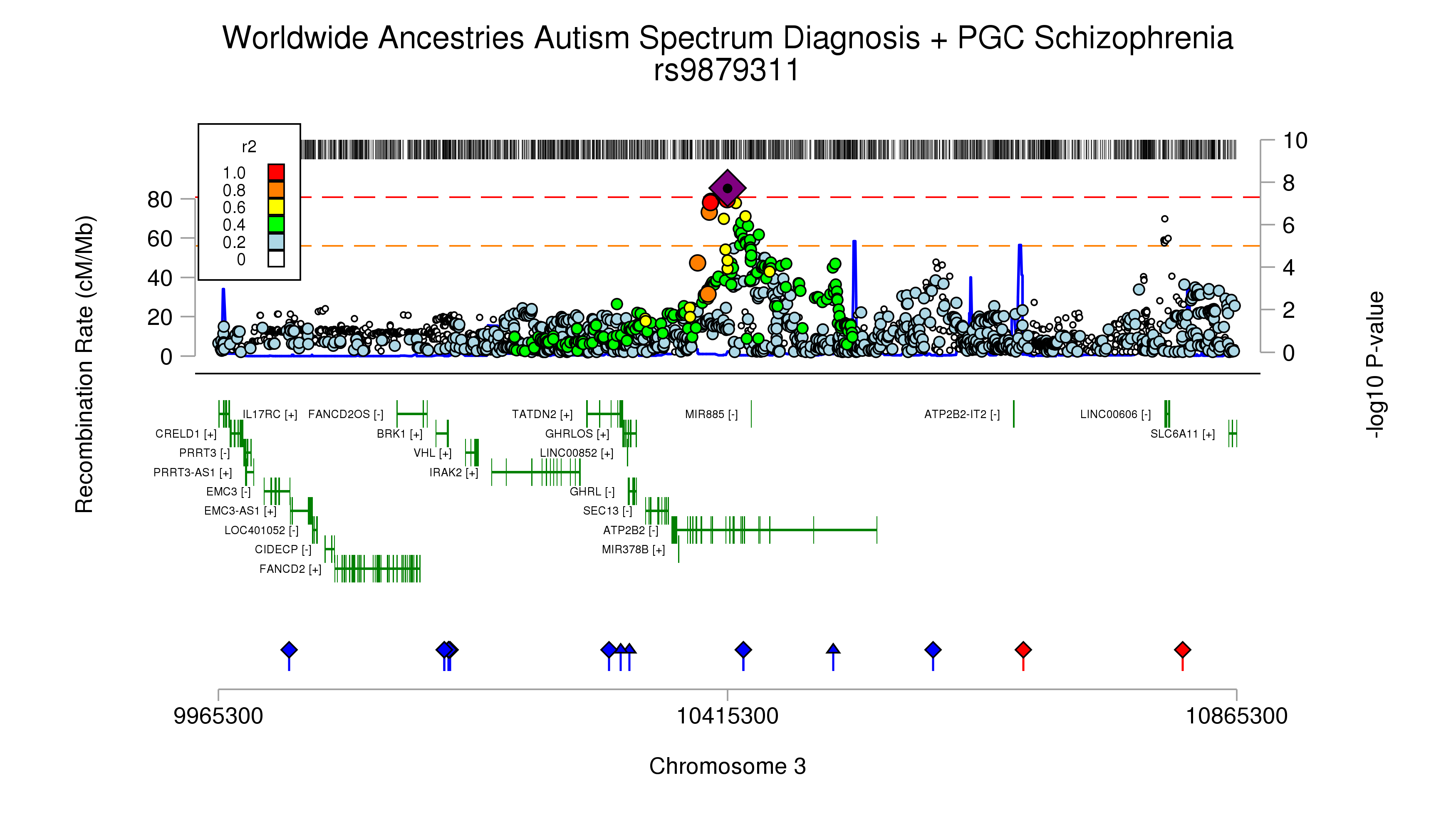

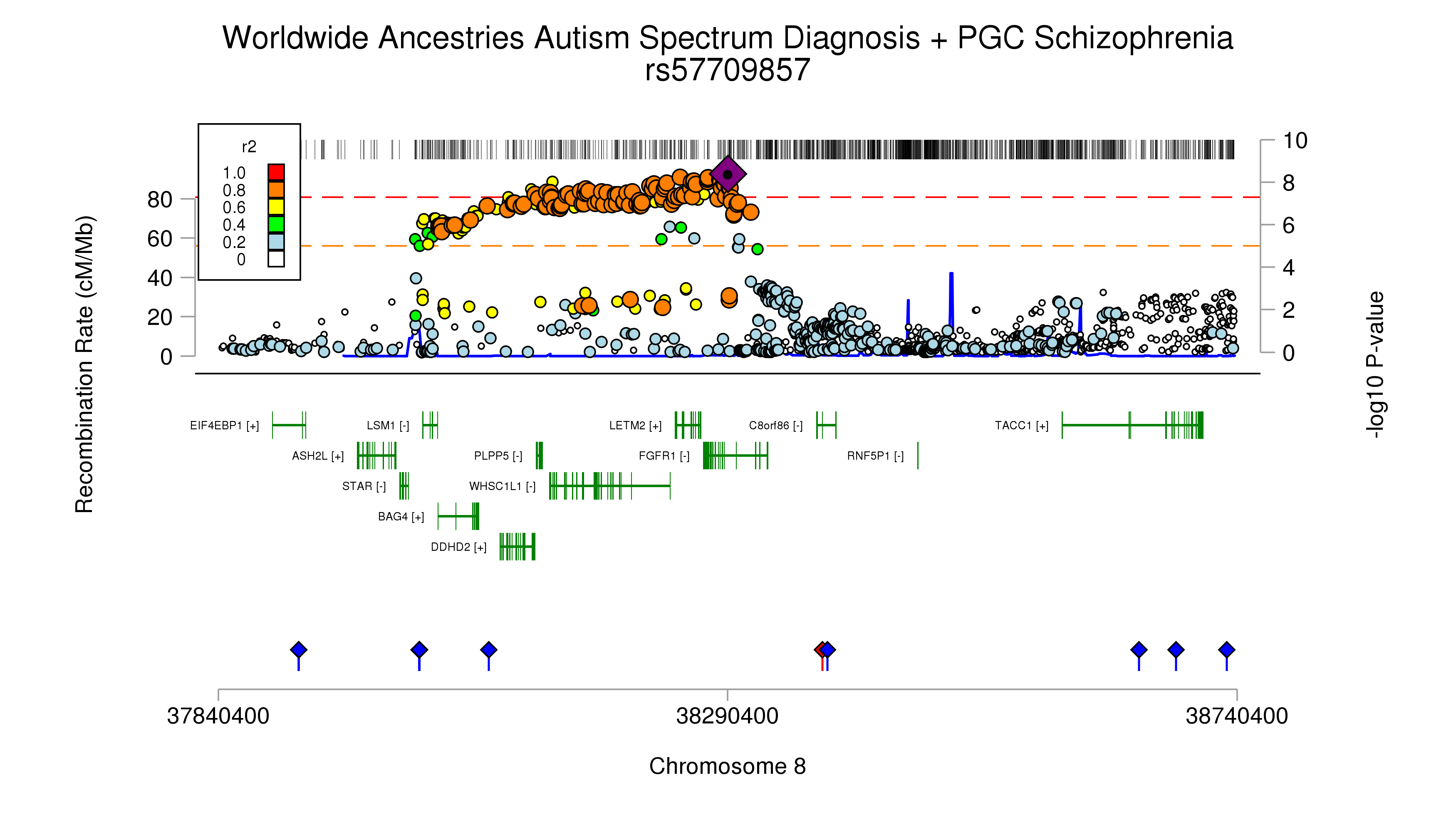

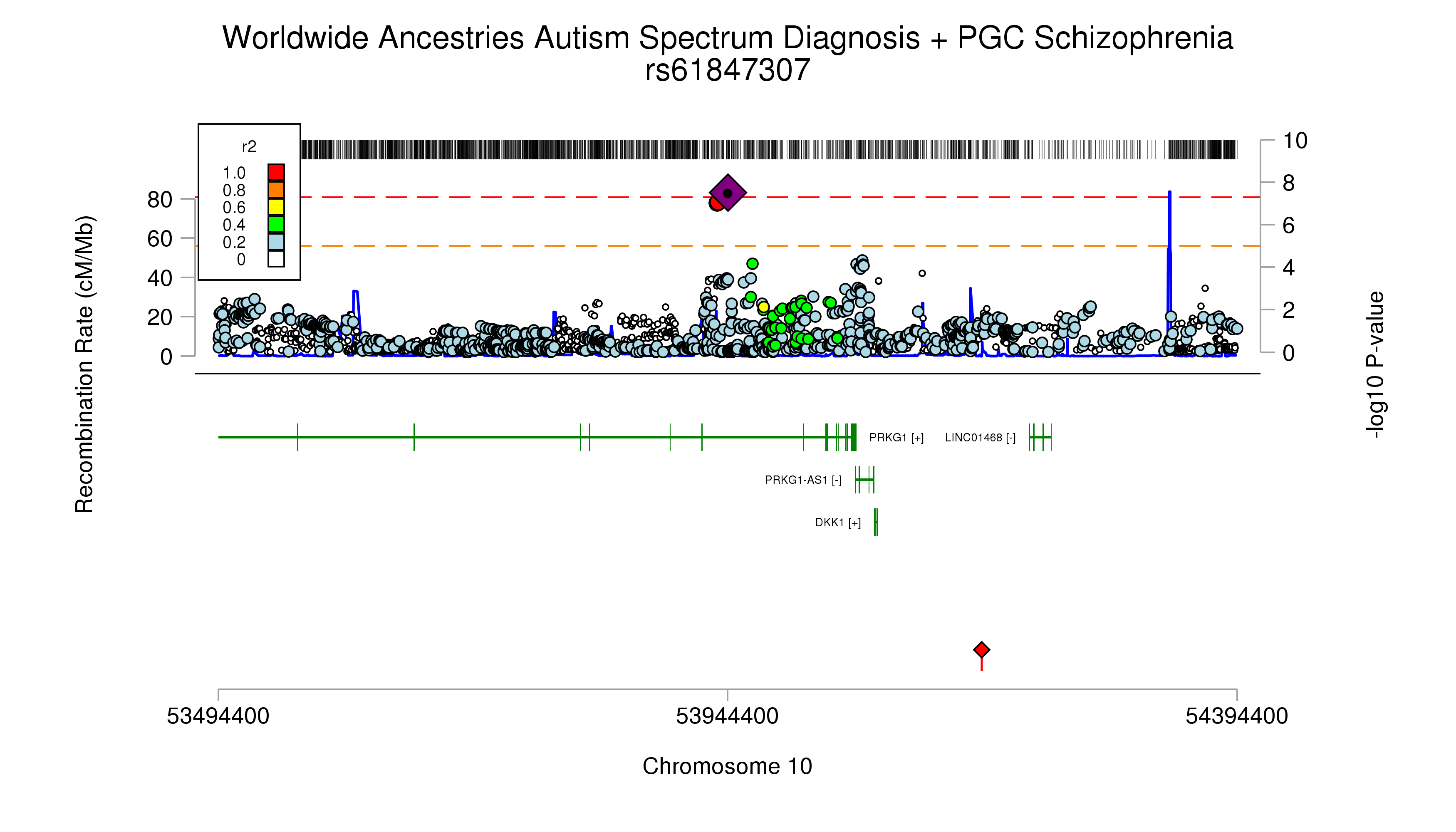

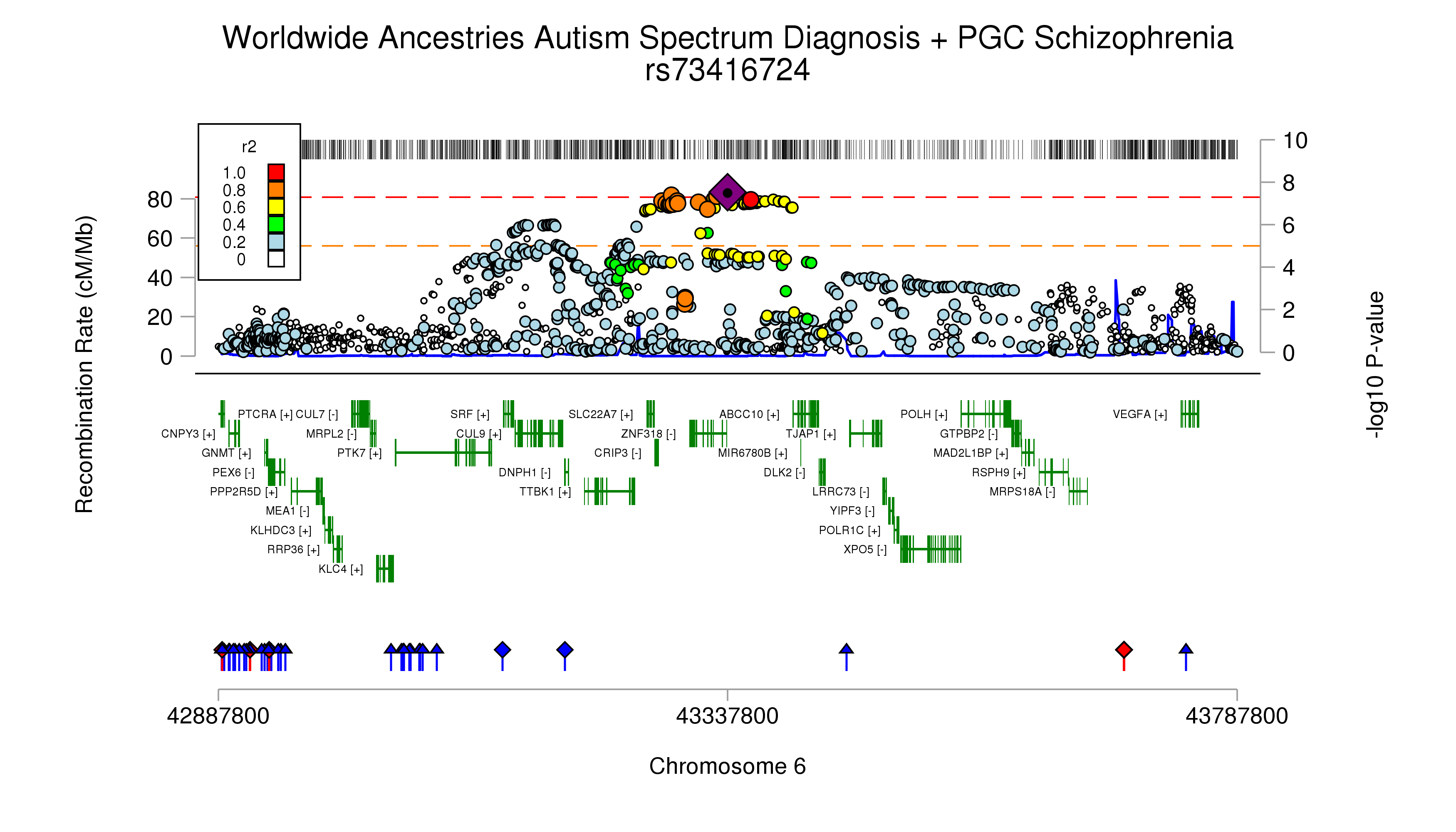


**Figure S17:** Principal Component (PC) Analysis of Raw Genotypes to describe Ancestry of the PGC ASD collection. The first 3 PCs are plotted; PCs derived from the 1KG panel are included for reference and orientation. Legend: black (1KG – European subset), blue (1KG – Asian subset), green (1KG – African subset, purple (PGC-ASD – European Subset), red (PGC-ASD – non-European subset).


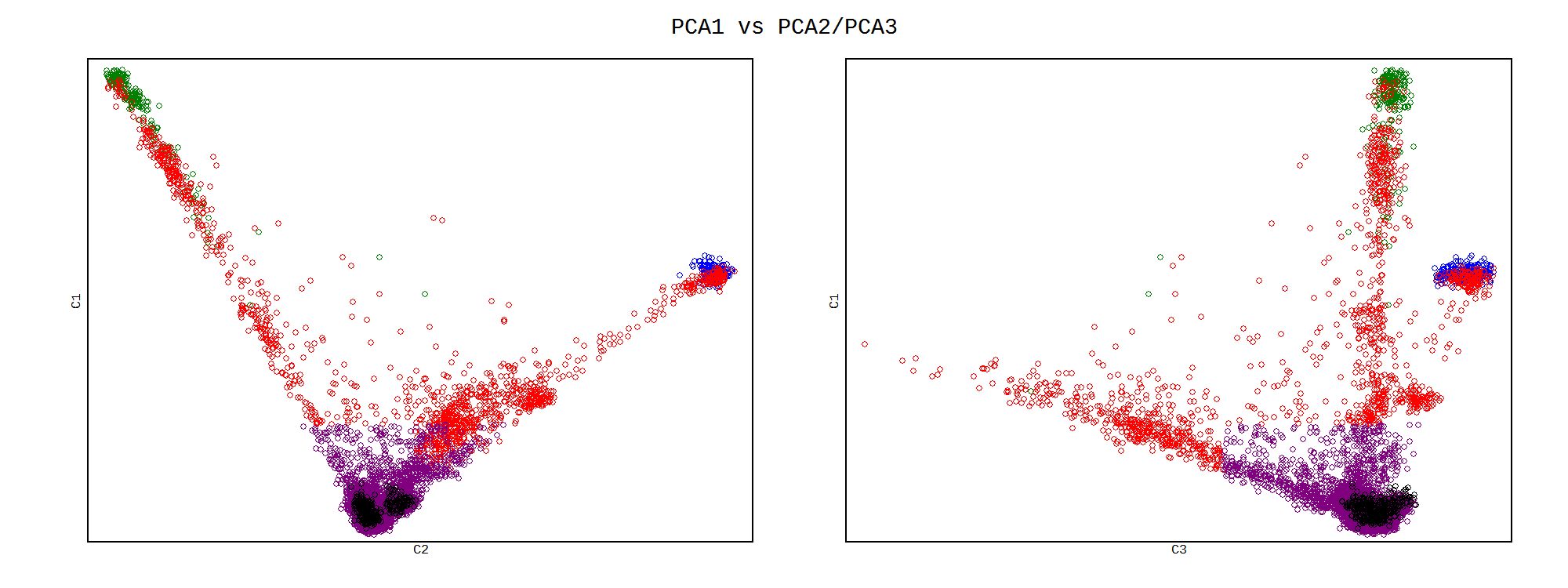


**Figure S18:** Schematic of the analytic approach undertaken.


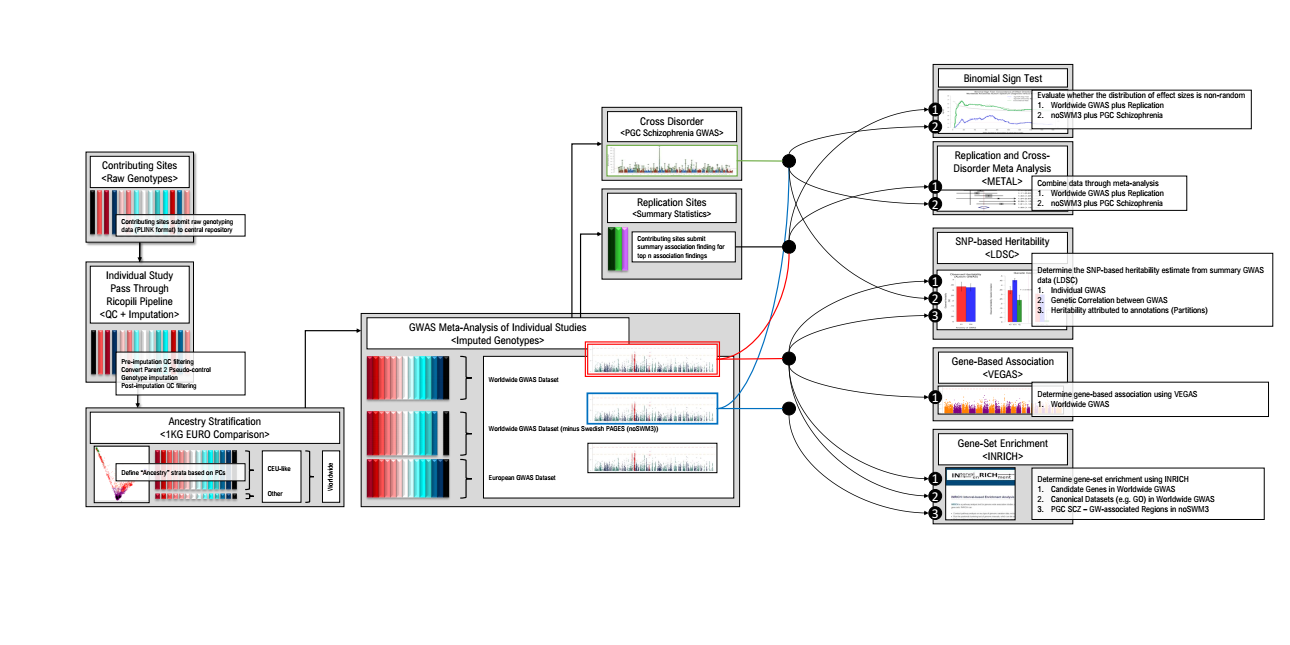


# References

1. Anney R, Klei L, Pinto D, Regan R, Conroy J, Magalhaes TR, Correia C, Abrahams BS, Sykes N, Pagnamenta AT *et al*: **A genome-wide scan for common alleles affecting risk for autism**. *Human molecular genetics* 2010, **19**(20):4072-4082.

2. Anney R, Klei L, Pinto D, Almeida J, Bacchelli E, Baird G, Bolshakova N, Bolte S, Bolton PF, Bourgeron T *et al*: **Individual common variants exert weak effects on the risk for autism spectrum disorders**. *Human molecular genetics* 2012, **21**(21):4781-4792.

3. Lajonchere CM, Consortium A: **Changing the landscape of autism research: the autism genetic resource exchange**. *Neuron* 2010, **68**(2):187-191.

4. Geschwind DH, Sowinski J, Lord C, Iversen P, Shestack J, Jones P, Ducat L, Spence SJ, Committee AS: **The autism genetic resource exchange: a resource for the study of autism and related neuropsychiatric conditions**. *American journal of human genetics* 2001, **69**(2):463-466.

5. Weiss LA, Arking DE, Daly MJ, Chakravarti A: **A genome-wide linkage and association scan reveals novel loci for autism**. *Nature* 2009, **461**(7265):802-808.

6. Gaugler T, Klei L, Sanders SJ, Bodea CA, Goldberg AP, Lee AB, Mahajan M, Manaa D, Pawitan Y, Reichert J *et al*: **Most genetic risk for autism resides with common variation**. *Nature genetics* 2014, **46**(8):881-885.

7. Chaste P, Klei L, Sanders SJ, Hus V, Murtha MT, Lowe JK, Willsey AJ, Moreno-De-Luca D, Yu TW, Fombonne E *et al*: **A genome-wide association study of autism using the Simons Simplex Collection: Does reducing phenotypic heterogeneity in autism increase genetic homogeneity?** *Biological psychiatry* 2015, **77**(9):775-784.

8. Lord C, Rutter M, Le Couteur A: **Autism Diagnostic Interview-Revised: a revised version of a diagnostic interview for caregivers of individuals with possible pervasive developmental disorders**. *Journal of autism and developmental disorders* 1994, **24**(5):659-685.

9. Lord C, Rutter M, Goode S, Heemsbergen J, Jordan H, Mawhood L, Schopler E: **Autism diagnostic observation schedule: a standardized observation of communicative and social behavior**. *Journal of autism and developmental disorders* 1989, **19**(2):185-212.

10. Pinto D, Pagnamenta AT, Klei L, Anney R, Merico D, Regan R, Conroy J, Magalhaes TR, Correia C, Abrahams BS *et al*: **Functional impact of global rare copy number variation in autism spectrum disorders**. *Nature* 2010, **466**(7304):368-372.

11. Pinto D, Delaby E, Merico D, Barbosa M, Merikangas A, Klei L, Thiruvahindrapuram B, Xu X, Ziman R, Wang Z *et al*: **Convergence of genes and cellular pathways dysregulated in autism spectrum disorders**. *American journal of human genetics* 2014, **94**(5):677-694.

12. Szatmari P, Paterson AD, Zwaigenbaum L, Roberts W, Brian J, Liu X-Q, Vincent JB, Skaug JL, Thompson AP, Senman L *et al*: **Mapping autism risk loci using genetic linkage and chromosomal rearrangements**. *Nature genetics* 2007, **39**(3):319-328.

13. Gauthier J, Joober R, Dube MP, St-Onge J, Bonnel A, Gariepy D, Laurent S, Najafee R, Lacasse H, St-Charles L *et al*: **Autism spectrum disorders associated with X chromosome markers in French-Canadian males**. *Molecular psychiatry* 2006, **11**(2):206-213.

14. Berument SK, Rutter M, Lord C, Pickles A, Bailey A: **Autism screening questionnaire: diagnostic validity**. *The British journal of psychiatry : the journal of mental science* 1999, **175**:444-451.

15. Teslovich TM, Musunuru K, Smith AV, Edmondson AC, Stylianou IM, Koseki M, Pirruccello JP, Ripatti S, Chasman DI, Willer CJ *et al*: **Biological, clinical and population relevance of 95 loci for blood lipids**. *Nature* 2010, **466**(7307):707-713.

16. Auranen M, Vanhala R, Varilo T, Ayers K, Kempas E, Ylisaukko-Oja T, Sinsheimer JS, Peltonen L, Jarvela I: **A genomewide screen for autism-spectrum disorders: evidence for a major susceptibility locus on chromosome 3q25-27**. *American journal of human genetics* 2002, **71**(4):777-790.

17. Ylisaukko-oja T, Nieminen-von Wendt T, Kempas E, Sarenius S, Varilo T, von Wendt L, Peltonen L, Jarvela I: **Genome-wide scan for loci of Asperger syndrome**. *Molecular psychiatry* 2004, **9**(2):161-168.

18. Rehnstrom K, Ylisaukko-oja T, Nieminen-von Wendt T, Sarenius S, Kallman T, Kempas E, von Wendt L, Peltonen L, Jarvela I: **Independent replication and initial fine mapping of 3p21-24 in Asperger syndrome**. *J Med Genet* 2006, **43**(2):e6.

19. Kilpinen H, Ylisaukko-oja T, Rehnstrom K, Gaal E, Turunen JA, Kempas E, von Wendt L, Varilo T, Peltonen L: **Linkage and linkage disequilibrium scan for autism loci in an extended pedigree from Finland**. *Human molecular genetics* 2009, **18**(15):2912-2921.

20. Lampi KM, Sourander A, Gissler M, Niemela S, Rehnstrom K, Pulkkinen E, Peltonen L, Von Wendt L: **Brief report: validity of Finnish registry-based diagnoses of autism with the ADI-R**. *Acta Paediatr* 2010, **99**(9):1425-1428.

21. Fischbach GD, Lord C: **The Simons Simplex Collection: a resource for identification of autism genetic risk factors**. *Neuron* 2010, **68**(2):192-195.

22. Bertoglio K, Jill James S, Deprey L, Brule N, Hendren RL: **Pilot study of the effect of methyl B12 treatment on behavioral and biomarker measures in children with autism**. *Journal of alternative and complementary medicine* 2010, **16**(5):555-560.

23. Lit L, Sharp FR, Bertoglio K, Stamova B, Ander BP, Sossong AD, Hendren RL: **Gene expression in blood is associated with risperidone response in children with autism spectrum disorders**. *The pharmacogenomics journal* 2012, **12**(5):368-371.

24. Gorrindo P, Williams KC, Lee EB, Walker LS, McGrew SG, Levitt P: **Gastrointestinal dysfunction in autism: parental report, clinical evaluation, and associated factors**. *Autism research : official journal of the International Society for Autism Research* 2012, **5**(2):101-108.

25. Hertz-Picciotto I, Croen LA, Hansen R, Jones CR, van de Water J, Pessah IN: **The CHARGE study: an epidemiologic investigation of genetic and environmental factors contributing to autism**. *Environmental health perspectives* 2006, **114**(7):1119-1125.

26. Hoffmann TJ, Kvale MN, Hesselson SE, Zhan Y, Aquino C, Cao Y, Cawley S, Chung E, Connell S, Eshragh J *et al*: **Next generation genome-wide association tool: design and coverage of a high-throughput European-optimized SNP array**. *Genomics* 2011, **98**(2):79-89.

27. Gorrindo P, Lane CJ, Lee EB, McLaughlin B, Levitt P: **Enrichment of elevated plasma F2t-isoprostane levels in individuals with autism who are stratified by presence of gastrointestinal dysfunction**. *PloS one* 2013, **8**(7):e68444.

28. Risi S, Lord C, Gotham K, Corsello C, Chrysler C, Szatmari P, Cook EH, Jr., Leventhal BL, Pickles A: **Combining information from multiple sources in the diagnosis of autism spectrum disorders**. *Journal of the American Academy of Child and Adolescent Psychiatry* 2006, **45**(9):1094-1103.

29. Borglum AD, Demontis D, Grove J, Pallesen J, Hollegaard MV, Pedersen CB, Hedemand A, Mattheisen M, investigators G, Uitterlinden A *et al*: **Genome-wide study of association and interaction with maternal cytomegalovirus infection suggests new schizophrenia loci**. *Molecular psychiatry* 2014, **19**(3):325-333.

30. Hollegaard MV, Grove J, Grauholm J, Kreiner-Moller E, Bonnelykke K, Norgaard M, Benfield TL, Norgaard-Pedersen B, Mortensen PB, Mors O *et al*: **Robustness of genome-wide scanning using archived dried blood spot samples as a DNA source**. *BMC Genet* 2011, **12**:58.

31. Schendel DE, Diguiseppi C, Croen LA, Fallin MD, Reed PL, Schieve LA, Wiggins LD, Daniels J, Grether J, Levy SE *et al*: **The Study to Explore Early Development (SEED): a multisite epidemiologic study of autism by the Centers for Autism and Developmental Disabilities Research and Epidemiology (CADDRE) network**. *Journal of autism and developmental disorders* 2012, **42**(10):2121-2140.

32. Weiss LA, Shen Y, Korn JM, Arking DE, Miller DT, Fossdal R, Saemundsen E, Stefansson H, Ferreira MA, Green T *et al*: **Association between microdeletion and microduplication at 16p11.2 and autism**. *N Engl J Med* 2008, **358**(7):667-675.

33. Steinberg S, de Jong S, Mattheisen M, Costas J, Demontis D, Jamain S, Pietilainen OP, Lin K, Papiol S, Huttenlocher J *et al*: **Common variant at 16p11.2 conferring risk of psychosis**. *Molecular psychiatry* 2014, **19**(1):108-114.

34. Rutter ML, Bailey AJ, Berument SK, Lord C, Pickles A: **Social Communication Questionairre**. In*.* Los Angeles, CA: Western Psychological Services; 2003.

35. Patterson N, Price AL, Reich D: **Population structure and eigenanalysis**. *PLoS genetics* 2006, **2**(12):e190.

36. Cross-Disorder Group of the Psychiatric Genomics Consortium P: **Identification of risk loci with shared effects on five major psychiatric disorders: a genome-wide analysis**. *Lancet* 2013, **381**(9875):1371-1379.

37. Spielman RS, McGinnis RE, Ewens WJ: **Transmission test for linkage disequilibrium: the insulin gene region and insulin-dependent diabetes mellitus (IDDM)**. *American journal of human genetics* 1993, **52**(3):506-516.

38. Schaid DJ: **General score tests for associations of genetic markers with disease using cases and their parents**. *Genetic epidemiology* 1996, **13**(5):423-449.

39. Lange C, Laird NM: **Power calculations for a general class of family-based association tests: dichotomous traits**. *American journal of human genetics* 2002, **71**(3):575-584.

40. Howie B, Marchini J, Stephens M: **Genotype imputation with thousands of genomes**. *G3 (Bethesda)* 2011, **1**(6):457-470.

41. Delaneau O, Marchini J, Zagury JF: **A linear complexity phasing method for thousands of genomes**. *Nature methods* 2012, **9**(2):179-181.

42. Purcell S, Neale B, Todd-Brown K, Thomas L, Ferreira MA, Bender D, Maller J, Sklar P, de Bakker PI, Daly MJ *et al*: **PLINK: a tool set for whole-genome association and population-based linkage analyses**. *American journal of human genetics* 2007, **81**(3):559-575.

43. Chang CC, Chow CC, Tellier LC, Vattikuti S, Purcell SM, Lee JJ: **Second-generation PLINK: rising to the challenge of larger and richer datasets**. *Gigascience* 2015, **4**:7.

44. Devlin B, Roeder K: **Genomic control for association studies**. *Biometrics* 1999, **55**(4):997-1004.

45. Pe'er I, Yelensky R, Altshuler D, Daly MJ: **Estimation of the multiple testing burden for genomewide association studies of nearly all common variants**. *Genetic epidemiology* 2008, **32**(4):381-385.

46. de Bakker PI, Ferreira MA, Jia X, Neale BM, Raychaudhuri S, Voight BF: **Practical aspects of imputation-driven meta-analysis of genome-wide association studies**. *Human molecular genetics* 2008, **17**(R2):R122-128.

47. Willer CJ, Li Y, Abecasis GR: **METAL: fast and efficient meta-analysis of genomewide association scans**. *Bioinformatics* 2010, **26**(17):2190-2191.

48. Myers AJ, Gibbs JR, Webster JA, Rohrer K, Zhao A, Marlowe L, Kaleem M, Leung D, Bryden L, Nath P *et al*: **A survey of genetic human cortical gene expression**. *Nature genetics* 2007, **39**(12):1494-1499.

49. Xia K, Shabalin AA, Huang S, Madar V, Zhou YH, Wang W, Zou F, Sun W, Sullivan PF, Wright FA: **seeQTL: a searchable database for human eQTLs**. *Bioinformatics* 2012, **28**(3):451-452.

50. Neph S, Kuehn MS, Reynolds AP, Haugen E, Thurman RE, Johnson AK, Rynes E, Maurano MT, Vierstra J, Thomas S *et al*: **BEDOPS: high-performance genomic feature operations**. *Bioinformatics* 2012, **28**(14):1919-1920.

51. Bulik-Sullivan BK, Loh PR, Finucane HK, Ripke S, Yang J, Schizophrenia Working Group of the Psychiatric Genomics C, Patterson N, Daly MJ, Price AL, Neale BM: **LD Score regression distinguishes confounding from polygenicity in genome-wide association studies**. *Nature genetics* 2015, **47**(3):291-295.

52. Stahl EA, Raychaudhuri S, Remmers EF, Xie G, Eyre S, Thomson BP, Li Y, Kurreeman FA, Zhernakova A, Hinks A *et al*: **Genome-wide association study meta-analysis identifies seven new rheumatoid arthritis risk loci**. *Nature genetics* 2010, **42**(6):508-514.

53. Huang H, Chanda P, Alonso A, Bader JS, Arking DE: **Gene-based tests of association**. *PLoS genetics* 2011, **7**(7):e1002177.

54. Arking DE, Pulit SL, Crotti L, van der Harst P, Munroe PB, Koopmann TT, Sotoodehnia N, Rossin EJ, Morley M, Wang X *et al*: **Genetic association study of QT interval highlights role for calcium signaling pathways in myocardial repolarization**. *Nature genetics* 2014, **46**(8):826-836.

55. Trynka G, Hunt KA, Bockett NA, Romanos J, Mistry V, Szperl A, Bakker SF, Bardella MT, Bhaw-Rosun L, Castillejo G *et al*: **Dense genotyping identifies and localizes multiple common and rare variant association signals in celiac disease**. *Nature genetics* 2011, **43**(12):1193-1201.

56. Mishra A, Macgregor S: **VEGAS2: Software for More Flexible Gene-Based Testing**. *Twin Res Hum Genet* 2015, **18**(1):86-91.

57. Wojcik GL, Kao WH, Duggal P: **Relative performance of gene- and pathway-level methods as secondary analyses for genome-wide association studies**. *BMC Genet* 2015, **16**:34.

58. De Rubeis S, He X, Goldberg AP, Poultney CS, Samocha K, Cicek AE, Kou Y, Liu L, Fromer M, Walker S *et al*: **Synaptic, transcriptional and chromatin genes disrupted in autism**. *Nature* 2014, **515**(7526):209-215.

59. Sanders SJ, He X, Willsey AJ, Ercan-Sencicek AG, Samocha KE, Cicek AE, Murtha MT, Bal VH, Bishop SL, Dong S *et al*: **Insights into Autism Spectrum Disorder Genomic Architecture and Biology from 71 Risk Loci**. *Neuron* 2015, **87**(6):1215-1233.

60. Darnell JC, Van Driesche SJ, Zhang C, Hung KY, Mele A, Fraser CE, Stone EF, Chen C, Fak JJ, Chi SW *et al*: **FMRP stalls ribosomal translocation on mRNAs linked to synaptic function and autism**. *Cell* 2011, **146**(2):247-261.

61. Sugathan A, Biagioli M, Golzio C, Erdin S, Blumenthal I, Manavalan P, Ragavendran A, Brand H, Lucente D, Miles J *et al*: **CHD8 regulates neurodevelopmental pathways associated with autism spectrum disorder in neural progenitors**. *Proceedings of the National Academy of Sciences of the United States of America* 2014, **111**(42):E4468-4477.

62. Iossifov I, O'Roak BJ, Sanders SJ, Ronemus M, Krumm N, Levy D, Stessman HA, Witherspoon KT, Vives L, Patterson KE *et al*: **The contribution of de novo coding mutations to autism spectrum disorder**. *Nature* 2014, **515**(7526):216-221.

63. Uddin M, Tammimies K, Pellecchia G, Alipanahi B, Hu P, Wang Z, Pinto D, Lau L, Nalpathamkalam T, Marshall CR *et al*: **Brain-expressed exons under purifying selection are enriched for de novo mutations in autism spectrum disorder**. *Nature genetics* 2014, **46**(7):742-747.

64. Parikshak NN, Luo R, Zhang A, Won H, Lowe JK, Chandran V, Horvath S, Geschwind DH: **Integrative functional genomic analyses implicate specific molecular pathways and circuits in autism**. *Cell* 2013, **155**(5):1008-1021.

65. Schizophrenia Working Group of the Psychiatric Genomics Consortium: **Biological insights from 108 schizophrenia-associated genetic loci**. *Nature* 2014, **511**(7510):421-427.

66. Samocha KE, Robinson EB, Sanders SJ, Stevens C, Sabo A, McGrath LM, Kosmicki JA, Rehnstrom K, Mallick S, Kirby A *et al*: **A framework for the interpretation of de novo mutation in human disease**. *Nature genetics* 2014, **46**(9):944-950.

67. Lindblad-Toh K, Garber M, Zuk O, Lin MF, Parker BJ, Washietl S, Kheradpour P, Ernst J, Jordan G, Mauceli E *et al*: **A high-resolution map of human evolutionary constraint using 29 mammals**. *Nature* 2011, **478**(7370):476-482.

68. Xu K, Schadt EE, Pollard KS, Roussos P, Dudley JT: **Genomic and network patterns of schizophrenia genetic variation in human evolutionary accelerated regions**. *Molecular biology and evolution* 2015.

69. Subramanian A, Tamayo P, Mootha VK, Mukherjee S, Ebert BL, Gillette MA, Paulovich A, Pomeroy SL, Golub TR, Lander ES *et al*: **Gene set enrichment analysis: a knowledge-based approach for interpreting genome-wide expression profiles**. *Proceedings of the National Academy of Sciences of the United States of America* 2005, **102**(43):15545-15550.
